# Enhancing Treg persistence by inhibiting necroptosis restrains autoimmune pathology

**DOI:** 10.1172/JCI207077

**Published:** 2026-09-01

**Authors:** Qiaoyan Wu, Na Cui, Li Gao, Zhihui Lu, Xiang Ao, Rui Pang, Xingyan Li, Heling Pan, Daichao Xu, Peiying Li, Junying Yuan, Chengyu Zou

**Affiliations:** 1 Interdisciplinary Research Center on Biology and Chemistry, Shanghai Institute of Organic Chemistry, Chinese Academy of Sciences, Shanghai, China.; 2 University of Chinese Academy of Sciences, Beijing, China.; 3 Department of Neurology, and; 4 Department of Anesthesiology, Key Laboratory of the Ministry of Education, Renji Hospital, Shanghai Jiao Tong University School of Medicine, Shanghai, China.; 5 Clinical Research Center, Renji Hospital, Shanghai Jiao Tong University School of Medicine, Shanghai, China.; 6 Shanghai Key Laboratory of Aging Studies, Shanghai, China.

**Keywords:** Autoimmunity, Cell biology, Adaptive immunity, Tregs

## Abstract

Regulatory T cells (Tregs) hold great promise as next-generation therapeutics for autoimmune diseases. However, maintaining their functional persistence within inflamed tissues remains a major translational challenge. Using an in vitro system that recapitulates the inflammatory CNS milieu of multiple sclerosis (MS), together with a pooled shRNA screen, we identify necroptotic signaling as a key driver of Treg death, thereby compromising Treg functional persistence under inflammatory conditions. We further demonstrate that Tregs in both a mouse model of MS and patients with MS exhibited a preferential susceptibility to receptor-interacting serine/threonine kinase 1–dependent (RIPK1-dependent) necroptosis. Mechanistically, a FOXP3-driven, low-glucose metabolic program rendered Tregs intrinsically susceptible to necroptosis by limiting O-GlcNAcylation (O-GlcNAc) modification on RIPK1. This vulnerability was not shared by conventional T cells under comparable inflammatory conditions. Finally, in combination with adoptive Treg transfer, we show that selective inhibition of necroptosis in Tregs enhanced their survival and suppressive function at sites of active inflammation, thereby reducing autoimmune pathology in mouse models of MS and systemic lupus erythematosus. Together, these findings identify necroptotic cell death as a barrier to Treg persistence within inflamed tissues and highlight the therapeutic potential of necroptosis-resistant Tregs for the treatment of autoimmune diseases.

## Abstract 2

Protecting immune-regulating cells from a form of inflammatory cell death helps them survive and better control autoimmune disease in multiple sclerosis and lupus

## Graphical abstract

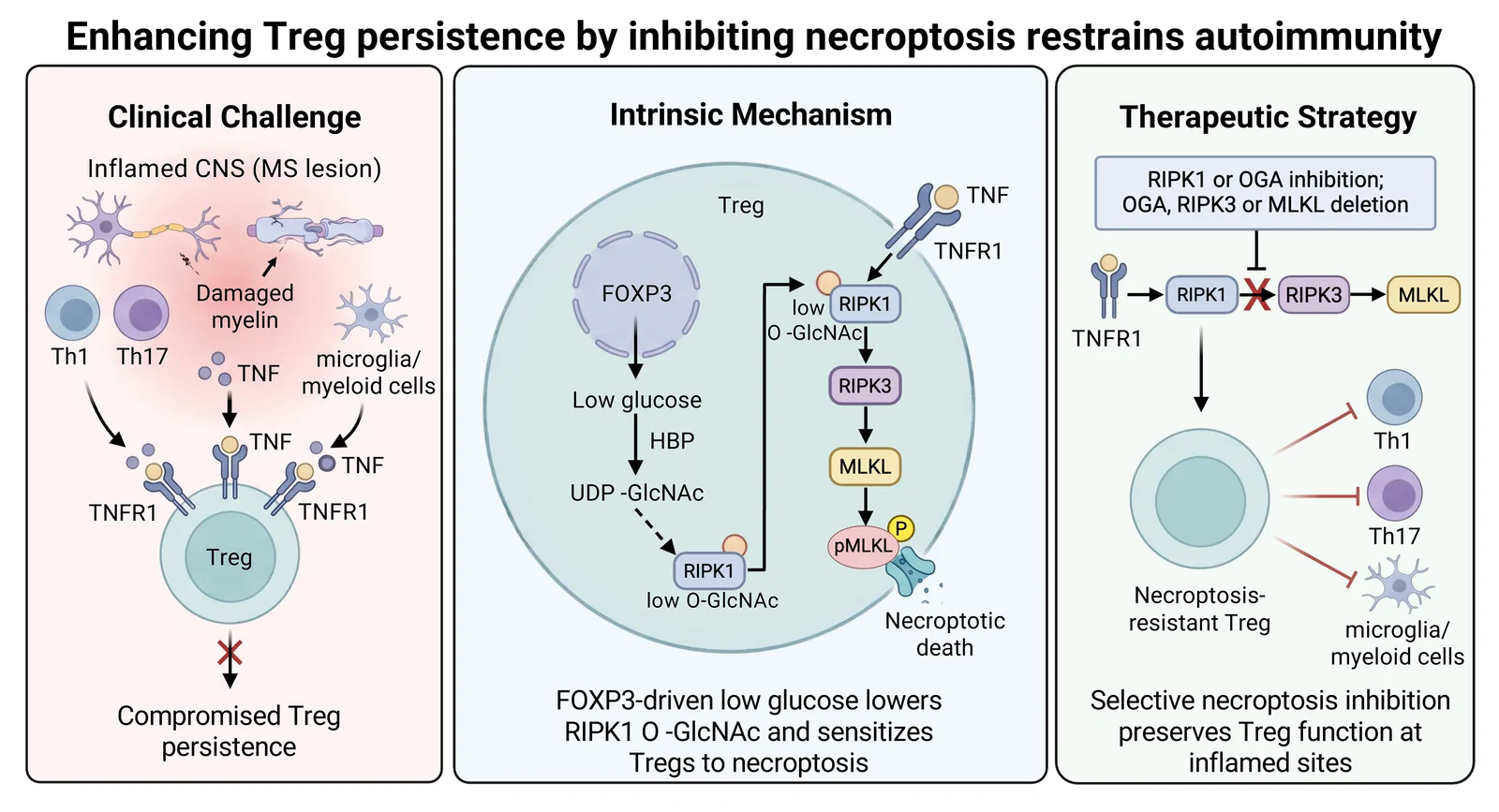

## Introduction

Defects in regulatory T cell–mediated (Treg-mediated) immunosuppression are frequently implicated in autoimmune diseases such as multiple sclerosis (MS) and systemic lupus erythematosus (SLE), particularly within inflamed tissues (1–3). Current strategies to enhance Treg-based immunotherapies for autoimmune diseases include in vivo expansion using low-dose IL-2 or TNFR2 agonists, enforced FOXP3 expression through genetic manipulation, and antigen-specific or CAR engineering (4–11). While some of these approaches have yielded encouraging outcomes in early clinical trials, the signaling pathways that compromise Treg functional persistence at sites of active inflammation, defined here as reduced survival together with impaired suppressive capacity at the cell population level, remain largely undefined.

Sites of active inflammation in autoimmune disease are characterized by the convergence of pathogenic effector T cells and activated myeloid cells, which together generate a cytokine- and metabolite-rich environment that challenges Treg survival and limits their suppressive capacity (12, 13). In MS, Th1 and Th17 (Th1/Th17) cells are dominant drivers of tissue inflammation through the initial production of IFN-γ, IL-17, and TNF, whereas activated myeloid cells amplify inflammatory signaling via antigen presentation, costimulatory cues, and additional cytokines and reactive mediators. This coordinated interaction between effector T cells and myeloid cells is a defining feature of inflammatory lesions. However, the mechanisms by which this inflammatory setting compromises Treg functional persistence, including survival and suppressive capacity, have not been systematically examined.

Tregs exhibit a distinct metabolic signature that distinguishes them from conventional CD4^+^ T cells (Tcons). Unlike Tcons, which rely heavily on glucose metabolism, Tregs exhibit reduced glucose uptake and lower expression of glucose transporters (14–16). While deficiencies in glucose transporters impair Tcon proliferation, they do not affect Treg function or expansion (17, 18). This low-glucose metabolic signature is regulated by FOXP3, which reprograms Treg metabolism to sustain function in low-glucose environments (15, 19, 20). Paradoxically, elevated glucose levels have been shown to inhibit Treg-suppressive capacity (21).

Necroptosis, characterized by the sequential activation of receptor-interacting serine/threonine kinase 1 (RIPK1), RIPK3, and mixed lineage kinase domain-like protein (MLKL), is recognized as a proinflammatory form of regulated cell death in innate immune and parenchymal cells, where it serves as an evolutionarily conserved defense mechanism that amplifies immune responses and promotes pathogen clearance (22, 23). However, genetic mutations that enhance necroptotic signaling are associated with a wide spectrum of inflammatory and autoimmune diseases (24). For instance, noncleavable variants of RIPK1 lead to a dominant autoinflammatory disease, and haploinsufficiency of the necroptosis checkpoint A20 results in an early onset autoinflammatory disease (25, 26). Consequently, developing strategies to selectively modulate necroptosis in vivo in a therapeutically tractable manner, without broadly suppressing this pathway, remains a major unresolved challenge.

## Results

### Tregs restrain Th1- and Th17-driven inflammation yet undergo profound cell death.

To mimic the inflammatory characteristics of the MS CNS, we developed a system with coculturing of in vitro–primed Th1/Th17 cells from experimental autoimmune encephalomyelitis (EAE) mice and BV2 microglia (Supplemental Figure 1, A and B; supplemental material available online with this article; https://doi.org/10.1172/JCI207077DS1). Compared with naive CD4^+^ T cells, Th1/Th17 cells markedly altered the gene expression profile of BV2 microglia (Supplemental Figure 1, C and D). Kyoto Encyclopedia of Genes and Genomes (KEGG) pathway analysis of upregulated genes showed enrichment of the immune disease–related pathway, particularly SLE (Supplemental Figure 1E and Supplemental Table 1). Because the KEGG does not include an MS-specific pathway, we generated a curated list of MS-associated microglial signatures based on previous publications (Supplemental Table 2) (27, 28). We observed significant enrichment of this MS microglial signature in the Th1/Th17-BV2 coculture system, but not in the naive CD4^+^ T cell-BV2 coculture system (Supplemental Figure 1F). By quantitative PCR (qPCR), we further confirmed markedly increased proinflammatory cytokines and mediators (*Il1a*, *Il1b*, *Ptgs2*, *Nos2*, and *Tnf*), upregulated the activated microglia marker *Cd86*, and reduced the homeostatic marker *Tmem119* (Supplemental Figure 1G). These molecular signatures were consistent with CNS inflammation in MS (29–32). We introduced natural Tregs into the Th1/Th17-BV2 coculture system and found that they effectively suppressed inflammatory responses in a dose-dependent manner (Supplemental Figure 1H).

However, at sites of active inflammation, such as the MS CNS, maintaining Treg functional persistence remains a major challenge for the clinical translation of Treg-based therapies. To determine whether this inflammatory milieu compromises Treg survival or suppressive capacity, we took advantage of the Th1/Th17-BV2 coculture system. Treg functional persistence was assessed by measuring Treg death and the expression of FOXP3 and CD25 in surviving Tregs. We used a 50:1 microglia/Treg ratio based on initial titration experiments (Supplemental Figure 1H), which provided an optimal dynamic range while allowing sufficient recovery of Tregs by sorting for downstream molecular analyses. CFSE-prelabeled Tregs were added to cocultures containing Th1/Th17 cells and BV2 microglia for 48 hours. Under these conditions, we observed substantial Treg death, along with a slight but statistically insignificantly downward trend in FOXP3 and CD25 expression among the surviving Tregs (Supplemental Figure 2, A and B).

To further explore the temporal dynamics of Treg instability, we introduced Tregs into the Th1/Th17-BV2 coculture for 6, 12, 24, and 48 hours. Treg death increased progressively over time, whereas FOXP3 expression remained relatively stable in the surviving cells throughout the time course (Supplemental Figure 2C). Treg death in the naive CD4-BV2 coculture system was relatively mild, suggesting that Treg death was largely driven by the inflammatory environment rather than by BV2 microglia alone or the in vitro culture system itself (Supplemental Figure 2C). In addition, we found that Th1/Th17-activated BV2 microglia did not promote Treg proliferation (Supplemental Figure 2D). Instead, additional CD3/CD28 stimulation induced Treg proliferation in the coculture system, as indicated by increased Ki67 expression (Supplemental Figure 2E), suggesting that Treg proliferation and Treg death were driven by distinct signals originating from TCR stimulation and inflammatory responses, respectively.

Collectively, these results indicate that impaired Treg functional persistence at sites of active inflammation is driven primarily by Treg death rather than by FOXP3 downregulation.

### Functional genetic screen identifies necroptotic signaling as a key driver limiting Treg functional persistence at sites of active inflammation.

To identify the molecular basis underlying the regulation of Treg functional persistence at sites of active inflammation, we developed a pooled shRNA library targeting 2,232 genes spanning major signaling pathways (6 distinct shRNA sequences per gene; see the gene list in Supplemental Table 3). This shRNA library was introduced into mouse natural Tregs using a lentiviral vector coexpressing EGFP, with the multiplicity of infection kept below 0.1 to ensure single shRNA integration per cell. EGFP^+^ Tregs were sorted 72 hours after transduction, yielding, on average, more than 200 cells per shRNA and were subsequently transferred into the Th1/Th17-BV2 coculture system for the next 48 hours using a Treg/microglia ratio of 1:50. Naive CD4-BV2 coculture system served as a control (Figure 1A).

As functional Tregs express high levels of CD25, we isolated Live/CD25^hi^/EGFP^+^ Tregs by flow cytometry to identify genes that regulate Treg functional persistence within the Th1/Th17-BV2 coculture system (Supplemental Figure 3). Our screen identified *Xiap*, *Il10*, and *Ctla4* as genes required to maintain Treg functional persistence in this system (Figure 1B and Supplemental Table 4). Loss of XIAP promotes RIPK1- and RIPK3-dependent necroptosis in multiple immune cell types, including neutrophils and DCs (33, 34) and specially impairs the suppressive function of Tregs (35). Furthermore, IL-10 and CTLA-4 are well-established immunoregulatory mediators required for optimal Treg suppressive function (36, 37). To validate whether loss of IL-10 or CTLA-4 would contribute to compromised Treg functional persistence, we delivered an siRNA targeting *Il10* or *Ctla4* into Tregs using a coacervate-based delivery system, which achieved more than 80% transfection efficiency (Supplemental Figure 2F) (38). Knockdown of *Il10* or *Ctla4* significantly reduced the ability of Tregs to suppress inflammatory responses and increased Treg death under inflammatory conditions (Figure 1, C and D, and Supplemental Figure 2G). The identification of *Xiap*, *Il10*, and *Ctla4*, three well-established modulators of Treg survival and suppressive function, confirmed the robustness and validity of our screening approach.

We next examined genes whose knockdown increased the recovery of functionally persistent Tregs in our screen. In total, we identified 29 genes whose loss led to functional Treg enrichment within the Th1/Th17-BV2 coculture system. *Ripk3* emerged as the top target (Figure 1B, and Supplemental Table 4). We then delivered siRNAs targeting each gene into Tregs and found that *Ripk3* knockdown most strongly enhanced the suppressive ability of Tregs against Th1/Th17-triggered inflammation compared with negative control–transfected (siNC-transfected) controls (Figure 1E).

As a key component of the necroptotic signaling pathway, RIPK3 can be activated by RIPK1 to form the necrosome and subsequently activate MLKL, which disrupts lysosomal, mitochondrial, and plasma membranes to trigger cell death (22). The involvement of necroptotic signaling in Treg persistence and function has not been previously examined.

To further determine whether activation of the necroptotic pathway compromises the functional persistence of both natural and induced Tregs at sites of active inflammation, we isolated natural Tregs or in vitro–induced Tregs from RIPK1-D138N (kinase-inactive RIPK1 mutant) and MLKL-KO mice (Supplemental Figure 4A). We found that inhibition of RIPK1 kinase activity or deletion of MLKL efficiently suppressed inflammatory responses and markedly reduced cell death in both Treg populations (Figure 1, F–I). Similarly, using natural and in vitro–induced Tregs isolated from RIPK3-KO mice, we confirmed that genetic inhibition of necroptosis enhanced Treg-mediated suppression of inflammatory responses and markedly reduced Treg death in the Th1/Th17-primary microglia coculture system (Supplemental Figure 4, B–F).

Furthermore, we used a flow cytometry–based analysis we previously established to detect the activated necroptotic markers phosphorylated (S166) RIPK1 (pRIPK1), phosphorylated (T231/232) RIPK3 (pRIPK3), and phosphorylated (S345) MLKL (pMLKL) in Tregs within the Th1/Th17-BV2 coculture system (39). The specificity of these phospho-antibodies for flow cytometric detection was further validated (Supplemental Figure 5A). These analyses confirmed that necroptotic signaling was actively engaged in Tregs at sites of active inflammation (Supplemental Figure 5B).

Taken together, these results demonstrate that activation of necroptotic signaling was a primary driver of Treg death and compromised functional persistence at sites of active inflammation.

### Preventing Treg necroptosis ameliorates EAE pathology.

We next evaluated whether inhibiting Treg necroptosis could mitigate autoimmune pathology in vivo. We established a passive EAE mouse model by introducing in vitro–primed Th1/Th17 cells into RAG2-KO mice. Naive CD4^+^ T cells isolated from WT, RIPK1-D138N, or MLKL-KO mice were polarized in vitro into Tregs. Subsequently, an equivalent number of Tregs from each genotype were cotransferred with antigen-activated Th1/Th17 cells into RAG2-KO mice (Figure 2A). Although WT Tregs effectively reduced EAE clinical scores as a result of the primed Th1/Th17 cells, transfer of RIPK1-D138N or MLKL-KO Tregs led to even greater improvement in EAE symptoms (Figure 2A). In an independent experiment, we also observed similar protection following transfer of RIPK3-KO Tregs (Supplemental Figure 6A). Moreover, genetic inhibition of the necroptotic pathway in these mouse models did not affect baseline Treg homeostasis (Supplemental Figure 6, B–E), consistent with previous reports (40, 41).

We also found that inhibition of Treg necroptosis decreased Th1/Treg and Th17/Treg ratios in the CNS of EAE mice (Figure 2B, and Supplemental Figure 6, F–H, and Supplemental Figure 7A). A reduction in demyelination, as demonstrated by an increase in myelin basic protein (MBP) staining, was also observed in the RIPK1-D138N, RIPK3-KO, and MLKL-KO Treg EAE groups as compared with those treated with WT Tregs (Figure 2C and Supplemental Figure 7, B and C). The attenuated activation of microglia and astrocytes coincided with decreased expression of inflammatory cytokines in the spinal cord of EAE mice upon inhibition of Treg necroptosis (Figure 2, D–G, and Supplemental Figure 7, D and E, and Supplemental Figure 8). Taken together, these data indicate that inhibition of Treg necroptosis effectively prevented the development of EAE.

To further examine whether Treg necroptosis contributes to compromised Treg functional persistence during EAE pathogenesis, we transferred WT CD45.1^+^ Tregs and MLKL-KO CD45.2^+^ Tregs at a 1:1 ratio into RAG2-KO mice, together with primed Th1/Th17 cells. Th1/Th17 cells were isolated from EGFP mice for the purpose of distinguishing them from Tregs. Two weeks after administration, the equal ratio of CD45.1^+^EGFP^–^/CD45.2^+^EGFP^–^ Tregs was conserved in control RAG2-KO mice, whereas it was reduced during the development of EAE (Figure 2H, and Supplemental Figure 9, A and B). Ki-67 staining showed that MLKL deficiency did not affect Treg proliferation (Supplemental Figure 9C). These data suggest that necroptosis drives significant Treg loss during EAE pathogenesis.

Given that the pathological conversion of Tregs into Th17 cells has been recognized in autoimmune disorders (42), we examined the ratios of CD45.1^+^EGFP^–^ Th17 cells to CD45.1^+^EGFP^–^ Tregs and of CD45.2^+^EGFP^–^ Th17 cells to CD45.2^+^EGFP^–^ Tregs in passive EAE mice as outlined in the preceding paragraph. The comparison of them showed no significant difference 2 weeks after T cell transfer (Supplemental Figure 9D). These results suggest that necroptosis of Tregs does not affect EAE pathogenesis through Treg/Th17 conversion.

### Tregs are preferentially susceptible to necroptosis in EAE mice and patients with MS.

Next, we sought to determine whether Tregs indeed undergo necroptosis during the development of autoimmunity. CNS-infiltrated immune cells, including T cells, have been shown to exit the CNS and reenter the circulation during MS pathogenesis (43–46). We assessed necroptotic signaling in Tregs isolated from the blood of EAE mice and patients with MS.

Mice were injected with myelin oligodendrocyte glycoprotein (MOG)_35–55_/CFA and pertussis toxin (PTX) to induce active EAE. Before immunization, the levels of pRIPK1, pRIPK3, and pMLKL were comparable across all CD4^+^ T cell subsets in both WT and RIPK1 D138N mice, indicating the absence of detectable necroptotic pathway activation before disease induction. However, as EAE progressed, pRIPK1, pRIPK3, and pMLKL levels became preferentially elevated in Tregs, but not in Th1, Th2, or Th17 subsets (Supplemental Figure 10, A and B). In addition, the presence of pMLKL in Tregs was associated with an increase in SYTOX staining, indicating the disruption of plasma membrane (Supplemental Figure 10B).

To further confirm that Tregs are vulnerable to necroptosis during EAE, we utilized a FOXP3 reporter mouse model and induced EAE pathogenesis. In this system, Tregs were identified as CD4^+^ T cells expressing FOXP3, while Tcons were CD4^+^FOXP3^–^. Using imaging flow cytometry (IFC), we detected aggregation of RIPK1 and MLKL, which are also markers of RIPK1 activation and necroptosis (47), in Tregs. We also observed increased expression of pRIPK1 and pMLKL in Tregs, but not in Tcons, during EAE pathogenesis (Figure 3, A and B, and Supplemental Figure 11, A and B). In addition, staining for pRIPK1 and pMLKL in spinal cord sections from EAE mice revealed that a substantial proportion of Tregs showed activated necroptotic signaling (Figure 4, A and B). Together, these data indicate that Tregs are preferentially susceptible to RIPK1 activation and necroptosis during EAE pathology.

To explore RIPK1 activation and its downstream signaling pathways in leukocytes from patients with MS, we performed single-cell mass cytometry by time of flight (CyTOF) to examine peripheral blood samples from patients with MS and age-/sex-matched healthy controls (Supplemental Table 5). Leveraging the high-dimensional capabilities of CyTOF, we designed a panel of immune markers combined with RIPK1, pRIPK1, pMLKL, pS176/S180 IKK (pIKK), and cleaved caspase 3 (cCasp3) (Figure 5A). pIKK and cCasp3 are indicators of NF-κB inflammatory pathway activation and apoptosis, respectively. The suitability of these antibodies for flow cytometric analysis has been assessed by the manufacturers. We identified Tregs according to surface markers (CD66b^–^CD11b^–^CD3^+^CD4^+^CD8–CD25^+^CD127^–^) and distinguished other PBMC populations, including Th1, Th2, and Th17 cells, CD8^+^ T cells, B cells, monocytes, NK cells, plasmacytoid DCs (pDCs), and conventional DCs (cDCs) (Supplemental Figure 12A). Although the proportion of Tregs in patients with MS showed a downward trend compared with the control group, the difference was not statistically significant (average 4.3% vs. 5.6%, *P* = 0.1624, Supplemental Figure 12B). However, the Th1/Treg and Th17/Treg ratios were significantly elevated in patients with MS compared with healthy controls (Figure 5B), indicating disrupted immune homeostasis, consistent with previous reports (48, 49).

In line with the increased susceptibility to necroptosis observed in Tregs of EAE mice, we found significantly higher expression levels of pRIPK1 and pMLKL, the terminal effector of necroptosis, in Tregs from patients with MS (Figure 5C). However, the expression levels of cCasp3, pIKK, and RIPK1 were comparable between patients with MS and individuals in the control group (Supplemental Figure 12, C–E). These findings suggest that Tregs in patients with MS selectively exhibit activation of the RIPK1/MLKL necroptotic pathway.

Importantly, elevated levels of pRIPK1, pMLKL, pIKK, and cCasp3 were not detected in other PBMC populations (Supplemental Figure 13, A–E). Using RIPK1 signaling as an internal control, we further demonstrated that Tregs, but not other PBMC populations, exhibited RIPK1 activation in patients with MS, as evidenced by an increased pRIPK1/RIPK1 ratio (Figure 5D and Supplemental Figure 13, F–H). These findings suggest that Tregs in patients with MS undergo RIPK1 activation and necroptosis.

Using the Th1/Th17-BV2 microglia coculture system established above, we further confirmed that human Tregs exhibited markedly greater susceptibility to RIPK1/MLKL necroptotic pathway activation than did human Tcons (Supplemental Figure 14). Thus, while our findings support a conserved vulnerability of Tregs to RIPK1/MLKL necroptotic signaling in MS-related inflammation, the direct relevance of this pathway to Treg behavior within the human CNS will need to be investigated in future studies.

### TNF induces Treg necroptosis.

One of the most extensively studied signal transduction processes that activates RIPK1 and triggers necroptosis is the soluble TNF/TNFR1 signaling pathway, which is also a curial mediator in EAE pathogenesis (50). We next examined whether TNF could induce necroptosis of Tregs. A nonlethal dose of soluble TNF (4 μg/mouse) was administered by i.v. injection, and circulating immune cells were collected 2 hours after injection. Consistent with the preferential susceptibility of necroptosis in Tregs during EAE pathogenesis, and as seen patients with MS, Tregs had elevated expression levels of necroptotic markers in comparison with other CD4^+^ T cell subsets following TNF treatment (Supplemental Figure 15). We further assessed the susceptibility of TNF-induced necroptosis using FOXP3 reporter mice. Using IFC, we detected significantly higher expression of pRIPK1, pRIPK3, and pMLKL staining in Tregs compared with Tcons upon TNF stimulation (Supplemental Figure 16A). The occurrence of pRIPK1, pRIPK3 and pMLKL in Tregs was linked to an increase of SYTOX stain (Supplemental Figure 16B). These findings collectively suggest that Tregs have heightened vulnerability to necroptosis in the presence of TNF in vivo.

To examine the susceptibility of Tregs to necroptosis upon TNF stimulation in vitro, we differentiated Th1 and Tregs from naive CD4^+^ T cells. A lactate dehydrogenase (LDH) cytotoxicity assay demonstrated that soluble TNF treatment led to a significant loss of Tregs rather than Th1 cells (Supplemental Figure 16C). The TNF-induced cell death in Tregs could be prevented by inhibitors of RIPK1 and RIPK3 kinases, indicating the involvement of necroptosis (Supplemental Figure 16C). Furthermore, Western blot analysis detected the necroptotic markers pRIPK1, pRIPK3, and pMLKL in Tregs treated with TNF (Supplemental Figure 16D). These observations demonstrate that soluble TNF treatment renders Tregs susceptible to necroptosis in vitro.

### Reduced O-GlcNAcylation modification of RIPK1 contributes to the preferential vulnerability of Tregs to necroptosis.

Tregs are known to exhibit unique characteristics in terms of a low level of glucose uptake and metabolism (17). Consistent with earlier studies (17, 51), Glut1, an essential glucose transporter in T cells, exhibited diminished expression levels in both natural and in vitro–induced Tregs when compared with other CD4^+^ T cell subsets (Supplemental Figure 17, A–C). The effect of glucose uptake on regulating necroptosis has been revealed by our previous studies (39, 52, 53). Hence, we embarked on an investigation to determine whether the distinct glucose metabolism signature of Tregs plays a role in the vulnerability of Treg necroptosis in autoimmune pathogenesis.

Glucose-derived pyruvate could be metabolized into citrate in the mitochondria, which is then exported into cytoplasm and broken into acetyl-CoA. We have previously demonstrated that glucose-derived acetyl-CoA regulates the acetylation of A20 protein, thereby preventing its lysosomal degradation (39, 52). A20 protects cells from necroptosis via regulation of the ubiquitination of RIPK1 and RIPK3 (54). Considering these findings, we hypothesized that Tregs might potentially downregulate A20, rendering them susceptible to necroptosis. However, the level of A20 expression in Tregs was, in fact, comparable to that in other CD4^+^ T cell subsets (Supplemental Figure 17, D–F).

With reduced glucose uptake, Tregs also exhibit increased pyruvate oxidation (51). This feature may help maintain pyruvate and citrate concentrations in Tregs at levels comparable to those in Th1/Th17 cells, despite low glucose uptake, while further diminishing glucose flux into other glycolytic pathways (51). In addition to glycolysis catalyzing the conversion of glucose to pyruvate, glucose may concurrently undergo downstream metabolism through the hexosamine biosynthetic pathway (HBP) (Supplemental Figure 17G). Indeed, in Tregs, we observed significant downregulation of HBP metabolites, including UDP-GlcNAc, which acts as the donor substrate for *O-GlcNAcylation* (O-GlcNAc) (Supplemental Figure 17G; data were sourced from the supplemental materials of Gerriets et al.; ref. 51).

Consistent with the reduced activity of HBP, O-GlcNAc was attenuated in Tregs (Supplemental Figure 17, H and I). As O-GlcNAc prevents necroptosis by targeting both RIPK1 and RIPK3 (55, 56), we proceeded to investigate the O-GlcNAc levels of RIPK1 and RIPK3 in Tregs. Our results demonstrated a substantial decrease in RIPK1 O-GlcNAc levels in Tregs, as evidenced by immunoprecipitation (IP) (showing a 64.2% decrease compared with Tcons, Figure 6A) or the proximity ligation assay (PLA) (showing a 63.55% decrease compared with Tcons, Figure 6, B and C). The specificity of the PLA signal was validated (Supplemental Figure 18A). Furthermore, the O-GlcNAc modification of RIPK3 in Tregs exhibited a relatively modest yet significant reduction (36.36% decrease by IP and 46.69% decrease by PLA, Supplemental Figure 18, B–D). Considering that the O-GlcNAc levels of RIPK1 were more markedly reduced in Tregs and that RIPK1 functions as an upstream regulator of RIPK3 in necroptosis, we next explored whether the decreased O-GlcNAc modification of RIPK1 in Tregs plays a role in their preferential vulnerability.

O-GlcNAc was added by O-GlcNAc transferase (OGT) and reversibly removed by O-GlcNAcase (OGA). Thiamet G (TMG) is a potent OGA inhibitor used to enhance O-GlcNAc both in vitro and in vivo (57). We examined the effect of TMG on O-GlcNAc levels in Tregs isolated from the spleen, and our findings demonstrated that TMG treatment could effectively increase O-GlcNAc in Tregs (Supplemental Figure 18E). In vivo administration of TMG did not change the frequency of Tregs, and Tregs treated with TMG retained their ability to suppress Th1 cells (Supplemental Figure 18, F and G). Notably, O-GlcNAc levels of RIPK1 and RIPK3 in Tregs significantly increased following TMG treatment, reaching levels comparable to those in Tcons (Figure 6D and Supplemental Figure 18H). These data suggest that the decreased O-GlcNAc levels of RIPK1 in Tregs can be effectively restored by inhibiting OGA without comprising its suppressive capabilities.

O-GlcNAc modification of RIPK1 at S332 suppresses its activation and subsequent necroptosis (56). Consistent with these findings, TNF treatment induced greater activation of RIPK1 and downstream necroptotic signaling in CD4^+^ effector T cells from RIPK1-S332A mice compared with WT controls (Figure 6, E and F). Importantly, the preferential vulnerability of Tregs to RIPK1 activation and necroptosis was abolished in RIPK1-S332A mice. Furthermore, treatment with TMG effectively reduced TNF-induced RIPK1 activation in Tregs from WT mice, but it had no effect in RIPK1-S332A mice (Figure 6E). Taken together, these results suggest that reduced O-GlcNAc modification of RIPK1 underlies the preferentially increased susceptibility of Tregs to RIPK1 activation and subsequent necroptosis.

### Treg necroptosis and EAE pathology are attenuated via the inhibition of OGA.

Previous studies have shown that TMG treatment or insufficient OGA activity mitigates cell necroptosis or necroptosis-related factors in vivo (55, 56, 58). Thus, we proceeded with an investigation into the potential effect of increasing O-GlcNAc levels on Treg necroptosis. Along with the elevation of O-GlcNAc levels on RIPK1 in Tregs induced by TMG administration, necroptosis of Tregs upon TNF treatment was significantly reduced (Supplemental Figure 19A). In addition, pretreatment of Tregs with TMG 24 hours prior to TNF exposure resulted in the successful prevention of Treg necroptosis (Supplemental Figure 19, B and C). These data collectively suggest that upregulation of O-GlcNAc via OGA inhibition serves as an effective interventional approach against TNF-induced Treg necroptosis both in vivo and in vitro.

Administration of TMG led to reduced disease severity, preserved myelin integrity, and alleviated neuroinflammation in mice with EAE (Supplemental Figure 19, D–F, and Supplemental Figure 20A). The observed therapeutic benefits of TMG treatment in EAE mice were correlated reduced ratios of Th1 cells/Tregs and Th17 cells/Tregs and a decline in Treg necroptosis (Supplemental Figure 20, B–E). Additionally, TMG treatment had a minimal effect on EAE progression in mice lacking Tregs (Supplemental Figure 20F). Collectively, these findings provide evidence that TMG treatment ameliorates EAE progression by inhibiting Treg necroptosis.

### Reduction of OGA in Tregs protects against EAE pathogenesis.

To further explore the effect of targeted elevation of O-GlcNAc in Tregs on the regulation of Treg necroptosis and disease progression in the context of EAE, we transduced Tregs with lentiviral vectors carrying an OGA shRNA to generate OGA-knockdown (KD) Tregs (shOGA Tregs) or a negative control shRNA Tregs (shNC Tregs). The decreased OGA expression in shOGA Tregs was sustained for at least 2 weeks in vitro (Supplemental Figure 21A). O-GlcNAc levels were significantly increased in shOGA Tregs, whereas expression levels of FOXP3 remained unchanged (Supplemental Figure 21B). Accordingly, shOGA Tregs exhibited elevated O-GlcNAc of RIPK1 (Supplemental Figure 21C). Expression levels of both IL-17A and IFN-γ in simulated shOGA Tregs were similar to those in untransduced Tregs and shNC Tregs (Supplemental Figure 21D). These data suggest that shOGA Tregs can maintain their suppressor phenotype in vitro.

Using the established passive EAE mouse model, we investigated the potential effect of shOGA Tregs on EAE development. In comparison with shNC Tregs, administration of shOGA Tregs had a superior protective effect on the severity of EAE induced by primed Th1/Th17 cells (Figure 7A). Preserved myelin integrity, reduced activation of astrocytes and microglia, reduced ratios of Th1 cells/Tregs and Th17 cells/Tregs, and decreased neuroinflammation were additionally observed in the group treated with shOGA Tregs (Figure 7, B–G, and Supplemental Figure 21E). These findings indicate that the reduction of OGA in Tregs ameliorated EAE development.

To further explore the potential resistance of shOGA Tregs to necroptosis in EAE, we coadministered control Tregs (CD45.1^+^EGFP^–^) and OGA-KD Tregs (CD45.2^+^EGPF^–^) at a 1:1 ratio, along with primed Th1/Th17 EGFP^+^ cells into RAG2-KO mice as described before. The shNC/shOGA Treg ratio was also reduced in RAG2-KO mice that received primed Th1/Th17 cells, while remaining constant in control RAG2-KO mice (Figure 7H). Analysis of necroptotic markers, including pRIPK3 and pMLKL, in CD45.2^+^EGFP^–^ Tregs showed decreased levels compared with CD45.1^+^EGFP^–^ Tregs during EAE pathogenesis, and the pathological conversion of Tregs into Th17 cells was unaffected by OGA deficiency (Supplemental Figure 21, F and G). Overall, these findings indicate that targeting OGA prevented the loss and dysfunction of Tregs due to necroptosis in EAE.

To further validate these findings and address potential limitations of the lentiviral shRNA approach, we established an independent Treg-intrinsic gene-editing strategy using FOXP3-Cas9 mice. Purified Tregs were transduced with gRNAs targeting *Ripk3*, *Mlkl*, or *Oga*, and Western blot analysis confirmed efficient depletion of the corresponding target proteins. We then adoptively transferred these gene-edited Tregs together with primed Th1/Th17 cells into Rag2-KO recipient mice. Consistent with our shOGA results and genetic evidence from the necroptosis pathway, Treg-intrinsic targeting of *Ripk3*, *Mlkl*, or *Oga* significantly attenuated EAE disease severity (Supplemental Figures 22 and 23). Together, these orthogonal approaches demonstrated that limiting OGA activity or disrupting the RIPK3/MLKL necroptotic axis within Tregs preserved Treg stability and function, thereby protecting against EAE pathogenesis.

### Necroptosis-resistant Tregs alleviate the pathogenesis of SLE.

In addition to MS, the loss and dysfunction of Tregs also contribute to the pathogenesis of SLE (59). To explore whether necroptosis-resistant Tregs could demonstrate enhanced suppressive capacity against pathogenic autoreactivity in SLE, we adapted the bm12-inducible model as previously described (60). Necroptosis-resistant Tregs (MLKL-KO or shOGA) or their respective control counterparts (WT or shNC) were coadministered along with splenocytes from bm12 mice (Figure 8A). The frequencies of Tfh cells, germinal center (GC) B cells and plasma cells were modestly, but significantly, reduced in groups treated with WT or shNC Tregs compared with the group receiving only bm12 lymphocytes (Figure 8B, and Supplemental Figure 24). In contrast, MLKL-KO or shOGA Tregs had a significantly stronger suppressive effect on the expansion of autoreactive T and B cells and splenocytes (Figure 8C). Consistent with these findings, kidney damage was significantly reduced in mice treated with necroptosis-resistant Tregs, as evidenced by decreased immune cell infiltration and reduced severity of nephritis compared with the WT and shNC groups (Figure 8, D and E). These results collectively indicate that necroptosis-resistant Tregs provide a promising therapeutic strategy for the treatment of SLE.

## Discussion

Understanding the mechanisms that compromise Treg functional persistence within inflamed tissues remains a major barrier to effective Treg-based immunotherapies. Here, we performed an unbiased pooled shRNA screen to identify regulators of Treg functional persistence in inflammatory environments driven by Th1/Th17 cells and microglia, which recapitulate sites of active inflammation in the MS CNS. We identified RIPK3 and demonstrated that its associated necroptotic signaling pathway is, to our knowledge, a previously unrecognized, death-driven checkpoint that limits Treg functional persistence. Independent validation and functional assays further showed that preventing necroptosis in Tregs was an effective strategy to enhance Treg-based immunotherapies against autoimmune pathology.

It is worth noting that most diseases targeted by current Treg therapy involve the upregulation of inflammatory ligands that could trigger Treg necroptosis. Given that both genetic and clinical evidence supports the safety of inhibiting necroptotic signaling, the necroptosis-resistant Tregs proposed in this study hold strong potential to complement existing strategies, such as reinforcement of FOXP3 expression (8–11), engineering of CAR-Tregs to operate in inflammatory environments (6, 7, 61), or induction of functional Tregs from Tcons (62, 63), through an independent, death-driven mechanism to enhance Treg-based immunotherapies.

In addition to their well-established role in autoimmune diseases, recent studies have highlighted the importance of Tregs in inflammatory disorders and neurological conditions. Autologous, ex vivo–expanded Treg transfer has shown promising clinical efficacy in a patient with refractory ulcerative colitis (64). Meanwhile, in rodent models of Parkinson’s disease, cotransplantation of autologous Tregs suppresses neuroinflammation and improves the survival of grafted dopamine neurons (65). Whether adoptive transfer of necroptosis-resistant Tregs could further enhance these therapeutic outcomes remains to be investigated.

Compared with Tcons, Tregs rely less on glucose to maintain their stability and function, as their master transcription factor FOXP3 suppresses glycolytic metabolism by inhibiting the expression of GLUT1 and MYC (14–16). This uniquely low-glucose metabolic profile enables Tregs to sustain their suppressive activity in glucose-poor tissues such as the ischemic brain and solid tumors (21, 66). Our findings demonstrate that this reduced glycolytic program led to a substantial decline in O-GlcNAc levels on RIPK1, thereby increasing the vulnerability of Tregs to necroptosis. Notably, quiescent naive T cells also display low glycolytic activity. However, they express relatively low levels of necroptotic pathway components, including MLKL, and are therefore resistant to necroptosis under conditions that can induce this form of cell death in Tregs (67). Additionally, Tregs can undergo metabolic adaptation when proliferation is required. During active proliferation, Tregs exhibit a strongly glycolytic phenotype with increased GLUT1 expression (68), but this occurs at the expense of diminished suppressive capacity and reduced FOXP3 expression (14) and probably makes them more resistant to necroptosis. This dynamic interplay between glucose metabolism and FOXP3 expression may function as an intrinsic feedback system that balances Treg stability and function, thereby preventing an overly immunosuppressive environment.

A similar feedback logic may also help reconcile the apparently opposing effects of TNF signaling on Tregs during inflammation. Tregs express high levels of TNFR2. TNFR2 signaling has been shown to promote Treg activation, proliferation, phenotypic stability, and suppressive function (69). Unlike TNFR1, which is efficiently activated by soluble TNF and can engage RIPK1-dependent death signaling, TNFR2 is preferentially activated by membrane-bound or multimeric TNF and does not contain a death domain (70, 71). Thus, TNFR2 signaling may initially support Treg expansion and function where cell-cell contact and membrane-bound TNF are abundant. However, exposure to soluble TNF at inflammatory sites may favor TNFR1/RIPK1-dependent necroptotic attrition, particularly once Tregs enter a metabolically vulnerable state with reduced RIPK1 O-GlcNAcylation. In addition, sustained TNFR2 engagement may lower the threshold for TNFR1/RIPK1-dependent death signaling, potentially through regulation of TRAF2 and cIAP availability (72, 73). Such crosstalk between TNFR2-mediated activation and TNFR1-mediated necroptotic attrition may represent a complementary negative feedback mechanism that limits excessive Treg accumulation and helps maintain immune balance.

Besides the dynamic regulation of proliferative capacity and necroptotic vulnerability, Tregs may undergo either proliferation or necroptosis dependent on the microenvironment during MS pathogenesis. DC-mediated antigen presentation provides the necessary T cell receptor (TCR) stimulation to drive Treg expansion, effectively creating a “proliferative niche” (74–76). Once Tregs exit this niche, they migrate to inflammatory sites, where they suppress immune responses by releasing antiinflammatory cytokines like IL-10. However, at these inflammatory sites, Tregs become vulnerable to necroptosis. Indeed, we observed that a substantial fraction of Tregs in the MS CNS exhibited markers of active necroptotic signaling, whereas necroptotic activation was detected only in a minor fraction of circulating Tregs (Figure 3). These observations not only support the idea that the highly inflammatory milieu of the CNS drives Treg necroptosis, but also raise the possibility that some Tregs initiating necroptosis within the CNS might reenter the circulation, a phenomenon that requires further investigation.

The dichotomy between proliferative expansion in permissive niches and necroptotic attrition at inflammatory sites could help explain the paradoxical increase in Treg counts observed in the cerebrospinal fluid of patients with MS (77). Additionally, in line with this dichotomy hypothesis, a recent study reported an enrichment of Tregs in the meninges and the third ventricle of mouse brains during EAE pathogenesis, whereas limited numbers of Tregs were detected within the brain parenchyma (78). These findings suggest that CNS border regions may serve as permissive niches for Treg accumulation or proliferation, while entry into the inflamed parenchyma may expose Tregs to signals that favor necroptotic attrition. Further studies are warranted to elucidate how Tregs proliferate or are protected from necroptosis at CNS border sites, and how their proliferative versus necroptotic fate is dynamically regulated as these cells traffic into the parenchyma.

Downregulation or loss of FOXP3 has long been considered a key mechanism driving Treg instability, particularly in inflammatory settings dominated by IL-6 signaling (79). In a murine model of inflammatory arthritis, synovial fibroblast–derived IL-6 promotes the conversion of Tregs into IL-17–producing “ex-Tregs,” thereby amplifying joint inflammation (42). In contrast to this transcriptional model of instability, our data reveal a mechanistically distinct pathway in which Th1/Th17-triggered microglial inflammatory responses predominantly activate necroptotic signaling in Tregs. Interestingly, our findings are consistent with in vivo Treg-tracking analyses showing that FOXP3 expression remains stable during autoimmune inflammation (80). These results raise the possibility that FOXP3 downregulation and necroptotic activation represent 2 context-dependent stress responses, each of which may compromise Treg functional persistence across autoimmune disease settings.

Loss-of-function mutations that promote necroptosis are associated with various autoimmune diseases (81–83). In light of the implications of these findings indicating that RIPK1 kinase-dependent necroptosis could be a key factor in the development of autoimmune diseases, clinical trials for RIPK1 inhibitors are currently underway for the treatment of these conditions. Previous studies have demonstrated that global inhibition of necroptosis is effective in ameliorating MS and SLE progression (84–87), but the involvement of Tregs in this process is unclear. In the present study, we applied multiple single-cell–based strategies to quantify necroptotic signaling activation, revealing a preferential vulnerability of Tregs to necroptosis as autoimmune disease progresses.

Given that autoimmune diseases primarily originate from a dysregulated immune system, the necroptotic vulnerability of Tregs may represent both a critical mechanism in disease development and a promising therapeutic target, particularly in light of the demonstrated feasibility of adoptive Treg transfer in recent clinical trials. In line with our findings, a previous study showed that Tregs show heightened sensitivity to necroptosis compared with Tcons when Casp8 is inhibited (88). However, the mechanisms underlying this selective vulnerability, as well as the therapeutic potential of preventing Treg necroptosis, remained unclear. Here, our findings demonstrate that a FOXP3-driven low-glucose metabolic program rendered Tregs intrinsically susceptible to necroptosis and further suggest that selective inhibition of necroptosis in Tregs, when combined with adoptive Treg transfer, can confer therapeutic benefit while avoiding the broad immunosuppression associated with global inhibition of inflammatory cell death pathways.

While our data indicate that increasing O-GlcNAc at RIPK1 (S332) was sufficient to eliminate the preferential vulnerability of Tregs to RIPK1 activation following TNF treatment, we cannot exclude the possibility that the relatively moderate decrease in O-GlcNAc on RIPK3 might also contribute to Treg necroptosis under certain conditions. Notably, O-GlcNAc modifications on both RIPK1 and RIPK3 have been shown to prevent the formation of the RIPK1-RIPK3 necrosome (55, 56). Our results suggest that pharmaceutical genetic enhancement of O-GlcNAc in Tregs could be a promising approach to prevent Treg necroptosis in autoimmune diseases and to successfully slow disease progression. Given that inhibitors of OGA have been developed for the treatment of Alzheimer’s diseases and are generally well tolerated (89), our findings highlight the importance of assessing the clinical effectiveness of these inhibitors for autoimmune diseases.

The execution of necroptosis requires the activation of MLKL by phosphorylation, which leads to the formation of amyloid-like polymers and eventual plasma membrane rupture (90). During necroptosis, there are also observations of lysosomal membrane permeabilization, mitochondrial fragmentation, and leakage of the nuclear membrane (91, 92). Indeed, before translocating into the plasma membrane, activated MLKL disrupts the integrity of the lysosome, leading to the release of luminal contents (91). It is noteworthy that homeostasis of the lysosomal nutrient–sensing complex metabolically controls Tregs stability and sustains its suppressive function (93, 94). Thus, pMLKL could potentially impair Treg function by targeting the lysosome before triggering cell death, a mechanism that requires further investigation.

A limitation of the translational component of this study is the relatively small size of our MS CyTOF cohort and the lack of an independent validation cohort. Furthermore, because our clinical analysis was restricted to peripheral blood, we lacked CNS-derived samples, such as cerebrospinal fluid or brain tissue, from these patients and therefore could not directly assess Treg necroptosis within the CNS compartment. Although our mouse models provided robust mechanistic insights into CNS pathology, future clinical studies using larger cohorts and CNS sampling will be required to further validate our findings in humans.

Overall, our study identifies necroptosis as a previously unrecognized, death-driven checkpoint that compromises Treg functional persistence at sites of active inflammation and underscores the therapeutic potential of adoptive transfer of necroptosis-resistant Tregs.

## Methods

Further information can be found in Supplemental Methods.

### Sex as a biological variable.

For human blood–based studies, both male and female individuals were included. For mouse studies, only male mice were used, including mice used for EAE and SLE models. Male mice were selected to maintain consistency across experimental cohorts and to minimize potential variability associated with sex-dependent differences in disease susceptibility and immune responses. Because sex-dependent differences have been reported in both EAE and SLE models, whether the findings presented here are equally applicable to female mice remains to be determined and will require further validation.

### Statistics.

Data are presented as the mean ± SEM. Comparisons between 2 groups were performed using an unpaired, 2-tailed Student’s *t* test. For comparisons involving more than 2 groups with a single independent variable, 1-way ANOVA was performed. For experiments involving 2 independent variables, 2-way ANOVA was performed, including assessment of the interaction between the 2 factors. Post hoc multiple-comparison tests were selected on the basis of the prespecified comparisons: Dunnett’s multiple-comparison test was used when multiple experimental groups were compared with a single, predefined control group; Tukey’s multiple-comparison test was used for all pairwise comparisons among groups; and Šidák’s multiple-comparison test was used for prespecified pairwise comparisons. A *P* value of less than 0.05 was considered statistically significant. The number of samples per group (*n*) or the number of experiments is specified in the figure legends.

### Study approval.

All animal experimental protocols were approved by the ethics committees of the Interdisciplinary Research Center on Biology and Chemistry (IRCBC), Chinese Academy of Sciences. The clinical study was approved by the IRB of Renji Hospital of Shanghai Jiaotong University and was performed in accordance with the Declaration of Helsinki. Written informed consent was obtained from all patients or their legally authorized representatives.

### Data availability.

Data are available in the Supporting Data Values file and the associated supplemental materials. Sequencing data have been deposited in the NCBI’s Sequence Read Archive (SRA) under accession number PRJNA1484746 (www.ncbi.nlm.nih.gov/bioproject/?term=PRJNA1484746%201484746[uid]). Values for all data points in graphs are reported in the Supporting Data Values file.

## Manuscript information

### Author contributions.

CZ conceptualized the study. QW NC, LG, ZL, RP, XL, and HP designed the methodology. QW, NC, LG, ZL, XA, RP, XL, and HP designed the methodology and conducted the experiments. XA participated in experimental design and performed part of the experimental work for this study. QW and CZ wrote the original draft of the manuscript. LG, PL, DX, JY, and CZ reviewed and edited the manuscript. LG, JY, and CZ acquired funding for the study. LG, HP, PL, DX, JY, and CZ provided resources. LG, PL, DX, JY, and CZ supervised the study.

### AI disclosure.

ChatGPT-5 was used solely to check grammar and polish the language of the manuscript. AI tools were not used to generate the original manuscript content or to design or conduct the research.

### Conflict of interest.

The authors have declared that no conflict of interest exists.

### Funding.

The funding sources for this manuscript are listed below.

- National Natural Science Foundation of China grant 82188101 (to JY).
- Shanghai Municipal Science and Technology Major Project grant 22JC1410400 (to JY and CZ).
- Shanghai Municipal Science and Technology Major Project (2025) (to JY and CZ).
- Shanghai Basic Research Pioneer Project (to JY and CZ).
- Shanghai Key Laboratory of Aging Studies grant 19DZ2260400 (to JY and CZ).
- CAS Pioneer Initiative grant 2024000026 (to CZ).
- Shanghai Oriental Talent Program for Leading Professionals grant LJ2025063 (to CZ).
- National Special Plan for High-level Talents grant 2022000178 (to CZ).
- Young Talent Cultivation Program of CAS at Shanghai grant 2023000010 (to CZ).
- NSFC Promotion Program of Renji Hospital, Shanghai Jiao Tong University School of Medicine grant RJTJ26-MS-017 (to LG).
- National Natural Science Foundation of China grant 81801298 (to LG).

## Supplementary Material

Supplemental data

Supplemental data set 1

Unedited blot and gel images

Supporting data values

## Figures and Tables

**Figure 1:**
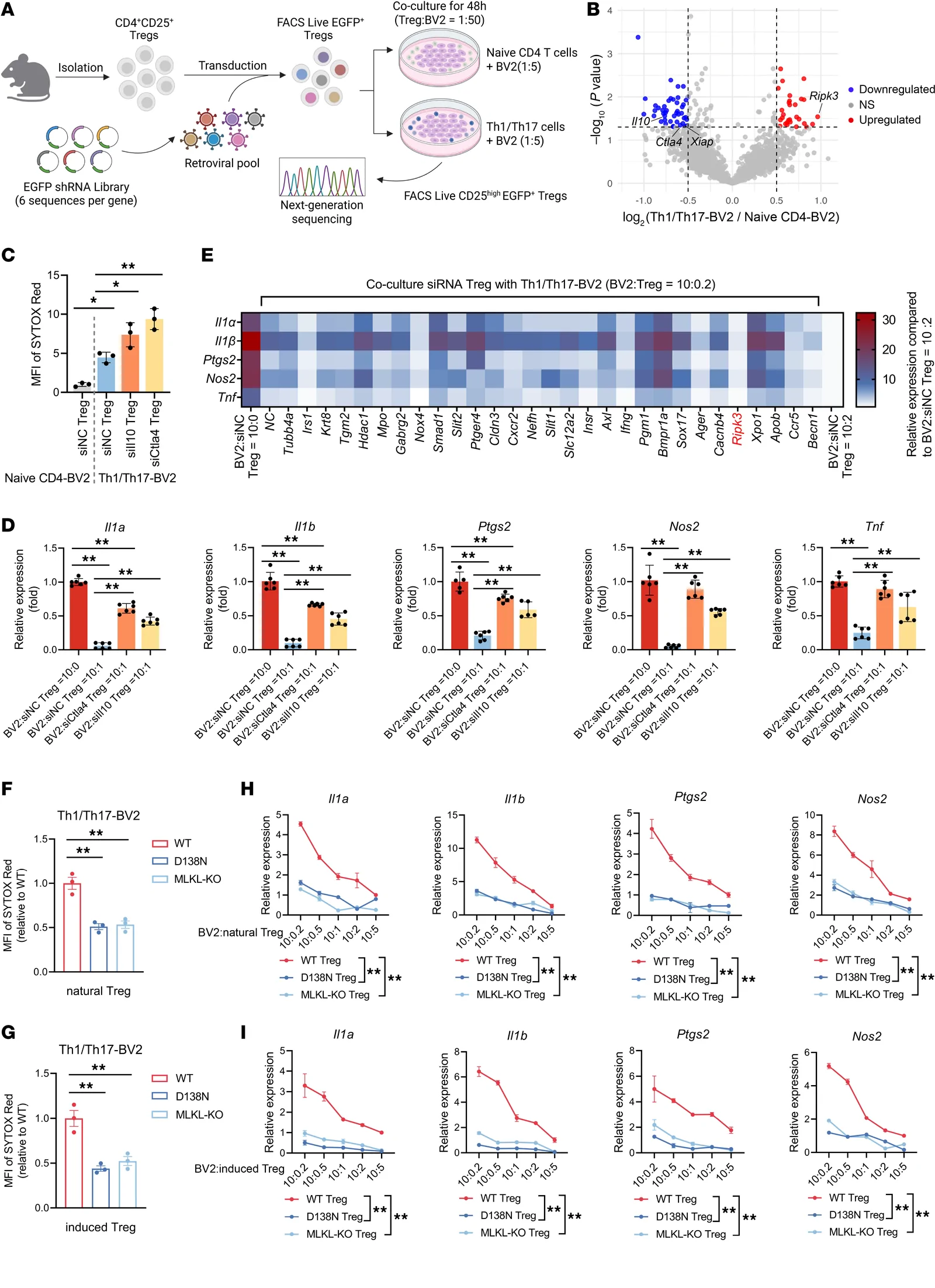
A functional genetic screen identifies necroptotic signaling as a primary driver of compromised Treg functional persistence at sites of active inflammation. (**A**) Schematic of the pooled shRNA screen. (**B**) Volcano plot of candidate genes identified by the screen. Positive and negative regulators are shown in red and blue, respectively. (**C**) Flow cytometric analysis of SYTOX Red staining as an indicator of cell death in Tregs. BV2 and Tregs were cocultured at a ratio of 10:0.2 (*n* = 3 independent experiments). (**D**) qPCR analysis of inflammatory cytokine expression in BV2 cells after coculture with the indicated Tregs (*n* = 5–6 independent experiments). (**E**) Heatmap showing mean inflammatory cytokine expression in BV2 cells from the Th1/Th17-BV2 coculture system. Tregs transduced with indicated siRNAs were added to the coculture system at a BV2/Treg ratio of 10:0.2. After 48 hours, inflammatory cytokine mRNA levels in BV2 were quantified by qPCR. BV2 cultured alone (10:0) and BV2 cocultured with siNC-transduced Tregs (10:2) served as positive and negative controls, respectively. Data are shown as the fold change relative to BV2/siNC Treg (10:2). (*n* = 3 independent experiments). (**F** and **G**) Flow cytometric analysis of SYTOX Red staining in natural and induced Tregs within the Th1/Th17-BV2 coculture system. BV2 and Tregs were cocultured at a ratio of 10:0.2 (*n* = 3 independent experiments). (**H** and **I**) Expression of inflammatory cytokines in BV2 cells from the Th1/Th17-BV2 coculture system. Cytokine expression was quantified by qPCR after 48 hours of coculture with natural or induced Tregs at different BV2/Treg ratios (*n* = 3 independent experiments). Data indicate the mean ± SEM. *P < 0.05 and **P < 0.01, by 1-way ANOVA with Dunnett’s multiple-comparison test (**C**, **F**, and **G**), 1-way ANOVA with Šidák’s multiple-comparison test (**D**), or 2-way ANOVA with Dunnett’s multiple-comparison test (**H** and **I**).

**Figure 2:**
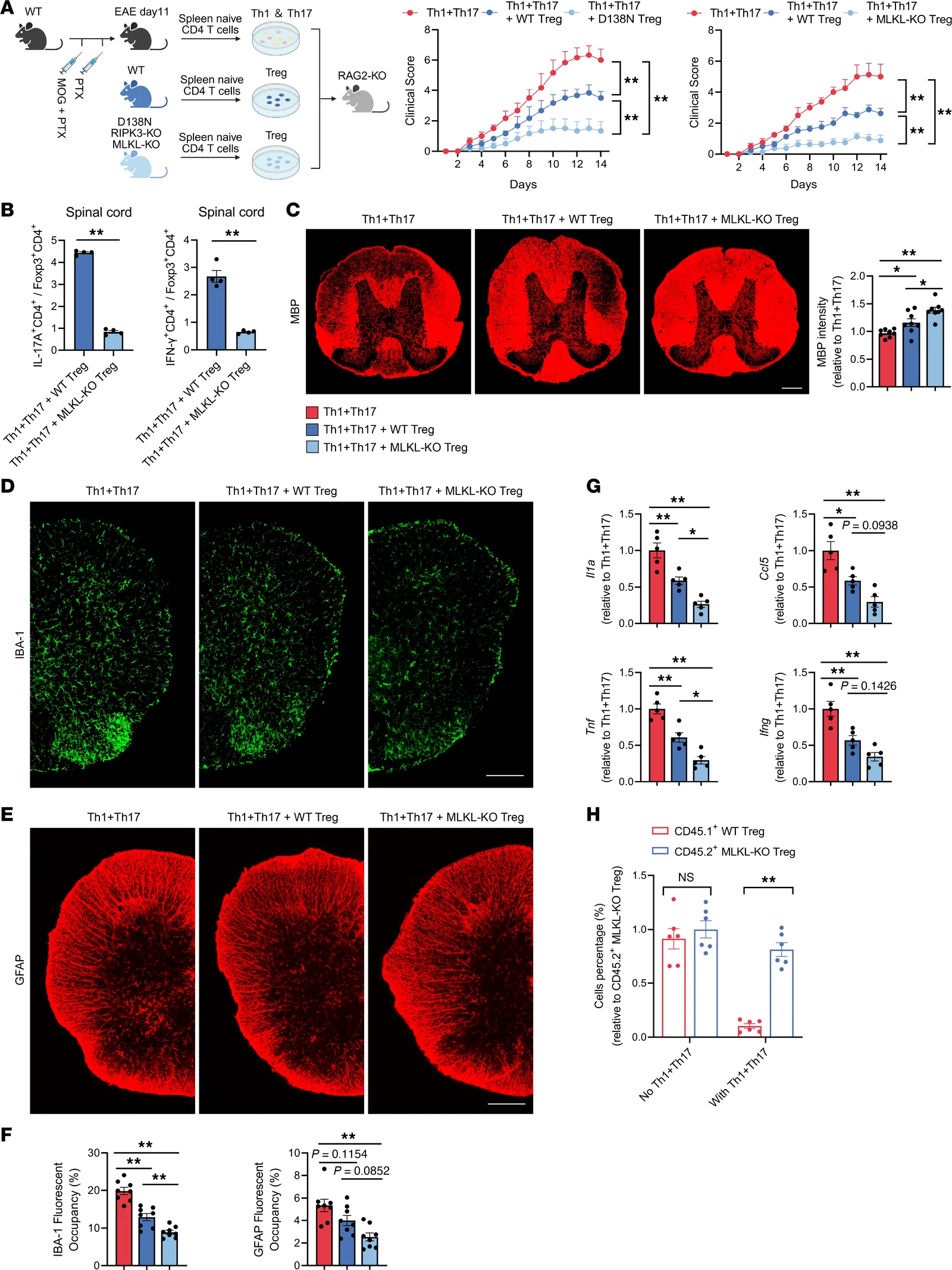
Preventing Tregs necroptosis ameliorates EAE pathogenesis. (**A**) Clinical scores for passive EAE mice that received in vitro–primed Th1/Th17 cells along with WT Tregs, RIPK1-D138N Tregs, or MLKL-KO Tregs (*n* = 6–8 mice per group). (**B**) Ratios of IFN-γ^+^CD4^+^ to Foxp3^+^CD4^+^ cells and IL-17A^+^CD4^+^ to Foxp3^+^CD4^+^ cells in the spinal cord of passive EAE mice on day14 after injection (*n* = 4 mice per group). (**C**) Immunofluorescence analysis of the demyelination process induced by passive EAE in the spinal cord of mice, 2 weeks after injection of in vitro–primed Th1/Th17 cells together with WT Tregs or MLKL-KO Tregs. The extent of myelination was quantified using the myelin marker MBP (scale bar: 200 μm; *n* = 8 mice per group). (**D**–**F**) Ionized calcium-binding adapter molecule (1 IBA-1) and glial fibrillary acidic protein (GFAP) staining was done to quantify microglial and astrocyte activation in the spinal cord of passive EAE mice on day14 after injection (scale bars: 200 μm; *n* = 8 mice per group). (**G**) Expression levels of inflammatory cytokines in the spinal cord of passive EAE mice, quantified by qPCR on day14 after transfer (*n* = 5 mice per group). (**H**) Percentage of CD45.1^+^ WT Tregs and CD45.2^+^ MLKL-KO Tregs in the blood of Rag2-KO mice, with or without cotransfer of primed Th1/Th17 cells. Mice were sacrificed 14 days after receiving an equal number of WT Tregs and MLKL-KO Tregs. Data were normalized to the number of CD45.2^+^ MLKL-KO Tregs in Rag2-KO mice (*n* = 6 mice per group). Data indicate the mean ± SEM. *P < 0.05 and **P < 0.01, by unpaired, 2-tailed Student’s *t* test (**B** and **H**), 1-way ANOVA with Tukey’s multiple-comparison test (**C**, **F** and **G**), or 2-way ANOVA with Tukey’s multiple-comparison test (**A**).

**Figure 3:**
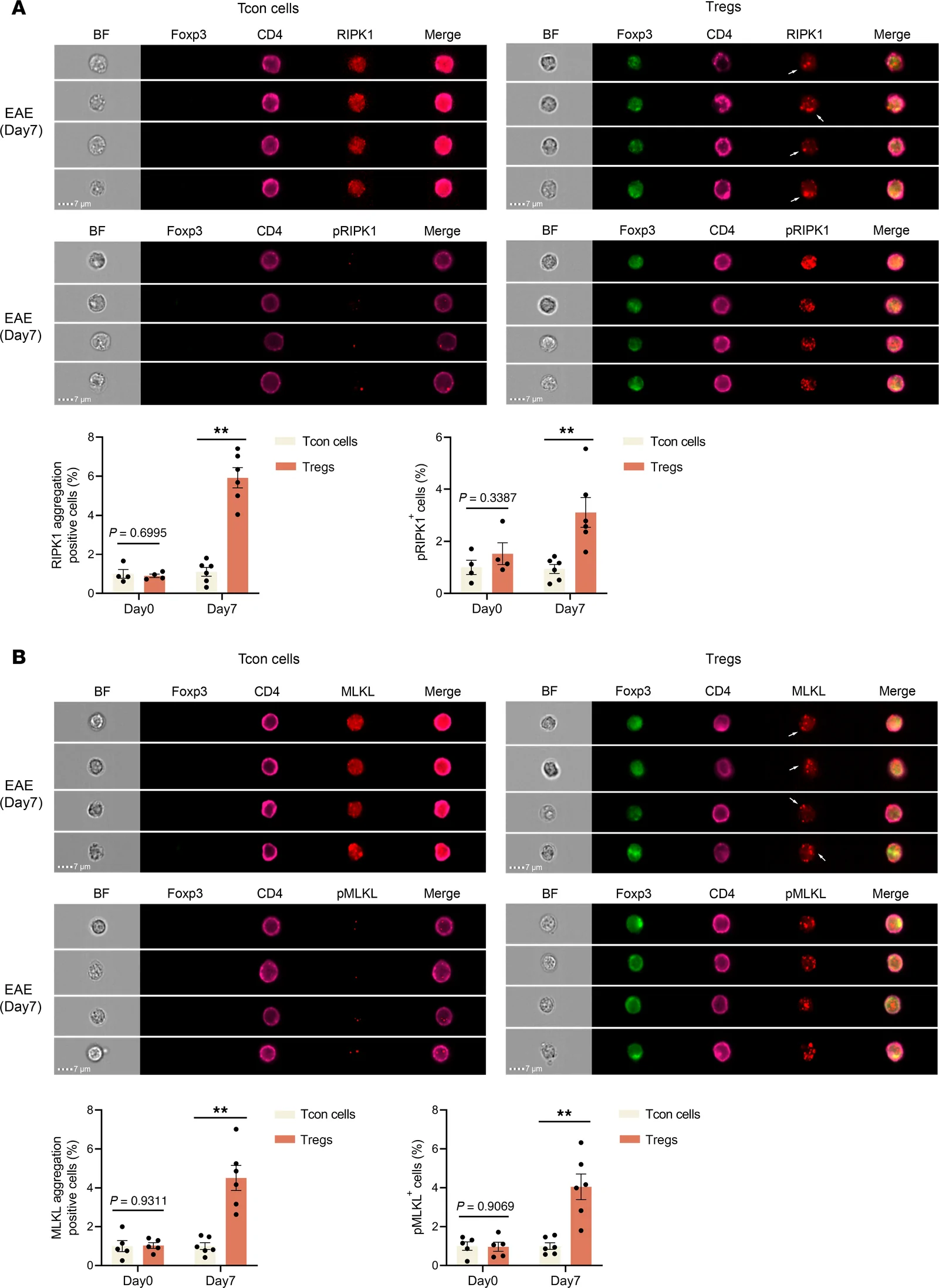
Tregs exhibit heightened susceptibility to necroptosis during EAE development. (**A** and **B**) Representative images, acquired using IFC, display RIPK1 and pRIPK1 staining as well as MLKL and pMLKL staining in circulating Tcons (CD4^+^FOXP3^–^) and Tregs (CD4^+^FOXP3^+^) from EAE mice on day 7 after immunization. The statistical graph shows the percentage of cells exhibiting MLKL aggregation (indicated in the images by white arrows) or pMLKL staining in Tcons and Tregs at day 0 and day 7 of EAE. Data were normalized to Tcons on day 0 (scale bars: 7 μm; *n* = 4–6 mice per group). Data indicate the mean ± SEM. **P < 0.01, by unpaired, 2-tailed Student’s *t* test (**A** and **B**). BF, bright field.

**Figure 4:**
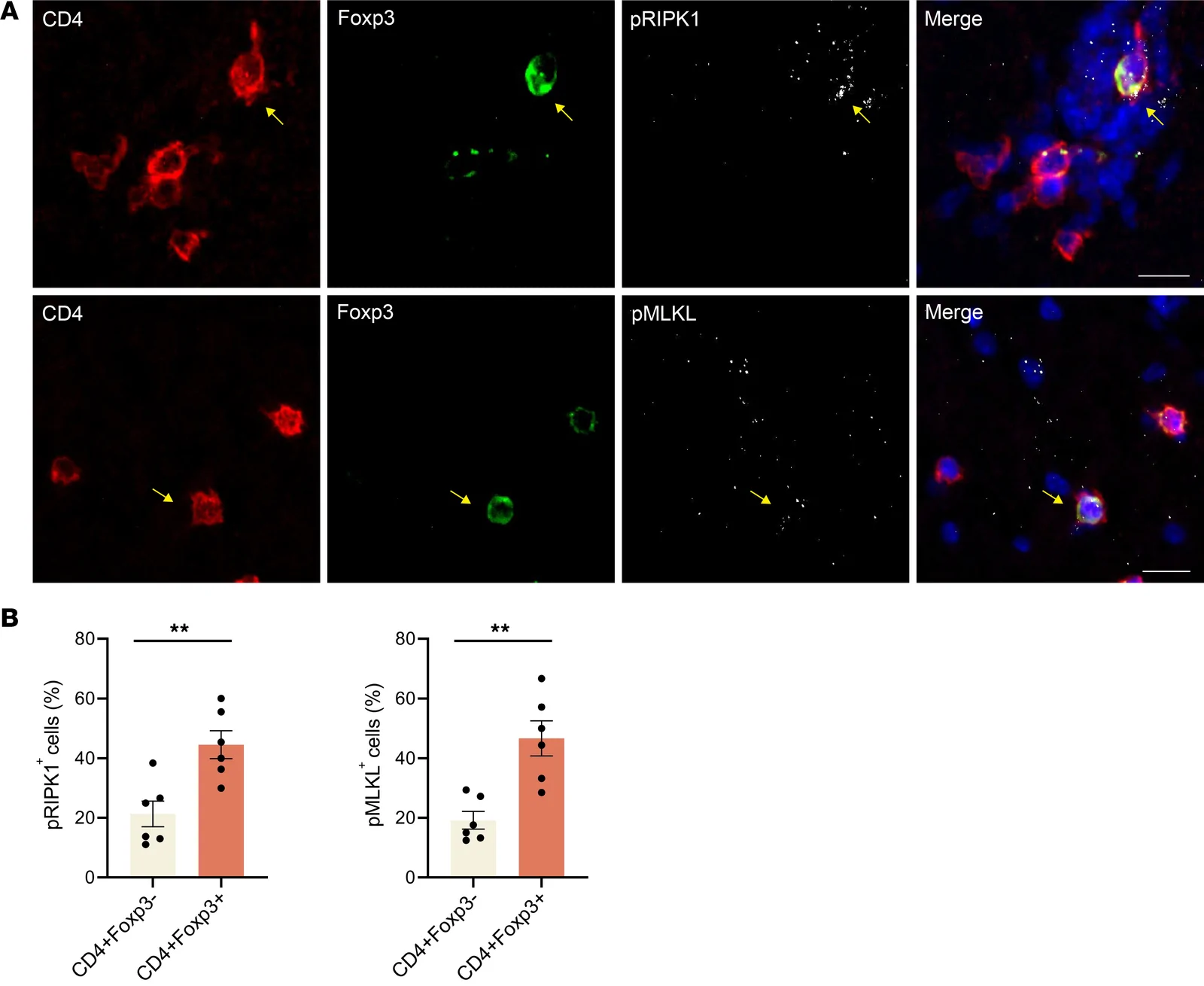
Tregs exhibit enhanced necroptotic signaling in the spinal cord of EAE mice. (**A**) Immunofluorescence analysis of pRIPK1 and pMLKL expression in Treg and Tcons in the spinal cord of EAE mice on day 21 after EAE induction (scale bars: 10 μm). (**B**) Quantification of the proportion of pRIPK1^+^/pMLKL^+^ cells among Treg and Tcons in spinal cord sections (*n* = 6 mice per group). Data indicate the mean ± SEM. **P < 0.01, by unpaired, 2-tailed Student’s *t* test (**B**).

**Figure 5:**
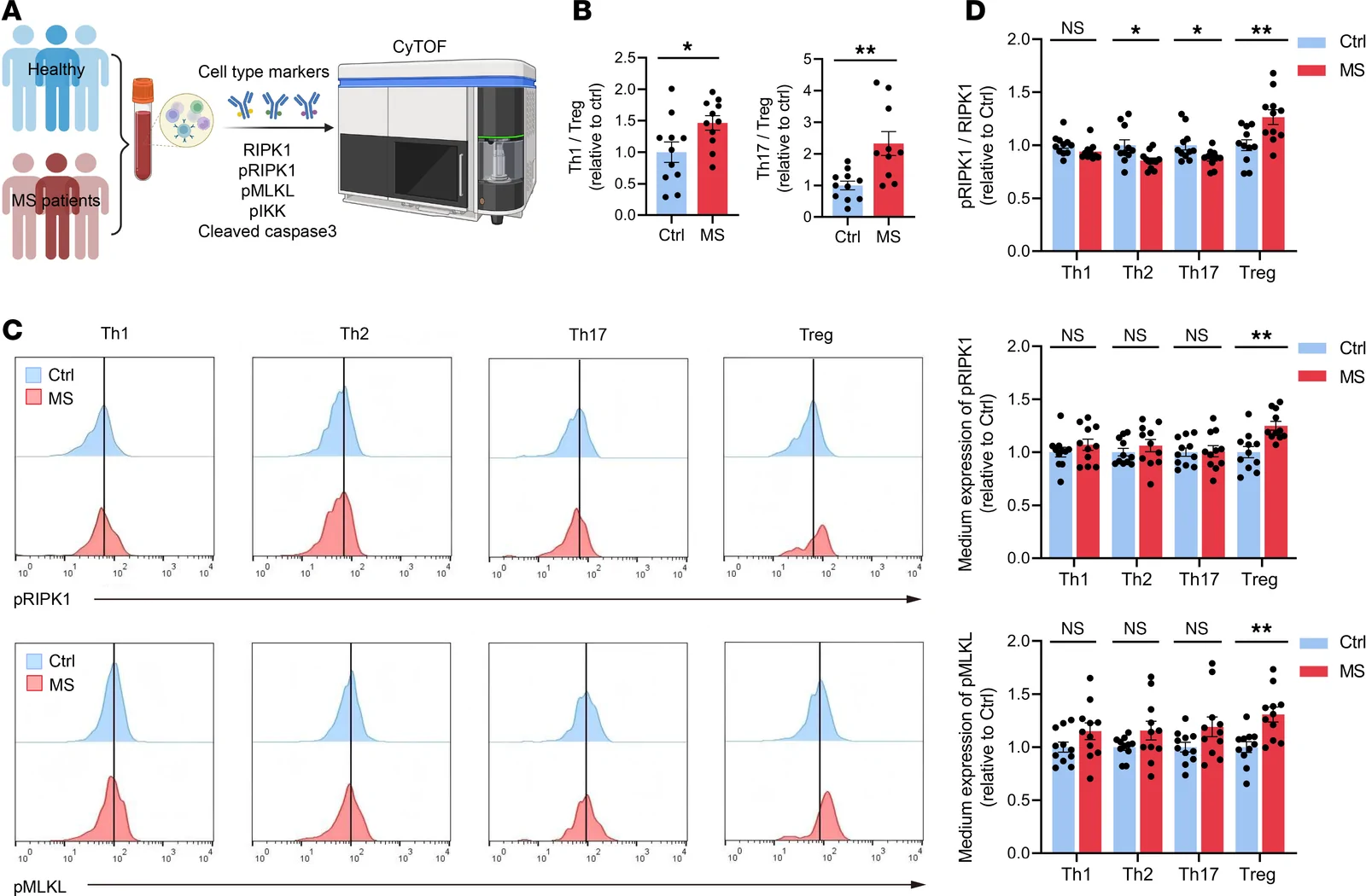
Tregs are preferentially susceptible to necroptosis in patients with MS. (**A**) The diagram illustrates the CyTOF process. (**B**) The ratios of Th1 to Tregs and Th17 to Tregs in patients with MS and healthy controls (*n* = 11 patients with MS; *n* = 11 healthy control volunteers). (**C**) The medium expression levels of pRIPK1 and pMLKL in Th1, Th2, Th17, and Tregs from patients with MS and healthy controls (*n* = 11 patients with MS; *n* = 11 healthy controls). (**D**) The ratios of pRIPK1 to RIPK1 in Th1, Th2, Th17, and Tregs (*n* = 11 patients with MS; *n* = 11 healthy controls). Data indicate the mean ± SEM. *P < 0.05 and **P < 0.01, by unpaired, 2-tailed Student’s *t* test (**B**–**D**).

**Figure 6:**
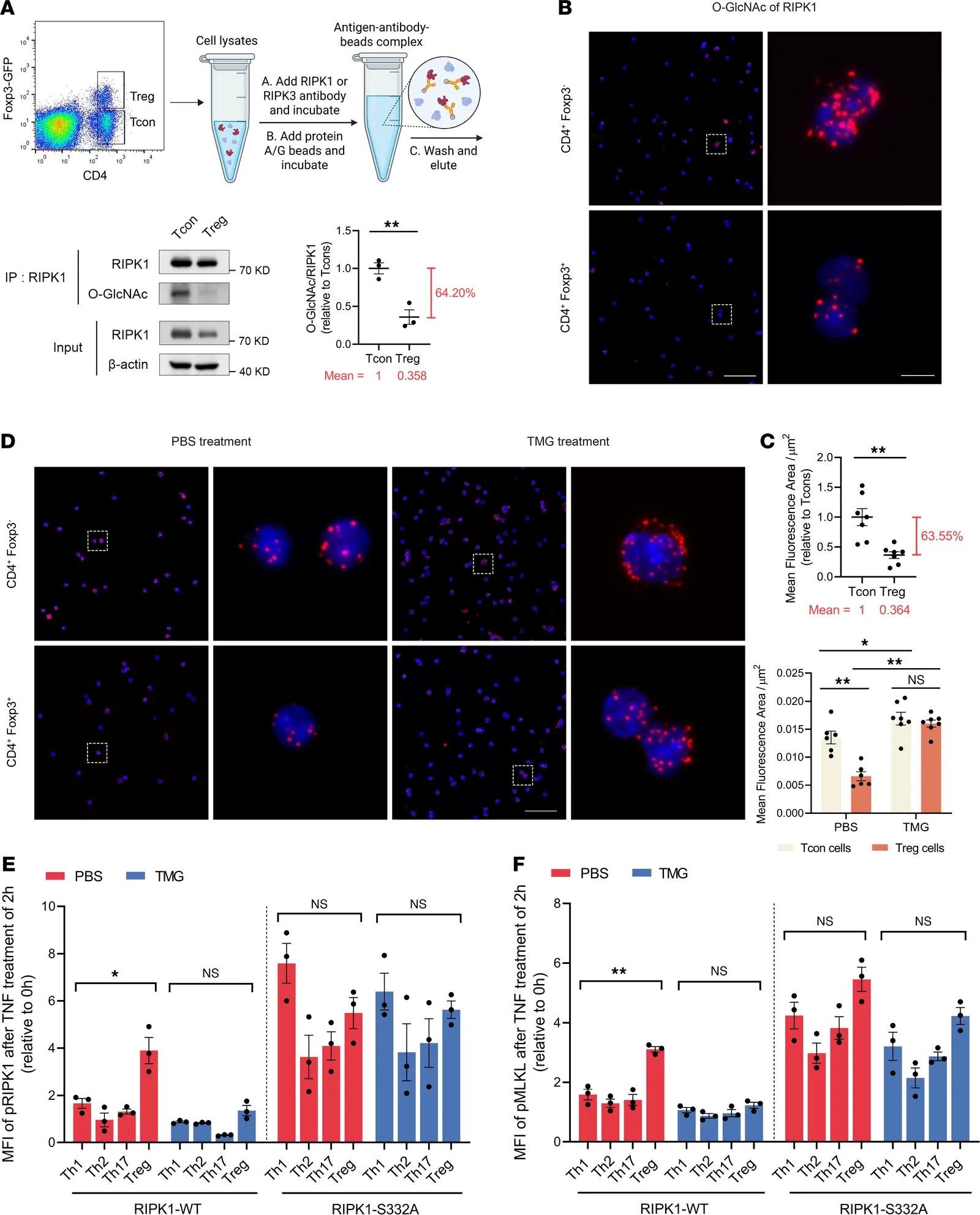
Reduced O-GlcNAc modification of RIPK1 underlies necroptotic vulnerability in Tregs. (**A**) Detection of O-GlcNAc modification on RIPK1 using IP assay in isolated Tcons (CD4^+^FOXP3^–^) and Treg (CD4^+^FOXP3^+^) cells (*n* = 3 independent experiments per group). (**B** and **C**) Detection of O-GlcNAc modification on RIPK1 by PLA in Tcons and Tregs (scale bars: 50 μm [left]; 5 μm [right]; *n* = 7 independent experiments per group). (**D**) Detection of O-GlcNAc modification on RIPK1 using PLA in Tcons and Tregs isolated from mice pretreated with 20 mg/kg TMG (scale bars: 50 μm [left]; 5 μm [right]; *n* = 6–7 independent experiments per group). (**E** and **F**) The expression of pRIPK1 and pMLKL in Th1, Th2, Th17, and Tregs from RIPK1-WT and RIPK1-S332A mice pretreated with either PBS or 20 mg/kg TMG, 2 hours after i.v. injection of 4 μg TNF (*n* = 3 mice per group). Data indicate the mean ± SEM. *P < 0.05 and **P < 0.01, by unpaired, 2-tailed Student’s *t* test (**A** and **C**), 2-way ANOVA with Šidák’s multiple-comparison test (**D**), or 1-way ANOVA with Dunnett’s multiple-comparison test (**E** and **F**).

**Figure 7:**
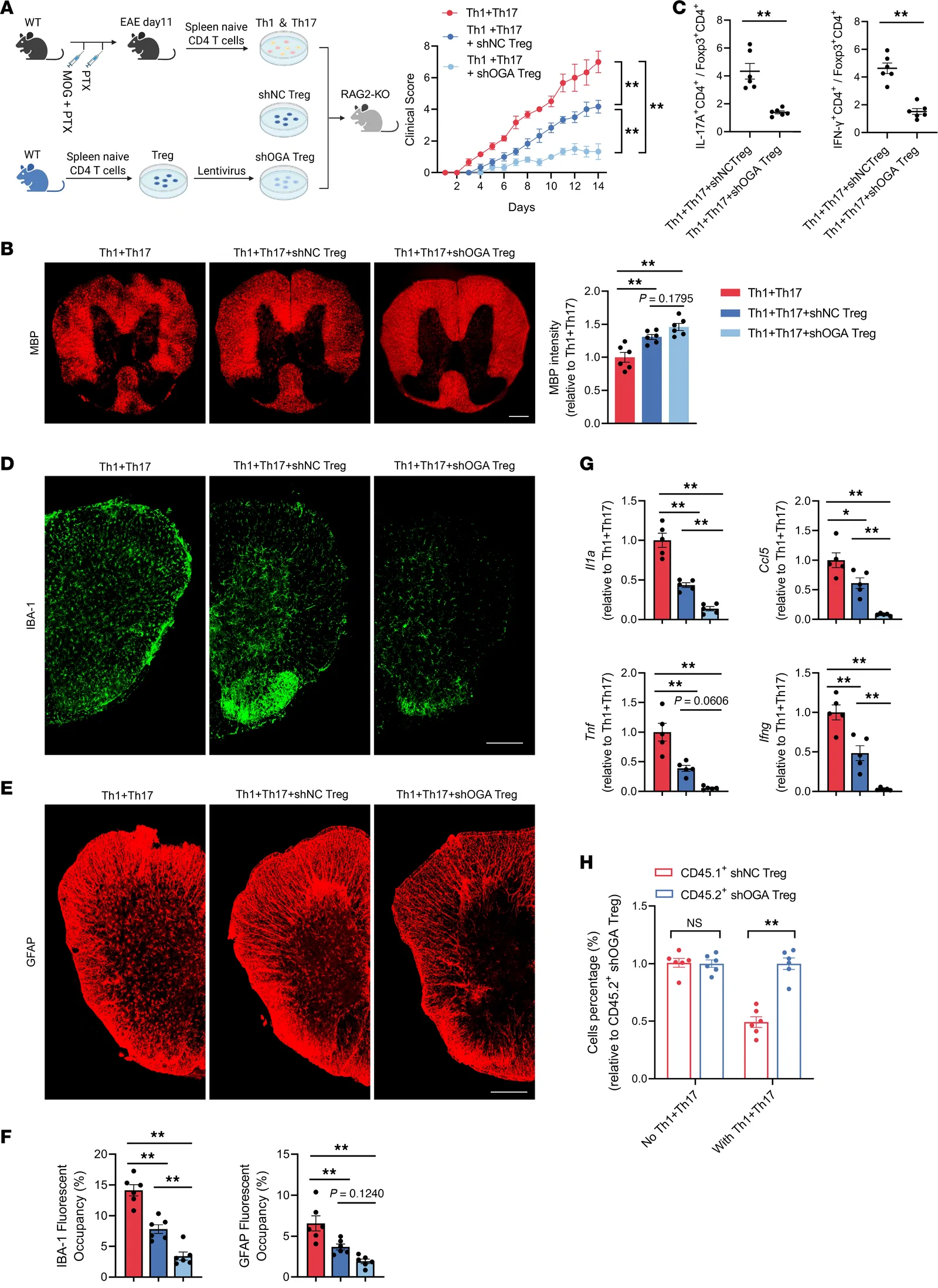
OGA reduction in Tregs mitigates EAE pathogenesis. (**A**) Clinical scores for passive EAE mice that received in vitro primed–Th1/Th17 cells along with shOGA Tregs or shNC Tregs (*n* = 6 mice per group). (**B**) Immunofluorescence analysis of the demyelination process induced by passive EAE in the spinal cord of mice, 2 weeks after injection of in vitro–primed Th1/Th17 cells together with shNC Tregs or shOGA Tregs. The extent of myelination was quantified using the myelin marker MBP (scale bars: 200 μm, *n* = 6 mice per group). (**C**) The ratios of IFN-γ^+^CD4^+^ to Foxp3^+^CD4^+^ cells and IL-17A^+^CD4^+^ to Foxp3^+^CD4^+^ cells in the spinal cord of passive EAE mice on day14 after injection (*n* = 6 mice per group). (**D**–**F**) IBA-1 and GFAP staining was done to quantify microglial and astrocyte activation in the spinal cord of passive EAE mice on day14 after injection (scale bars: 200 μm, *n* = 6 mice per group). (**G**) Expression of inflammatory cytokines in the spinal cord of passive EAE mice, quantified by qPCR on day14 after injection (*n* = 5 mice per group). (**H**) The percentage of CD45.1^+^ shNC Tregs and CD45.2^+^ shOGA Tregs in the blood of Rag2-KO mice, with or without cotransfer of primed Th1/Th17 cells. Mice were sacrificed 14 days after receiving an equal number of shNC Tregs and shOGA Tregs. Data were normalized to CD45.2^+^ shOGA Tregs (*n* = 6 mice per group). Data indicate the mean ± SEM. *P < 0.05 and **P < 0.01, by unpaired, 2-tailed Student’s *t* test (**C** and **H**), 1-way ANOVA with Tukey’s multiple-comparison test (**B**, **F**, and **G**), or 2-way ANOVA with Tukey’s multiple-comparison test (**A**).

**Figure 8:**
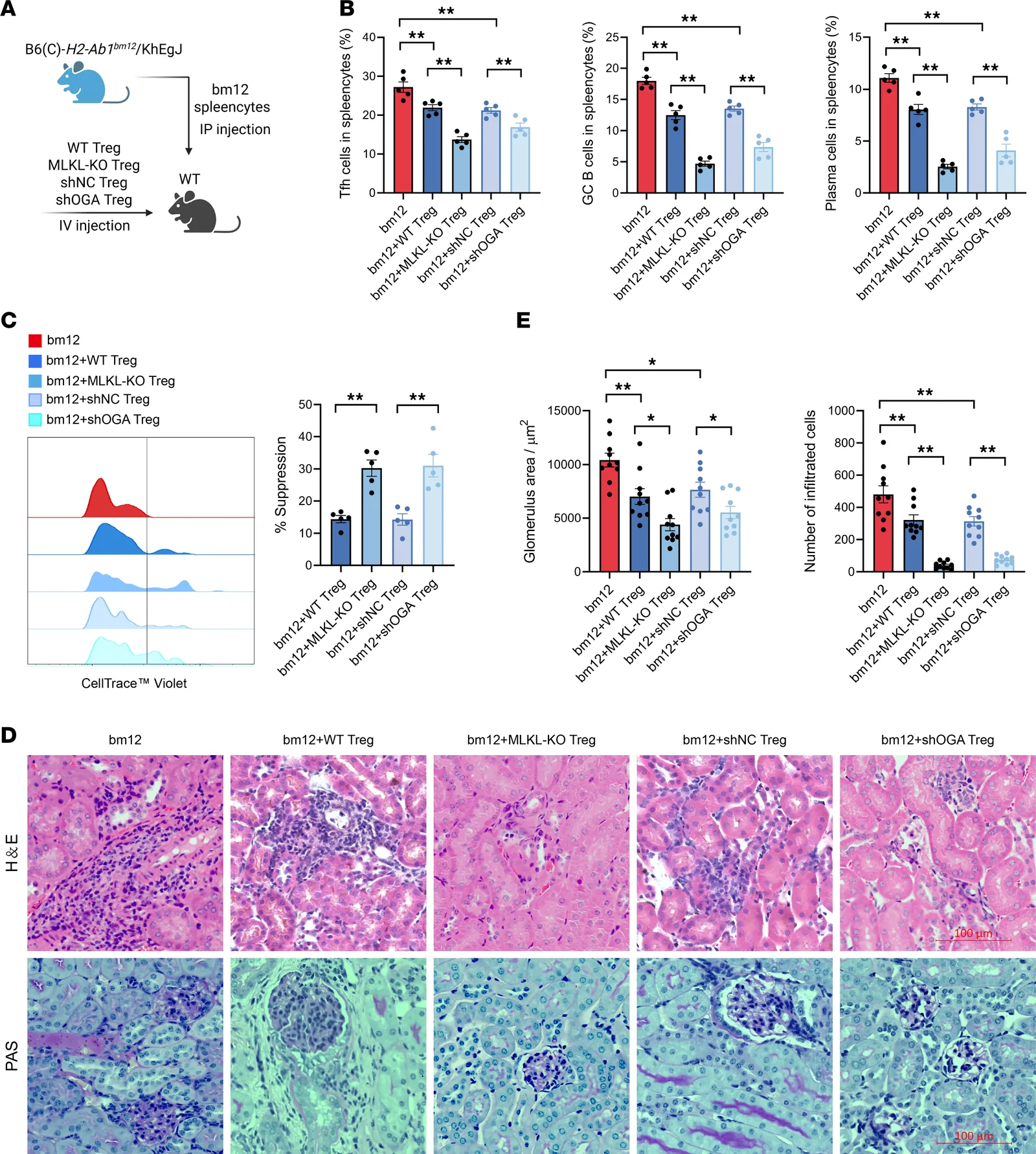
Necroptosis-resistant Tregs attenuate SLE pathogenesis. (**A**) Diagram illustrates the procedure for constructing the bm12 SLE model. WT mice received 1 × 10^6^ WT Tregs, MLKL-KO Tregs, shNC Tregs, or shOGA Tregs via tail vein injection, following i.p. injection with 30 million splenocytes isolated from bm12 mice. (**B**) Flow cytometric analysis of the percentage of Tfh cells, plasma cells, and GC B cells in the spleens of SLE mice on day 14 after injection of bm12 splenocytes (*n* = 5 mice per group). (**C**) Proliferation of CellTrace Violet–labeled (CTV-labeled) autoreactive bm12 splenocytes in the spleen of SLE mice on day 14 after injection. The statistical graph shows the percentage of suppressed proliferation bm12 lymphocytes in SLE mice received WT Tregs, MLKL-KO Tregs, shNC Tregs, and shOGA Tregs, as determined by CTV intensity (*n* = 5 mice per group). (**D** and **E**) Pathology of kidney tissue sections from SLE mice stained with H&E and periodic acid–Schiff (PAS) on day 30 after injection (scale bars: 100 μm). The statistical graph shows the number of infiltrated immune cells in renal tissue (assessed by H&E staining) and the glomerulus area in SLE mice (evaluated by PAS staining) in kidney sections. (*n* = 10 mice per group). Data indicate the mean ± SEM. *P < 0.05 and **P < 0.01, by 1-way ANOVA with Šidák’s multiple-comparison test (**B**, **C**, and **E**).

## Version 1 09/01/2026

In-Press Preview

## Version 2 11/02/2026

Electronic publication
