# Supplementary material for: Enhancing Treg persistence by inhibiting necroptosis restrains autoimmune pathology
Source: J Clin Invest. 2026 Sep 1;136(21):e207077. doi: 10.1172/JCI207077 (PMC13722395; doi:10.1172/JCI207077)
Supplement: Supplementary file 1 [file jci-136-207077-s211.pdf]

Supplemental Information

Enhancing Treg persistence by inhibiting necroptosis restrains autoimmune pathology

Qiaoyan Wu[1,2], Na Cui[1], Li Gao[3], Zhihui Lu[1,2], Xiang Ao[1,2], Rui Pang[4,5], Xingyan Li[1], Heling Pan[1], Daichao Xu[1,6], Peiying Li[4,5], Junying Yuan[1,6] and Chengyu Zou[1,6]

[1]Interdisciplinary Research Center on Biology and Chemistry, Shanghai Institute of Organic Chemistry, Chinese Academy of Sciences, Shanghai, China.

[2]University of Chinese Academy of Sciences, Beijing, China.

[3]Department of Neurology, Renji Hospital, Shanghai Jiao Tong University School of Medicine, Shanghai, China.

[4]Department of Anesthesiology, Key Laboratory of the Ministry of Education, Renji Hospital, Shanghai Jiao Tong University School of Medicine, Shanghai, China.

[5]Clinical Research Center, Renji Hospital, Shanghai Jiao Tong University School of Medicine, Shanghai, China.

[6]Shanghai Key Laboratory of Aging Studies, Shanghai, China.

Address correspondence to: Chengyu Zou (lead contact), Interdisciplinary Research Center on Biology and Chemistry, Shanghai Institute of Organic Chemistry, Chinese Academy of Sciences, Shanghai 200010, China. Phone: 86-21-66567086; Email: czou@mail.sioc.ac.cn. Or to Li Gao, Department of Neurology, Renji Hospital, Shanghai Jiao Tong University School of Medicine, Shanghai 200010, China. Phone: 86-21-68383501; Email: gaoli246619@126.com.

Supplemental Methods:

Natural $CD4^+CD25^+$ Treg isolation

For large-scale isolation of natural Treg cells, Treg expansion was first induced in mice of the indicated genotypes by daily intraperitoneal injection of 2.5 μg IL-2–Fc fusion protein for three consecutive days (1). On day 5, spleens were harvested and single-cell suspensions were prepared. Total $CD4^+$ T cells were enriched by negative selection using a Mouse $CD4^+$ T-Cell Isolation Kit (Selleck, Cat. #: B90001), followed by positive selection of $CD25^+$ cells using a EasySep™ Mouse CD25 Regulatory T Cell Positive Selection Kit (STEMCELL, Cat. #: 18782) to obtain $CD4^+CD25^+$ Treg cells.

The isolated Treg cells were cultured in X-VIVO15 medium (Lonza, Cat. #: 04-418Q) supplemented with 10% fetal bovine serum (FBS), 1% penicillin–streptomycin, 50 μM 2-mercaptoethanol, and 10 ng/mL IL-2 (PeproTech, Cat. #: 210-12). For Treg expansion, cells were additionally stimulated with CD3/CD28 T Cell Activator (STEMCELL, Cat. #: 100-1572).

Isolation and culture of primary microglia

Primary microglia were isolated from neonatal WT mouse brains. Briefly, forebrains from 1~2-day-old mouse pups were dissected, enzymatically digested with 0.01% trypsin, and gently triturated in DMEM supplemented with 10% heat-inactivated fetal bovine serum (FBS) and 1% penicillin–streptomycin. The resulting cell suspension was plated into poly-D-lysine (Sangon Biotech, Cat. #: E607015-0005) coated 75 cm² flasks and maintained at 37°C in a

humidified incubator with 5% CO_2, with medium changes every 3 days for 7–10 days (2).

After an initial 1-hour orbital shaking to detach microglia, floating cells were collected and replated in fresh medium at a density of 2 × 10^5 cells/mL in DMEM containing 10% FBS and 1% penicillin–streptomycin, and cultured under standard conditions (37°C, 5% CO_2).

Purity of isolated microglia was assessed by flow cytometry using CD11b (Biolegend, Cat. #: 101206, Clone: M1/70) as a microglial marker, with >95% of cells identified as $CD11b^+$. In addition, nearly all cells were IBA-1 (Abcam, Cat. #: ab178847, Clone: EPR16589) positive, further validating the high purity of the microglial population.

sgRNA Transfection of Treg Cells

$Foxp3^{tdTomato}$ × Cas9 mice were treated with IL-2–Fc fusion protein to expand Treg cells in vivo. Splenic $tdTomato^+$ Treg cells were isolated by fluorescence-activated cell sorting (FACS) and transfected with AF488-labeled sgRNAs (three sgRNAs per target gene, Supplemental Table 6) using the ProteanFect™ CRISPRMax Mouse Primary Immune Cell Transfection Kit (NANOPORTAL BIOTECH, Cat. #: PT06) according to the manufacturer's instructions. Seventy-two hours after transfection, $AF488^+tdTomato^+$ Treg cells were sorted by FACS and expanded in vitro for 5 days. Gene-editing efficiency was then assessed, and for each target gene, Treg cells transfected with the sgRNA exhibiting the

highest knockout efficiency were selected for adoptive transfer into RAG2-KO mice.

Bulk RNA Sequencing and Bioinformatic Analysis

BV2 cells cultured alone, co-cultured with naïve $CD4^+$ T cells, or co-cultured with Th1+Th17 cells were collected (5×10^6 cells per sample; n = 3 biological replicates per group). Total RNA was extracted using TRIzol Reagent and submitted to GENEWIZ for library preparation and bulk RNA sequencing. Raw sequencing reads were subjected to quality control using FastQC (v0.10.1). Clean reads were aligned to the mouse reference genome (mm10) using HISAT2 (v2.2.1). Gene expression was quantified as raw read counts, which were normalized using the DESeq2 package (v1.50.2) in R. Principal component analysis (PCA) was performed based on normalized gene expression data to assess sample clustering. Differentially expressed genes (DEGs) were identified using DESeq2. Kyoto Encyclopedia of Genes and Genomes (KEGG) pathway enrichment analysis of DEGs was performed using the clusterProfiler package in R. To determine whether transcriptional changes in BV2 cells were associated with MS-like microglial activation, we curated an MS-associated microglial signature gene set consisting of disease-associated microglial markers, complement and innate immune genes, inflammatory cytokines and chemokines, antigen-presentation and interferon-responsive genes, lipid-metabolism and myelin-debris-handling genes, oxidative-stress-related genes, and proliferation-associated reactive microglial

genes (Supplemental Table 2). For each comparison, genes were ranked by log_2 fold change, and enrichment of the MS-associated microglial signature was assessed using the fgsea package in R with 10,000 permutations, a minimum gene-set size of 5, and a maximum gene-set size of 500.

Isolation of mouse naïve CD4$^+$ T cells and T cell differentiation

Spleens were harvested from mice of the indicated genotypes, and single-cell suspensions were prepared. Mouse naïve CD4$^+$ T cells were isolated from splenocytes by magnetic negative selection using the EasySep™ Mouse Naïve CD4$^+$ T Cell Isolation Kit (STEMCELL, Cat. #: 19765). Purified naïve CD4$^+$ T cells were cultured in X-VIVO 15 medium supplemented with 10% FBS, 1% penicillin–streptomycin, and 50 µM 2-mercaptoethanol.

To induce differentiation into specific T cell subsets, cells were additionally stimulated with CD3/CD28 T Cell Activator (STEMCELL, Cat. #: 100-1572) and the following cytokines and neutralizing antibodies were added to the culture medium as previously described (3, 4). For Th1 differentiation, cells were cultured with 10 ng/mL IL-2, 10 ng/mL IL-12 (PeproTech, Cat. #: 210-12), and 10 µg/mL anti–IL-4 (Invitrogen, Cat. #: 16-7041-81, clone: 11B11). For Th2 differentiation, cells were cultured with 10 ng/mL IL-2, 10 ng/mL IL-4 (PeproTech, Cat. #: 214-14), and 10 µg/mL anti–IFN-γ (Invitrogen, Cat. #: 16-7311-81, clone: R4-6A2). For Th17 differentiation, cells were cultured with 5 ng/mL TGF-β (PeproTech, Cat. #: 100-21), 20 ng/mL IL-6 (PeproTech, Cat. #: 216-16), 10 ng/mL IL-23 (PeproTech, Cat. #: 200-23), 10 ng/mL IL-1β

(PeproTech, Cat. #: 211-11B), 10 μg/mL anti–IL-4, 10 μg/mL anti–IFN-γ, and 10 μg/mL anti–IL-2 (Invitrogen, Cat. #: 16-7021-85, clone: JES6-5H4). For Treg differentiation, cells were cultured with 10 ng/mL IL-2, 10 ng/mL TGF-β, 10 μg/mL anti–IL-4, and 10 μg/mL anti–IFN-γ.

Th1/Th17-BV2 and naïve CD4-BV2 co-culture systems

For co-culture experiments, a mixed medium consisting of DMEM and X-VIVO 15 at a 1:1 ratio supplemented with 10 ng/mL IL-2 was used. BV2 cells were detached, collected by centrifugation, resuspended in co-culture medium, and adjusted to a concentration of 5×10^5 cells/mL. In vitro–polarized Th1 and Th17 cells were harvested, resuspended in co-culture medium, mixed at a 1:1 ratio, and adjusted to a final concentration of 1×10^5 cells/mL. Naïve CD4 T cells were similarly collected and resuspended in co-culture medium at a concentration of 1×10^5 cells/mL.

BV2 cells were co-cultured with either Th1/Th17 cells or naïve CD4 T cells at a volume ratio of 1:1 for 24h. Treg cells were then harvested, resuspended in co-culture medium, and adjusted to a concentration of 1×10^6 cells/mL. The required number of Treg cells was prepared and the volume was adjusted to 500 μL, which was subsequently added to the Th1/Th17–BV2 or naïve CD4–BV2 co-culture systems for an additional 48h. Where indicated, anti-CD3 and anti-CD28 antibodies were added to the co-culture system at a 1:100 dilution (STEMCELL, Cat. #: 100-1572) to provide primary and co-stimulatory signals for T cell activation.

siRNA transfection of Treg cells

Treg cells were transfected with siRNA using the ProteanFect™ Max Mouse Immunocyte Transfection Kit (NANOPORTAL BIOTECH, Cat. #: PT03) according to the manufacturer's instructions. Briefly, 2 × 10^5 Treg cells were transfected with 20 pmol siRNA per reaction. The ProteanFect transfection complex was prepared as follows: 20 pmol siRNA was added to 40 µL Reagent A and mixed thoroughly, followed by the addition of 0.7 µL Reagent B. The mixture was gently pipetted up and down 20–30 times or vortexed for 10 s to ensure complete mixing. Subsequently, 10 µL Reagent C was added and mixed thoroughly.

For transfection, Treg cells were collected by centrifugation at 300 × g for 5 min, washed twice with Opti-MEM, and finally resuspended in Opti-MEM at a concentration of 1 × 10^7 cells/mL. Twenty microliters of the cell suspension were mixed with the prepared ProteanFect transfection complex and incubated at 37°C for 30 min in centrifuge tubes. After incubation, approximately five volumes of complete culture medium were added, followed by centrifugation at 300 × g for 5 min. The supernatant was discarded, and cells were resuspended in complete culture medium for further culture.

Transfection efficiency and gene knockdown efficiency were assessed 48 h after siRNA transfection. All subsequent experiments were performed within 5 days after transfection. The sequences of siRNAs used in this study are listed in Supplemental Table 7.

Pooled shRNA screening

The pooled shRNA library targeting over 2,232 mouse genes (Supplemental Table 3), with six shRNA constructs per gene from the RNAi Consortium, was cloned into a lentiviral vector backbone containing an EGFP-coding sequence expressed as a bicistronic transcript together with the shRNA. Lentiviral particles were produced in 293T cells, and mouse natural Treg cells were transduced at a multiplicity of infection of below 0.1 as assessed by EGFP expression. Three days after transduction, EGFP+ Treg cells were sorted and transferred into either the Th1/Th17-BV2 coculture system or the naïve CD4-BV2 control system. After 48 hours of coculture, EGFP+ Treg cells were sorted again using a gating strategy incorporating live/dead discrimination and CD25 staining for downstream DNA sequencing.

Genomic DNA was extracted from the sorted cells, and shRNA cassettes were amplified by PCR using primers (F: CTTGGCTTTATATATCTTGTGGAAAGGACGAAACACCG; R: GCGCAACCCCAACTTTTCTATAC). Sequencing libraries were generated following Illumina protocols. Sequence data were analyzed using the Barcode Deconvoluter software (Cellecta), and read counts for each shRNA construct were quantified. Read counts were normalized to the total number of reads per sample. For each gene, the mean fold change and corresponding p-value (Student's t-test) comparing the Th1/Th17-BV2 and naïve CD4-BV2 coculture systems were calculated. The results of the shRNA screen are provided in

Supplementary data set 1.

Induction of active EAE

Experimental autoimmune encephalomyelitis (EAE) was induced as previously described. Briefly, mice were immunized subcutaneously with 300 ng of MOG_{35-55} peptide emulsified at a 1:1 ratio in complete Freund's adjuvant (CFA) containing 1 mg/mL Mycobacterium tuberculosis, in a total volume of 300 μL per mouse. Pertussis toxin (200 ng per mouse) was administered intravenously on days 0 and 2 post-immunization (5). Mice were monitored daily for clinical signs of EAE and assigned a clinical score as follows: 0 – No clinical signs; 1 – Partial tail paralysis; 2 – Complete tail paralysis; 3 – Hind limb weakness (paresis) and uncoordinated movement; 4 – Paralysis of one hind limb; 5 – Paralysis of both hind limbs; 6 – Paralysis of both hind limbs with weakness in the forelimbs; 7 – Paralysis of both hind limbs and one forelimb; 8 – Paralysis of all four limbs; 9 – Moribund state; 10 – Death. This scoring system was used to assess disease progression throughout the study (6).

Isolation of infiltrated cells from spinal cord

Spinal cords were harvested from EAE mice on day 14 post-immunization and immediately placed in ice-cold RPMI-1640 medium. The tissues were finely minced with sterile blades and transferred to centrifuge tubes. Tissue suspensions were enzymatically digested in RPMI-1640 medium containing 10 μg/mL DNase I, 10 μg/mL collagenase II, and 10 μg/mL papain at 37°C for 20

min. Following digestion, bovine serum albumin (BSA) was added to a final concentration of 20% (w/v). The suspensions were gently triturated several times and centrifuged at 1,360 × g for 20 min at 4°C to remove myelin. The resulting cell pellets were resuspended in RPMI-1640 medium and centrifuged at 500 × g for 10 min at 4°C. After removal of the supernatant, the isolated cells were collected for downstream analyses.

Flow Cytometry

Single-cell suspensions were prepared from peripheral blood, spleen, and spinal cord. Splenic single-cell suspensions were generated by gently dissociating the tissue between frosted glass slides. Erythrocytes in blood and spleen samples were lysed using Red Cell Lysis Buffer (Invitrogen, Cat. #: 00-4300-54), and all cell suspensions were filtered through 40-μm cell strainers to remove debris.

For intracellular cytokine staining, cells were stimulated for 4 h at 37°C in a humidified incubator with eBioscience™ Cell Stimulation Cocktail containing protein transport inhibitors (7). Cells were then stained with fluorochrome-conjugated antibodies against cell surface markers in FACS buffer. Following washing with 1× PBS, cells were fixed with 2% paraformaldehyde and permeabilized with 0.1% saponin buffer for intracellular staining or with Foxp3/Transcription Factor Staining Buffer for nuclear staining, followed by incubation with the indicated antibodies.

Immunohistochemistry

Mice were perfused with cold PBS followed by 4% paraformaldehyde. The spinal cord was then dissected and immersed in the same fixative for 24 hours at 4°C. After fixation, the spinal cord was washed twice with PBS and dehydrated sequentially in 10% and 30% sucrose solutions. Once dehydrated, the spinal cord was rapidly frozen in OCT compound on dry ice and serially sectioned for immunohistochemistry. The spinal cord sections were blocked for 2 hours at room temperature with a solution of PBS containing 0.1% Triton X-100 and 5% normal goat serum (NGS). After blocking, the sections were incubated with primary antibodies overnight at 4°C. The following day, the sections were thoroughly washed and then incubated with secondary fluorescent antibodies at room temperature for 2 hours (8).

Imaging flow cytometry

Single-cell suspensions were prepared from the peripheral blood of FOXP3-IRES-GFP mice. Cells were first stained with PE/Cyanine7-conjugated anti-mouse CD4 antibody (1:500, BioLegend, Cat. #100422, Clone GK1.5) for 30 min at 4°C, followed by fixation with 2% paraformaldehyde for 30 min. After fixation, cells were permeabilized and incubated for 1 h in staining buffer (PBS containing 0.1% saponin and 1% FBS) with unconjugated primary antibodies against RIPK1 (1:400, Biolynx, Cat. #BX60005, Clone YJY-4-3), MLKL (1:400, CST, Cat. #37705, Clone D6W1K), phospho-RIPK1 (1:400, CST, Cat. #53286, Clone E7G6O), phospho-RIPK3 (1:200, CST, Cat. #91702, Clone E7S1R),

phospho-MLKL (1:400, CST, Cat. #37333, Clone D6E3G), or ASC (1:600, Abcam, Cat. #RM1049). After two washes with staining buffer, cells were incubated with Alexa Fluor 647-conjugated anti-rabbit IgG secondary antibody (1:500) for 30 min, washed, and analyzed by flow cytometry.

For imaging flow cytometry, the cells were resuspended in PBS and analyzed using the ImageStream X MK II system at ×60 magnification (Amnis). Data acquisition was carried out using INSPIRE v.2 software, and analysis was performed using IDEAS v.6.3 (Amnis). Single cells were gated based on area and aspect ratio (9). The expression of CD4, Foxp3, pRIPK1, pRIPK3 or pMLKL was quantified based on fluorophore intensity. The aggregation of RIPK1, MLKL, or ASC was gated based on contrast features, and quantification was performed by determining the proportion of cells exhibiting distinct plaque-like structures relative to the total cell count.

CyTOF detection and cell subset identification

Heparinized blood from MS patients and healthy volunteers were obtained after written informed consent and the approval of the Ethics Committees of Renji Hospital in Shanghai. Lysis buffer was used to remove red blood cells according to the manufacturer's protocol (eBioscience, Cat. #: 00-4300-54). The remaining white blood cells were then stored in freezing media (Stemcell Tech.) as frozen aliquots in liquid nitrogen, with each tube containing 0.5 million cells. The antibodies for RIPK1, pRIPK1, pMLKL, cCasp3 and pIKK were conjugated in-house with elements 169Tm, 175Lu, 176Yb, 157Gd and 139La using a

metal-chelating polymer generously provided by Polaris Biology. Other metal-conjugated antibodies were purchased from Polaris Biology.

The cells were thawed and centrifuged to resuspend in 400 μl of RPMI 1640 medium. Subsequently, the cells were incubated with cisplatin for live/dead cell discrimination (Polaris Biology, Cat. #: PB002) and then subjected to surface marker staining as outlined in the resource table. After washing, the cells were fixed and permeabilized using reagents from Polaris Biology (PB007, PB003, and PB018) for 10 minutes each at room temperature. Intracellular staining protocols were performed for RIPK1, pRIPK1, pMLKL, cCasp3, pIKK, Granzyme B, Perforin, and DNA. CytoNorm was used to normalize batch effects (10). FlowJo software was employed to exclude debris (DNA-), cell doublets (DNA high), dead cells (cisplatin+) and granulocytes (CD66b+). PBMC populations were identified using cell surface markers as shown in Figure S8.

Quantitative real-time PCR analysis

Total RNA was extracted from BV2 cells or spinal cord tissue using TRIzol™ Total RNA Extraction Reagent and quantified with a NanoDrop spectrophotometer. BV2 microglia were isolated from the in vitro co-culture system using anti-CD11b beads (Miltenyi, Cat. #: 130-097-142) and subsequently processed for RNA extraction. One microgram of total RNA was reverse-transcribed into cDNA using oligo(dT) primers and the HiScript III 1st Strand cDNA Synthesis Kit (Vazyme, Cat. #: R312-01), according to the

manufacturer's instructions. Quantitative real-time PCR (RT–qPCR) was performed using 2× Taq Master Mix on a QuantStudio Real-Time PCR System.

Relative mRNA expression levels were calculated using the $2^{-\Delta\Delta Ct}$ method (11), with hypoxanthine–guanine phosphoribosyltransferase (*Hprt*) serving as the internal control. Gene-specific primer sequences are listed in Supplemental Table 8.

Thiamet-G treatment and TNF administration

Thiamet-G (TMG, Selleck, Cat. #: S7213) was dissolved in sterile PBS. For in vitro experiments, cells were treated with 2 μM Thiamet-G for 24 h prior to subsequent treatments. For in vivo experiments, 8–12-week-old mice received daily intraperitoneal injections of TMG at a dose of 20 mg/kg, as previously described (12). PBS or TMG was administered daily for three consecutive days prior to EAE induction or TNF treatment, and daily administration was continued throughout the course of EAE pathogenesis.

Recombinant TNF (Novoprotein, Cat. #: CF09) was reconstituted in PBS at a concentration of 1 mg/mL and further diluted to a non-lethal dose of 4 μg in 100 μL PBS. TNF was administered to mice via tail vein injection.

Diphtheria toxin preparation and treatment

Diphtheria toxin (DT; Sigma, Cat. #: D0564) was dissolved in PBS to prepare a 1 mg/mL stock solution and aliquoted in 10 μL volumes for storage at -80°C. For Treg depletion, mice were intraperitoneally injected with DT at 20 ng/kg

body weight on days 1, 3, and 5. $Foxp3^+$ Treg deletion was assessed 24 h after the final injection. A subsequent round of DT administration was performed on day 12.

Immunoblotting

Th1 and Treg cells cultured in vitro were pre-incubated with Nec-1s (20 μM) or TMG (2 μM), followed by treatment with 100 ng/mL TNF for 12 hours. After treatment, the cells were harvested and lysed using NP40 buffer. The NP40 lysis buffer contained 50 mM Tris (pH 7.5), 130 mM NaCl, 1 mM Na3VO4, 50 mM NaF, 50 mM NEM, 1 mM PMSF, and a 1x protease inhibitor cocktail. The protein concentration in the cell supernatants was measured using a BCA assay and adjusted to ensure equal loading. The proteins were then separated by SDS-PAGE and subjected to immunoblotting with the specified antibodies.

LDH assay

LDH activity in the cell culture supernatants was evaluated using the LDH Cytotoxicity Assay Kit (Beyotime, Cat. #: C0017). Briefly, Th1 and Treg cells were seeded in 96-well plates at appropriate cell densities. The cells were then pre-incubated with either Nec-1s (20 μM), GSK-872 (100 μM), or TMG (2 μM) prior to exposure to 100 ng/mL TNF, 0.1% Triton X-100 as a positive control or a solvent negative control (DMSO or PBS), for a duration of 24 hours. After the treatment period, the cell culture supernatants were collected, and LDH activity was measured according to manufacturer's protocol.

Induction of passive EAE with myelin-reactive T cells

Donor mice were immunized according to the protocol for active EAE induction, except that no pertussis toxin was administered. Eleven days post-immunization, spleens and draining lymph nodes were harvested. Naïve $CD4^+$ T cells were isolated using the EasySep™ Mouse Naïve $CD4^+$ T Cell Isolation Kit (STEMCELL, Cat. #: 19765) and cultured in vitro for 5 days with 20 μg/mL MOG_{35-55} peptide under either Th1 or Th17 polarizing conditions.

After in vitro activation, MOG-specific $CD4^+$ Th1 and Th17 cells were harvested, resuspended in PBS, counted, and intravenously injected into Rag2-KO recipient mice (13, 14). Each recipient received a total of 5×10^6 cells, consisting of equal numbers of Th1 and Th17 cells.

For Treg co-transfer experiments, naïve $CD4^+$ T cells were isolated from WT, RIPK1 D138N, RIPK3-KO or MLKL-KO mice and differentiated in vitro under Treg-polarizing conditions for 5 days. A total of 5×10^6 Treg cells were co-injected via the tail vein into recipient mice simultaneously with the Th1 and Th17 cells.

Treg suppression assay with CellTrace™ Violet–labeled Th1 cells

Th1 cells were washed and resuspended in PBS, then labeled with 5 μM CellTrace™ Violet (CTV; Invitrogen, Cat. #: C34571) for 20 min at 37°C. Labeling was quenched by adding a four-fold excess volume of complete X-VIVO 15 medium containing 10% FBS. After two additional washes with culture

medium, CTV-labeled Th1 cells were seeded at 2×10^5 cells per well in 24-well plates.

Treg cells were differentiated as described above and, where indicated, pre-treated with 2 μM TMG for 24 h. Tregs were then co-cultured with CTV-labeled Th1 cells at ratios of 0:1 or 1:1 in the absence of exogenous stimuli for 3 days. Treg-mediated suppression was assessed as previously described (15). Flow cytometry analysis was performed on a guava easyCyte HT Flow Cytometer (Merck Millipore), and data were analyzed using FlowJo software.

Immunoprecipitation

Treg and conventional $CD4^+$ T (Tcon) cells were sorted from the spleens of Foxp3-IRES-GFP mice using FACS. The sorted cells were lysed in NP-40 lysis buffer containing protease inhibitors and centrifuged at 12,000 rpm for 10 min at 4°C. Protein concentrations in the supernatants were determined using a BCA assay (Thermo Fisher Scientific, Cat. #: 23227) and equalized across samples.

For immunoprecipitation, 1 mL of each lysate was transferred to a new 1.5 mL microcentrifuge tube, and 1 μL of either anti-RIPK1 (santa-crus, Cat. #: sc-133102, Clone: C-12) or anti-RIPK3 (santa-crus, Cat. #: sc-374639, Clone: B-2) antibody was added. Samples were incubated overnight at 4°C. The following day, 15 μL of rProtein A/G beads (smart-lifesciences, Cat. #:

SA032005) were added to each tube and incubated at 4°C for at least 4 h. Beads were then collected and washed three times with cold lysis buffer.

The washed beads were boiled with 50 μL of SDS loading buffer for 5 min at 95°C, followed by centrifugation at 1,000 × g for 5 min. Supernatants containing immunoprecipitated proteins were loaded onto SDS-PAGE gels and analyzed by immunoblotting with the indicated antibodies.

Proximity Ligation Assay

Treg and Tcon cells were isolated via FACS from the spleen of Foxp3-IRES-GFP mice. The cells were then subjected to fixation and permeabilization pre-treatment. Following this, Tcon and Treg cells were blocked with Duolink Blocking Solution for 60 minutes at 37 °C. After blocking, the cells were incubated at 4 °C overnight with two primary antibodies raised in different species: rabbit anti-mouse RIPK1 or rabbit anti-mouse RIPK3, and mouse anti-mouse O-GlcNAc. The next day, Duolink In Situ Proximity Ligation Assay (PLA) probes were added to the cells: anti-rabbit PLUS IgG (Sigma, Cat. #: Duo92002) and anti-mouse MINUS IgG (Sigma, Cat. #: Duo92004). The cells were incubated for 1 hour at 37 °C. The PLA signal was detected using Duolink In Situ Detection Reagent Red (Sigma, Cat. #: Duo92008). For signal amplification, ligation reagents containing ligase were added, and the cells were incubated for 30 minutes at 37 °C. Subsequently, amplification reagents containing polymerase were added, followed by an 80-minute incubation at 37 °C. The PLA signals were visualized as red dots using a Leica SP8 confocal

microscope and quantified using ImageJ software (16). Single antibody staining was used for negative control experiments.

Lentivirus construction and cell transfection

To package the pLKO.1 lentiviral vectors carrying OGA shRNA (GAGTACTAACCAATCCAAATT) or control shRNA (AATTCTCCGAACGTGTCACGT), 293T cells were used. The lentiviral supernatants were collected 48 hours post-transfection and filtered through a 0.22 μm filter. For infection, Treg cells were exposed to the viral supernatants in the presence of 10 μg/ml polybrene. After infection, the cells were cultured for an additional two days prior to use.

Construction of the SLE mouse model

The inducible bm12 model is established by transferring lymphocytes from B6(C)-*H2-Ab1*^{bm12}/KhEgJ (bm12) mice, a strain identical to C57BL/6 except for 3 amino acid substitutions on MHC class II, into C57BL/6 (B6) mice as previously reported (17). In brief, spleens were mashed through 40 μm cell strainers using a syringe plunger to create a single-cell suspension. The cells were centrifuged at 400 x g for 5 minutes, and the supernatant was decanted. Subsequently, 30 million lymphocytes from bm12 mice were injected intraperitoneally into B6 mice.

Kidney harvesting and Hematoxylin & Eosin staining

The thoracic cavity of SLE mice was quickly opened to expose the internal organs, and the kidney from one side was collected and fixed in 4% PFA. After washing with PBS and dehydrating through a gradient ethanol series, the tissues were cleared in xylene, embedded in wax, and sectioned into 5 μm slices for pathological examination (18).

The sections were heated at 70°C, deparaffinized in xylene, and rehydrated through a gradient ethanol series, followed by two washes with running water. The sections were then stained with 0.5% hematoxylin for 5 minutes, rinsed under running water, and differentiated in hematoxylin differentiation solution for 2-5 seconds. After another rinse under running water, the sections were blued for 15 minutes. They were then dehydrated using 85% and 95% ethanol for 5 minutes each, followed by staining with 0.05% Eosin for 5 minutes. The sections were further dehydrated twice with absolute ethanol for 5 minutes each. Subsequently, the sections were treated with fresh absolute ethanol for 5 minutes, followed by three changes of carbo-xylol and three changes of xylene. Finally, the sections were mounted in neutral balsam for microscopic observation.

Quantification and Statistical Analysis

Data were analyzed using GraphPad Prism. A two-tailed Student's t test was used for pairwise comparison between two groups. For comparisons involving more than two groups with a single independent variable, one-way ANOVA was performed. For experiments involving two independent variables, two-way

ANOVA was performed, including assessment of the interaction between the two factors. Post hoc multiple-comparisons tests were selected based on the prespecified comparisons: Dunnett's multiple-comparisons test was used when multiple experimental groups were compared with a single predefined control group; Tukey's multiple-comparisons test was used for all pairwise comparisons among groups; and Šídák's multiple-comparisons test was used for prespecified pairwise comparisons. *$p < 0.05$, **$p < 0.01$, ***$p < 0.001$, NS = no significant difference. Differences were considered to be significant if p value < 0.05 (*). Results are presented as mean ± SEM unless further specified. All EAE scoring, image acquisition, image analysis, and Flowjo analysis were performed blinded with respect to mouse genotype, treatment condition and human sample information.

Reagents

A list of reagents and commercial assays, along with their catalog numbers, is provided in Supplemental Table 9.

Human blood samples

The patient studies were conducted in compliance with the Declaration of Helsinki. The inclusion of human subjects and the associated documentation were approved by the Ethics Committees of Shanghai Renji Hospital. The information of patients and healthy controls is provided in Supplemental Table 5. The experiments were conducted with the approval of the Ethics Board at

Renji Hospital in Shanghai. Human Treg cells (Live CD3+CD4+CD25hiCD127lo) and Tcon cells (Live CD3+CD4+CD25neg) used in Supplemental Figure 12 were FACS-sorted from the peripheral blood of healthy donors.

Mice

Wild-type C57BL/6 (CD45.2) mice were purchased from Shanghai Lingchang Biotech Co. Ltd. Wild-type C57BL/6 (CD45.1), RAG2-KO, Foxp3-IRES-DTRGFP, R26-CAG-Cas9, Foxp3-IRES-tdTomato and R26-EGFP(M-NSG) mice were purchased from Shanghai Model Organisms Center. B6(C)-H2-Ab1bm12/KhEgJ mice were purchased from The Jackson Laboratory. RIPK1 D138N, RIPK3-KO, MLKL-KO mice were kindly provided by Dr. Junying Yuan. RIPK1 S332A mice were kindly provided by Dr. Daichao Xu. Male mice at age of 8-12 week were used in this study. All mice were maintained under pathogen-free conditions and housed with no more than five animals per cage under standardized light-dark cycle conditions with ad libitum access to food and water. The vivarium was maintained under controlled temperature (21 ± 1°C) and humidity (50 – 60%). The experimental procedures used in this study were approved by the Ethics Committees of Interdisciplinary Research Center on Biology and Chemistry (IRCBC), Chinese Academy of Sciences.

References

1. Webster KE, Walters S, Kohler RE, Mrkvan T, Boyman O, Surh CD, et al. In vivo expansion of T reg cells with IL-2-mAb complexes: induction of resistance to EAE and long-term acceptance of islet allografts without immunosuppression. *J Exp Med.* 2009;206(4):751-60.
2. Xie X, Liu J, Liang W, Zhang Y, Gong X, Yuan S, et al. Repression of RIPK1 kinase by INPP5D inhibits expression of diverse proinflammatory mediators and late-onset Alzheimer's disease risk factors. *Immunity.* 2026;59(2):419-37 e11.
3. Qian Y, Arellano G, Ifergan I, Lin J, Snowden C, Kim T, et al. ZEB1 promotes pathogenic Th1 and Th17 cell differentiation in multiple sclerosis. *Cell Rep.* 2021;36(8):109602.
4. Baixauli F, Piletic K, Puleston DJ, Villa M, Field CS, Flachsmann LJ, et al. An LKB1-mitochondria axis controls T(H)17 effector function. *Nature.* 2022;610(7932):555-61.
5. Stromnes IM, and Goverman JM. Active induction of experimental allergic encephalomyelitis. *Nat Protoc.* 2006;1(4):1810-9.
6. Bittner S, Afzali AM, Wiendl H, and Meuth SG. Myelin oligodendrocyte glycoprotein (MOG35-55) induced experimental autoimmune encephalomyelitis (EAE) in C57BL/6 mice. *J Vis Exp.* 2014(86).
7. Domingues HS, Mues M, Lassmann H, Wekerle H, and Krishnamoorthy G. Functional and pathogenic differences of Th1 and Th17 cells in experimental autoimmune encephalomyelitis. *PLoS One.* 2010;5(11):e15531.
8. Andreadou M, Ingelfinger F, De Feo D, Cramer TLM, Tuzlak S, Friebel E, et al. IL-12 sensing in neurons induces neuroprotective CNS tissue adaptation and attenuates neuroinflammation in mice. *Nat Neurosci.* 2023;26(10):1701-12.
9. Junqueira C, Crespo A, Ranjbar S, de Lacerda LB, Lewandrowski M, Ingber J, et al. FcgammaR-mediated SARS-CoV-2 infection of monocytes activates inflammation. *Nature.* 2022;606(7914):576-84.
10. Van Gassen S, Gaudilliere B, Angst MS, Saeys Y, and Aghaeepour N. CytoNorm: A Normalization Algorithm for Cytometry Data. *Cytometry A.* 2020;97(3):268-78.
11. Angelin A, Gil-de-Gomez L, Dahiya S, Jiao J, Guo L, Levine MH, et al. Foxp3 Reprograms T Cell Metabolism to Function in Low-Glucose, High-Lactate Environments. *Cell Metab.* 2017;25(6):1282-93 e7.
12. Park J, Ha HJ, Chung ES, Baek SH, Cho Y, Kim HK, et al. O-GlcNAcylation ameliorates the pathological manifestations of Alzheimer's disease by inhibiting necroptosis. *Sci Adv.* 2021;7(3).
13. Zhang Q, Liu W, Wang H, Zhou H, Bulek K, Chen X, et al. TH17 cells promote CNS inflammation by sensing danger signals via Mincle. *Nat Commun.* 2022;13(1):2406.
14. Zhang W, Cao X, Zhong X, Wu H, Shi Y, Feng M, et al. SRC2 controls CD4(+) T cell activation via stimulating c-Myc-mediated upregulation of amino acid

transporter Slc7a5. *Proc Natl Acad Sci U S A.* 2023;120(18):e2221352120.
15. Michalek RD, Gerriets VA, Jacobs SR, Macintyre AN, MacIver NJ, Mason EF, et al. Cutting edge: distinct glycolytic and lipid oxidative metabolic programs are essential for effector and regulatory CD4+ T cell subsets. *J Immunol.* 2011;186(6):3299-303.
16. Alam MS. Proximity Ligation Assay (PLA). *Curr Protoc Immunol.* 2018;123(1):e58.
17. Klarquist J, and Janssen EM. The bm12 Inducible Model of Systemic Lupus Erythematosus (SLE) in C57BL/6 Mice. *J Vis Exp.* 2015(105):e53319.
18. Martinez-Blanco A, Dominguez-Pantoja M, Botia-Sanchez M, Perez-Cabrera S, Bello-Iglesias N, Carrillo-Rodriguez P, et al. CD38 Deficiency Ameliorates Chronic Graft-Versus-Host Disease Murine Lupus via a B-Cell-Dependent Mechanism. *Front Immunol.* 2021;12:713697.

Figure legends:

Supplemental Figure 1. EAE-derived Th1 and Th17 cells induce an MS microglial gene signature in BV2 cells.

(A) Schematic illustration of the naïve CD4-BV2 and Th1/Th17-BV2 co-culture systems. BV2 microglia were co-cultured with naïve CD4 T cells or in vitro-primed Th1 and Th17 cells derived from EAE mice at a ratio of 5:1 for 24h. **(B)** Flow cytometric analysis showing the sorting purity of naïve CD4+ T cells (>95%) and the successful in vitro polarization of naïve CD4+ T cells into Th1 and Th17 cells. **(C)** Principal component analysis (PCA) of RNA-seq data from BV2 cells cultured alone, co-cultured with naïve $CD4^+$ T cells, or co-cultured with Th1 and Th17 cells. **(D)** Numbers of upregulated and downregulated differentially expressed genes (DEGs) identified in the indicated pairwise comparisons ($|\log2 \text{ fold change}| > 1$, $padj < 0.05$). **(E)** KEGG pathway enrichment analysis of genes significantly upregulated in the Th1/Th17-BV2 group relative to the Naïve CD4-BV2 group. Upregulated genes were significantly enriched in immune disease- and inflammatory signaling-related pathways. **(F)** Gene-set enrichment analysis comparing genes upregulated in the indicated system with an MS-associated microglial signature gene set. **(G)** Expression levels of inflammatory cytokines in BV2 microglia were quantified by qPCR after 24h of co-culture with naïve CD4 T cells or Th1+Th17 cells ($n = 4$ independent experiments per group). **(H)** Expression levels of inflammatory cytokines in BV2 microglia within the Th1/Th17-BV2 co-culture system were

quantified by qPCR after 48h of co-culture with Treg cells in different ratios (n = 3 independent experiments per group). Mean ± SEM. **p < 0.01 by Student's t test (G).

Supplemental Figure 2. Th1/Th17-driven inflammatory BV2 microenvironment promotes Treg cell death.

(A and B) Flow cytometry gating strategy for CFSE-labeled Treg cells in the Th1/Th17-BV2 co-culture system, with quantification of Treg cell death, and FOXP3 and CD25 expression in surviving Treg cells at a BV2:Treg ratio of 10:0.2 (n = 3 independent experiments per group). **(C)** Flow cytometry analysis of Treg cell death in the Th1/Th17-BV2 co-culture system at different time points, and FOXP3 expression in surviving Treg cells, at a BV2:Treg ratio of 10:0.2 (n = 3 independent experiments per group). **(D and E)** Flow cytometry analysis of Ki-67 expression in surviving Treg cells from the Th1/Th17-BV2 co-culture system, with or without additional CD3/CD28 stimulation (1:100 dilution) (n = 3 independent experiments per group). **(F and G)** Flow cytometric analysis of the transfection efficiency of fluorescently labeled *Ctla4* and *Il10* siRNAs in Treg cells. Untransfected Treg cells were used as controls. CTLA-4 and IL-10 expression levels after siRNA transfection were compared with the siNC group. Mean ± SEM. *p < 0.05, **p < 0.01 by Student's t test (B, D and E) or by Two-way ANOVA with Dunnett's multiple-comparisons test (C).

Supplemental Figure 3. Flow cytometry gating strategy for the pooled shRNA screen in Treg cells.

$CD4^+CD25^+$ Treg cells were isolated and transduced with an EGFP-expressing shRNA library. Live $EGFP^+$ Treg cells were sorted by FACS and co-cultured with Th1/Th17-polarized BV2 cells for 48 h. Following co-culture, live $CD25^{high}EGFP^+$ Treg cells were sorted by FACS for downstream analysis.

Supplemental Figure 4. **Treg cells suppress Th1/Th17-driven microglial inflammation while activating necroptotic signaling in the inflammatory microenvironment.**

(A) FOXP3 expression in natural and induced Treg cells from WT, D138N or MLKL-KO mice was assessed. **(B)** Representative flow cytometry plots showing the purity of isolated primary microglia, assessed by CD11b and IBA-1 staining. **(C)** Expression levels of inflammatory cytokines in primary microglia were quantified by qPCR after 24h of co-culture with naïve CD4 T cells or Th1+Th17 cells (n = 3 independent experiments per group). **(D)** FOXP3 expression in natural and induced Treg cells from WT, RIPK3-KO mice was assessed. **(E)** Flow cytometry analysis of SYTOX Red staining in natural and induced Treg cells within the Th1/Th17-BV2 co-culture system. BV2 and Treg cells were co-cultured at a ratio of 10:0.2 (n = 4 independent experiments per group). **(F)** Expression of inflammatory cytokines in primary microglia from the Th1/Th17 co-culture system. Cytokine expression was quantified by qPCR after 48h of co-culture with natural or induced Treg cells at different primary microglia:Treg ratios (n = 3 independent experiments per group). Mean ± SEM.

*p < 0.05, **p < 0.01 by Student's t test (C and E) or Two-way ANOVA with Dunnett's multiple-comparisons test (F).

Supplemental Figure 5. **Flow cytometry-based detection of activated necroptotic markers in Treg cells.**

(A) Western blot analysis showing pRIPK1 expression in MEFs following 30 min of TOZ treatment (O, 5Z-7-oxozeaenol, 0.25 nM; Z, z-VAD, 50 μM; Nec-1s, 20 μM; T, TNF, 10 ng/mL). pRIPK3 and pMLKL expression were analyzed in 293T cells 18 h after lentiviral transduction with RIPK3 or MLKL, respectively. 293T cells transduced with RIPK3 were pretreated with the RIPK3 inhibitor GSK-872 (100 μM) for 1 h. MLKL S345A was used as a negative control for pMLKL. The same batch of samples validated by western blot was used to validate the flow cytometry protocol for detecting pRIPK1, pRIPK3 and pMLKL. Untreated MEFs or untransfected 293T cells were used as Control. **(B)** Flow cytometric analysis of pRIPK1, pRIPK3, and pMLKL expression in Treg cells from the Th1/Th17–BV2 microglia co-culture system. BV2 microglia were co-cultured with Treg cells at a BV2:Treg ratio of 10:0.2. Treg cells co-cultured with naïve $CD4^+$ T cell–BV2 microglia were used as controls (n = 3 independent experiments per group). Mean ± SEM. *p < 0.05, **p < 0.01 by one-way ANOVA with Sidak's multiple-comparisons test (B).

Supplemental Figure 6. Genetic inhibition of necroptotic signaling does not affect baseline Treg homeostasis but ameliorates EAE severity.

(A) Clinical scores of passive EAE mice that received in vitro primed Th1+Th17 cells along with WT Treg cells, RIPK3-KO Treg cells (n = 6 mice per group). **(B)** Flow cytometry gating strategy for peripheral Treg cells. **(C to E)** Frequency of CD25+ FOXP3+ Treg cells among CD4+ T cells and expression levels of FOXP3, CD25, CTLA-4, and Ki-67 in Treg cells derived from blood, spleen, and thymus. **(F)** Flow cytometry gating strategy of IFN-γ+CD4+ cells, IL-17A+CD4+ cells and FOXP3+CD4+ cells. **(G and H)** The ratios of IFN-γ+CD4+ to FOXP3+CD4+ cells and IL-17A+CD4+ to FOXP3+CD4+ cells in the spinal cord of passive EAE mice at day14 post-injection (n = 3-5 mice per group). Mean ± SEM. *p < 0.05, **p < 0.01 by Student's t test (G and H) or Two-way ANOVA with Tukey's multiple-comparisons test (A).

Supplemental Figure 7. Prevention of Treg cell necroptosis mitigates EAE disease severity.

(A) Cell percentage of IFN-γ+CD4+ Th1 cells, IL-17A+CD4+ Th17 cells and FOXP3+CD4+ Treg cells in the spinal cord of passive EAE mice at day14 post-injection (n = 3-5 mice per group). **(B and C)** Immunofluorescence analysis of the demyelination process induced by passive EAE in the spinal cord of mice, two weeks after injection of in vitro primed Th1+Th17 cells together with WT Treg , RIPK1 D138N or RIPK3-KO Treg cells. The extent of myelination was quantified using the myelin marker MBP (Scale bars: 200 μm, n = 6 mice per group). **(D and E)** IBA-1 and GFAP staining were utilized to quantify microglial and astrocyte activation in the spinal cord of passive EAE mice at day14 post-

injection (Scale bars: 200 μm, n = 6 mice per group). Mean ± SEM. *p < 0.05, **p < 0.01 by Student's t test (A) or by one-way ANOVA with Tukey's multiple-comparisons test (B, C, D and E).

Supplemental Figure 8. Inhibition of Treg cell necroptosis ameliorates EAE severity.

(A and B) IBA-1 and GFAP staining were utilized to quantify microglial and astrocyte activation in the spinal cord of passive EAE mice at day14 post-injection (Scale bars: 200 μm, n = 6 mice per group). **(C and D)** Expression levels of inflammatory cytokines in the spinal cord of passive EAE mice, quantified by qPCR at day14 post-transfer (n = 5 mice per group). Mean ± SEM. *p < 0.05, **p < 0.01 by one-way ANOVA with Tukey's multiple-comparisons test (A, B, C and D).

Supplemental Figure 9. Flow cytometry gating strategy for Treg cells derived from CD45.1+ and CD45.2+ mice. (A) Flow cytometry gating strategy for CD45.1+ WT Treg cells and CD45.2+ MLKL-KO Treg cells in the blood of Rag2-KO mice, with or without co-transfer of primed Th1 and Th17 cells, 14 days post-injection. **(B)** The percentage of CD45.1+ WT Treg cells and CD45.2+ MLKL-KO Treg cells in the spinal cord of Rag2-KO mice, with or without co-transfer of primed Th1 and Th17 cells. Mice were sacrificed after 14 days receiving an equal number of WT Treg cells and MLKL-KO Treg cells. Data were normalized to the number of CD45.2+ MLKL-KO Treg cells in Rag2-KO mice (n = 6 mice per group). **(C)** The expression of Ki-67 in CD45.1+ WT

Treg cells and CD45.2+ MLKL-KO Treg cells in the blood of Rag2-KO mice, with or without co-transfer of primed Th1 and Th17 cells. Mice were sacrificed after 14 days receiving an equal number of WT Treg cells and MLKL-KO Treg cells. Data were normalized to the number of CD45.2+ MLKL-KO Treg cells in Rag2-KO mice (n = 6 mice per group). **(D)** Flow cytometry was used to analyze the normalized ratios of CD45.1+EGFP- Th17 to CD45.1+EGFP- Treg cells and CD45.2+EGFP- Th17 to CD45.2+EGFP- Treg cells in Rag2-KO mice following co-transfer of primed Th1 and Th17 cells. Mice were sacrificed after 14 days receiving an equal number of WT Treg cells and MLKL-KO Treg cells (n = 6 mice per group). Mean ± SEM. NS by Student's t test (B, C and D).

Supplemental Figure 10. Necroptotic signaling is uniquely upregulated in Treg cells during EAE. (A) Flow cytometry gating strategy of Th1, Th2, Th17 and Treg cells. **(B)** Flow cytometry analysis of the expression of pRIPK1, pRIPK3, pMLKL and SYTOX Red in Th1, Th2, Th17 and Treg cells of WT and D138N EAE mice at day0, day7 and day21 post-immunization (n = 3-6 mice per group). Mean ± SEM. *p < 0.05, **p < 0.01 by Student's t test (B).

Supplemental Figure 11. RIPK1 and MLKL staining in Treg and Tcon cells. (A and B) Representative images display RIPK1 and MLKL staining, in circulating Tcons (CD4+FOXP3-) and Treg cells (CD4+FOXP3+) from EAE mice at day0 post-immunization, acquired using imaging flow cytometry. (Scale bars: 7 μm; n = 4 mice per group). Mean ± SEM. NS by Student's t test (B).

Supplemental Figure 12. Flow cytometric analysis of CD4+ T cell subsets in MS patients.

(A) Flow cytometry gating strategy for identifying different leukocyte subsets from human blood samples. **(B)** The statistical graph shows the proportions of Th1, Th2, Th17, and Treg cells in the CD4+ T cells of multiple sclerosis (MS) patients and healthy controls (MS n = 11 patients, healthy controls n = 11 volunteers). **(C to E)** Flow cytometry analysis of pIKK, cCasp3 and RIPK1 expression in Th1, Th2, Th17 and Treg cells of MS patients and healthy controls (MS n = 11 patients and Ctrl n = 11 health volunteers). Mean ± SEM. NS by Student's t test (B).

Supplemental Figure 13. No activation of necroptotic signaling is observed in non-Treg cells from patients with MS.

(A) Percentage of different leukocyte subsets in PBMCs from MS patients and healthy controls (MS n = 11 patients and Ctrl n = 11 health volunteers). **(B to E)** Flow cytometry analysis of pRIPK1, pMLKL, pIKK, cCasp3 in different leukocyte subsets from MS patients and healthy controls (MS n = 11 patients and Ctrl n = 11 health volunteers). **(F)** Ratios of pRIPK1 to RIPK1 in Th1, Th2, Th17, and Treg cells (MS n = 11 patients and Ctrl n = 11 health volunteers). **(G and H)** Flow cytometry analysis of RIPK1 expression and pRIPK1/RIPK1 ratio in different leukocyte subsets from MS patients and healthy controls (MS n = 11 patients and Ctrl n = 11 health volunteers). Mean ± SEM. *$p < 0.05$ by Student's t test (F).

Supplemental Figure 14. Human Treg cells are preferentially susceptible to RIPK1–MLKL-dependent necroptotic activation in Th1/Th17-BV2 co-culture system.

(A) Schematic illustration of the co-culture systems involving human Treg and Tcon cells with naïve CD4–BV2 and Th1/Th17–BV2 microglia systems. **(B)** Flow cytometry gating strategy for human Treg and Tcon cells in the co-culture system. **(C and D)** Flow cytometric analysis of pRIPK1 and pMLKL expression in human Treg and Tcon cells in the co-culture system (n = 3 independent experiments per group). Mean ± SEM. **p < 0.01 by Student's t test (C and D).

Supplemental Figure 15. Soluble TNF induces Treg necroptosis in vivo.

Flow cytometry analysis of pRIPK1, pRIPK3 and pMLKL expression in Th1, Th2, Th17 and Treg cells isolated from the blood of mice, 2 hours after intravenous injection of 4 μg soluble TNF (n = 3 mice per group). Mean ± SEM. **p < 0.01 by one-way ANOVA with Dunnett's multiple-comparisons test.

Supplemental Figure 16. Soluble TNF induces Treg necroptosis in vitro.

(A) Representative images display pRIPK1, pRIPK3 and pMLKL staining in Tcons (CD4+FOXP3-) and Treg cells (CD4+FOXP3+) isolated from the blood of mice, 2 hours after intravenous injection of 4 μg TNF. The statistical graph shows the percentage of cells exhibiting pRIPK1, pRIPK3 or pMLKL stainng and the data were normalized to Tcons before TNF injection (Scale bars: 7 μm; n = 4 mice per group). **(B)** Flow cytometry analysis of SYTOX Red expression in Treg cells isolated from mouse blood, 2 hours after intravenous injection of

4 μg TNF (n = 4 mice per group). **(C)** LDH release assay of in vitro cultured Th1 and Treg cells treated with 100 ng/mL TNF. LDH release from Th1 or Treg cells treated with 0.1% Triton X-100 was considered as 100%. Nec-1s (20 μM) and Gsk-872 (100 μM) were used to inhibit RIPK1 and RIPK3 kinase activity, respectively (n = 3-4 independent experiments per group). **(D)** Western blot analysis of necroptotic markers pRIPK1, pRIPK3 (indicated by red arrow), and pMLKL in in vitro cultured Th1 and Treg cells treated with 100 ng/mL TNF for 12 hours. Nec-1s (20 μM) was pretreated 30 minutes prior to TNF administration. Mean ± SEM. *p < 0.05, **p < 0.01 by Student's t test (A, B and C) or one-way ANOVA with Dunnett's multiple-comparisons test (C).

Supplemental Figure 17. The HBP pathway is downregulated in Treg cells. **(A)** Western blot analysis of Glut1 expression in in vitro cultured Th0, Th1, Th2, Th17 and Treg cells. **(B)** FMO control for Glut1 staining. Glut1 expression in peripheral blood Treg cells was analyzed by flow cytometry. For the FMO control, cells were stained with the secondary antibody only, without the anti-Glut1 primary antibody. **(C)** Quantification of Glut1 expression on the cell surface of circulating Th1, Th2, Th17, and Treg cells using flow cytometry (n = 3 mice per group). **(D)** Western blot analysis of A20 expression in in vitro cultured Th0, Th1, Th2, Th17 and Treg cells. **(E)** FMO control for A20 staining. A20 expression in peripheral blood Treg cells was analyzed by flow cytometry. For the FMO control, cells were stained with the secondary antibody only, without the anti-A20 primary antibody. **(F)** Flow cytometry analysis of A20

expression in Th1, Th17 and Treg cells isolated from the blood of WT mice (n = 6 mice per group). **(G)** The diagram depicts the hexosamine biosynthesis pathway (HBP) downstream of glucose uptake. Expression levels of metabolites in the HBP across Th1, Th17, and Treg cells (Data were derived from the supplementary materials published in J Clin Invest. 2015;125(1):194–207. doi:10.1172/JCI76012.). **(H)** Quantification of overall O-GlcNAc modification levels in circulating Th1, Th2, Th17 and Treg cells by flow cytometry (n = 3 mice per group). (**I**) Western blot analysis of overall O-GlcNAc modification levels in in vitro cultured Th0, Th1, Th2, Th17 and Treg cells. Mean ± SEM. *p < 0.05, **p < 0.01 by one-way ANOVA with Dunnett's multiple-comparisons test (C, G and H) or one-way ANOVA with Sidak's multiple-comparisons test (F).

Supplemental Figure 18. O-GlcNAc modification of RIPK3 is downregulated in Treg cells. **(A)** Single antibody incubations were used as negative controls for PLA signaling (Scale bars: 50 μm). **(B to D)** Detection of O-GlcNAc modification on RIPK3 by immunoprecipitation assay or PLA in Tcons (CD4+FOXP3-) and Treg (CD4+FOXP3+) cells. (Scale bars: Left, 50 μm; Right 5 μm; n = 3-4 independent experiments per group). **(E)** Flow cytometry analysis of O-GlcNAcylation levels in Treg cells from the blood of WT mice treated with PBS or 20 mg/kg TMG (n = 9 mice per group). **(F)** Flow cytometry analysis of the percentage of Foxp3-positive Treg cells in the blood of WT mice treated with PBS or 20 mg/kg TMG (n = 9 mice per group). **(G)** CellTrace Violet

(CTV)-labeled Th1 cells were co-cultured with vehicle- or TMG (2 μM, 24h)-pretreated Treg cells for 3 days. The intensity of CTV was measured by flow cytometry. **(H)** Detection of O-GlcNAc modification on RIPK3 using PLA in Tcons and Treg cells isolated from mice pre-treated with 20 mg/kg TMG (Scale bars: Left, 50 μm; Right 5 μm; n = 6-7 independent experiments per group). Mean ± SEM. *p < 0.05, **p < 0.01 by Student's t test (B, D, E, F and H) or Two-way ANOVA with Sidak's multiple-comparisons test (H).

Supplemental Figure 19. Treg necroptosis and EAE symptoms are attenuated via the TMG treatment.

(A) The expression of pRIPK1, pRIPK3 and pMLKL in Treg cells isolated from mice pre-treated with 20 mg/kg TMG at the specified time points after intravenous injection of 4 μg TNF (n = 3 mice per group). **(B)** LDH release assay of in vitro cultured Treg cells pretreated with 2 μM TMG 24 hours, at the indicated time points following exposure to 100 ng/mL TNF (n= 3-4 independent experiments per group). **(C)** Western blot analysis of necroptotic markers pRIPK1, pRIPK3 (denoted by a red arrow) and pMLKL in in vitro cultured Treg cells pretreated with 2 μM TMG for 24 hours and then treated with 100 ng/mL TNF for 12 hours. **(D)** Clinical scores of mice treated with PBS or 20 mg/kg TMG via IP injection one day before immunization with MOG35-55/CFA and PTX. PBS or TMG treatment was administered daily throughout the course of EAE pathogenesis (n= 7-8 mice per group). **(E)** Immunofluorescence analysis of the EAE-induced demyelination process in the spinal cord of mice at day 15

post-immunization, with TMG treatment. The extent of myelination was quantified using the myelin marker MBP (n = 6 mice per group). **(F)** IBA-1 and GFAP staining were utilized to quantify microglial and astrocyte activation in the spinal cord of EAE mice with TMG treatment at day15 post-immunization (Scale bars: Upper, 200 μm; Lower, 20 μm; n = 6 mice per group). Mean ± SEM. *p < 0.05, **p < 0.01 by Student's t test (B, E and F) or Two-way ANOVA with Dunnett's multiple-comparisons test (A and D).

Supplemental Figure 20. TMG treatment suppresses Treg cell necroptosis and ameliorates EAE.

(A) Expression levels of inflammatory cytokines in the spinal cord of PBS or 20 mg/kg TMG treated EAE mice, quantified by qPCR at day15 post-immunization (n = 4 mice per group). **(B)** The ratios of IFN-γ+CD4+ to Foxp3+CD4+ cells and IL-17A+CD4+ to Foxp3+CD4+ cells in the spinal cord of PBS or 20 mg/kg TMG treated EAE mice at day15 post-immunization (n = 6 mice per group). **(C)** Cell percentage of IFN-γ+CD4+ Th1 cells, IL-17A+CD4+ Th17 cells and Foxp3+CD4+ Treg cells in the spinal cord of mice at EAE day15 (n = 6 mice per group). **(D and E)** Representative images display pMLKL staining in circulating Treg cells (CD4+FOXP3+) from PBS or 20 mg/kg TMG treated EAE mice at day7 post-immunization. The statistical graph shows the percentage of cells exhibiting pMLKL staining in Treg cells at day0 and day7 of EAE. Data were normalized to Treg cells at day0 with PBS treatment (Scale bars: 7 μm; n = 6 mice per group). **(F)** Clinical scores of Treg KO mice (Treg were deleted

through treated Foxp3-IRES-DTRGFP mice with 20 ng/g diphtheria toxoid at day1, day3, day5) treated with PBS or 20 mg/kg TMG following immunization with MOG35-55/CFA and PTX (n = 6 mice per group). Mean ± SEM. *p < 0.05, **p < 0.01 by Student's t test (A, B, C and E).

Supplemental Figure 21. Generation of OGA-downregulated Treg cells.

(A) Quantification of OGA expression in Treg cells cultured in vitro two weeks after transduction with lentivirus expressing either shNC or shOGA, as assessed by flow cytometry (n= 4 independent experiments per group). **(B)** Flow cytometry analysis of O-GlcNAcylation levels and Foxp3 expression in shNC Treg cells or shOGA cells (n = 4-6 independent experiments per group). **(C)** The O-GlcNAc level of RIPK1 revealed by PLA in shNC Treg cells or shOGA Treg cells (Scale bars: 5 μm; n = 3 independent experiments per group). **(D)** Flow cytometry analysis of the percentage of IFN-γ- or IL-17A-expressing cells in shOGA Treg cells, shNC Treg cells, and Ctrl Treg cells (n = 4 independent experiments per group). **(E)** Cell percentage of IFN-γ+CD4+ Th1 cells, IL-17A+CD4+ Th17 cells and Foxp3+CD4+ Treg cells in the spinal cord of passive EAE mice at day14 post-injection (n = 6 mice per group). **(F and G)** Flow cytometry was used to assess the expression of pRIPK3 and pMLKL in CD45.1+ shNC Treg cells and CD45.2+ shOGA Treg cells, as well as the ratios of EGFP- Th17 to GFP- Treg cells in Rag2-KO mice following the co-transfer of primed EGFP+ Th1 and Th17 cells. Mice were sacrificed 14 days after receiving an equal number of shNC Treg cells and shOGA Treg cells (n = 3-5

mice per group). Mean ± SEM. *p < 0.05, **p < 0.01 by Student's t test (A, B, C, E, F and G).

Supplemental Figure 22. Treg-intrinsic sgRNA-mediated targeting of RIPK3, MLKL, or OGA ameliorates passive EAE.

(A) Schematic overview of natural Treg cell isolation from Foxp3-Cas9 mice, sgRNA transduction and sorting, followed by co-transfer with EAE-derived Th1/Th17 cells into Rag2-KO recipient mice. **(B)** Flow cytometry gating strategy for sorting Treg cells transduced with AF488-labeled sgRNA. **(C)** Western blot analysis of knockout efficiency in flow-sorted AF488-labeled sgRNA-transduced Treg cells. **(D)** Clinical scores of passive EAE mice that received in vitro primed EAE-derived Th1/Th17 cells together with sgRNA-transduced Treg cells targeting *Ripk3*, *Mlkl*, *Oga*, or control sgRNA-transduced Treg cells (n = 6 mice per group). **(E)** Cell percentage of IFN-γ+CD4+ Th1 cells, IL-17A+CD4+ Th17 cells and Foxp3+CD4+ Treg cells in the spinal cord of passive EAE mice at day14 post-injection (n = 5 mice per group). **(F)** The ratios of IFN-γ+CD4+ to Foxp3+CD4+ cells and IL-17A+CD4+ to Foxp3+CD4+ cells in the spinal cord of passive EAE mice at day14 post-injection (n = 5 mice per group). **(G and H)** Immunofluorescence analysis of spinal cord demyelination in passive EAE mice. (Scale bars: 200 μm; n = 6 mice per group). Mean ± SEM. *p < 0.05, **p < 0.01 by one-way ANOVA with Dunnett's multiple-comparisons test (F), one-way ANOVA with Sidak's multiple-comparisons test (E and H) or Two-way ANOVA with Tukey's multiple-comparisons test (D).

Supplemental Figure 23. Treg-specific sgRNA targeting of RIPK3, MLKL, or OGA ameliorates passive EAE.

(A to C) Immunofluorescence analysis of microglial and astrocyte activation by IBA-1 and GFAP staining at day 14 post-injection, with corresponding quantification. (Scale bars: 200 μm; n = 6 mice per group). **(D)** Expression levels of inflammatory cytokines in the spinal cord of passive EAE mice, quantified by qPCR at day14 post-transfer (n = 5 mice per group). Mean ± SEM. **p < 0.01 by one-way ANOVA with Sidak's multiple-comparisons test (C and D).

Supplemental Figure 24. Flow cytometry gating strategy for immune cell subsets in SLE mice.

Flow cytometry gating strategy for GC B cells, plasma and Tfh cells.

Supplemental Table 1: KEGG pathway analysis of upregulated genes.

Supplemental Table 2: List of MS-associated microglial signatures.

Supplemental Table 3: Gene List for shRNA Library.

Supplemental Table 4: Downregulated and upregulated regulators identified by shRNA screening.

Supplemental Table 5: Age and sex of MS patients and healthy volunteers.

Supplemental Table 6: The sequences of sgRNAs.

Supplemental Table 7: The sequences of siRNAs.

Supplemental Table 8: Primer sequences used for qPCR.

Supplemental Table 9: Reagents.

Supplemental Figure 1

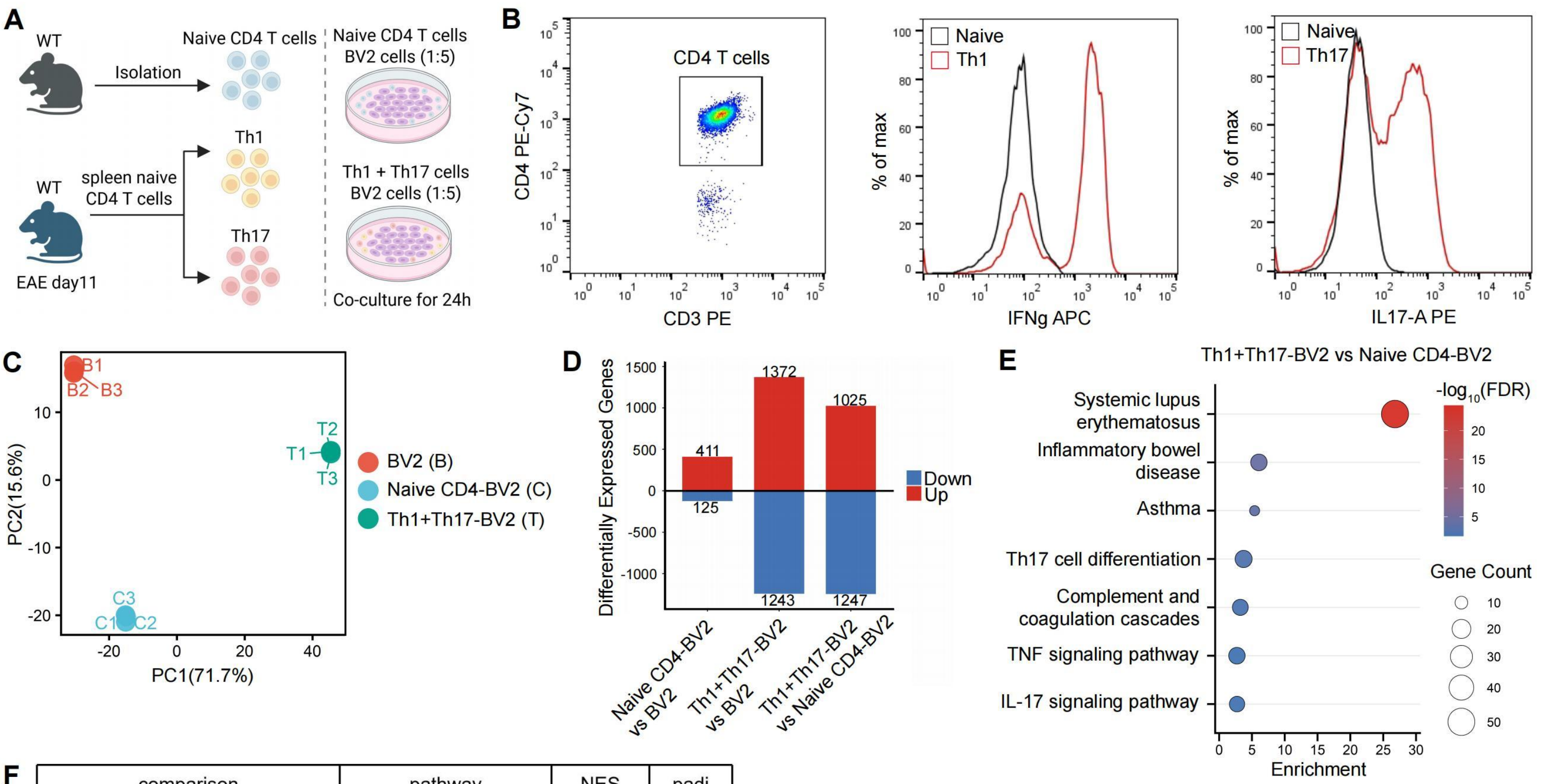

F

comparison	pathway	NES	padj
Th1+Th17-BV2 vs Naive CD4-BV2	MS microglia signature	1.88527	0.0071
Th1+Th17-BV2 vs BV2	MS microglia signature	1.86631	0.0040
Naive CD4-BV2 vs BV2	MS microglia signature	0.84231	0.6935

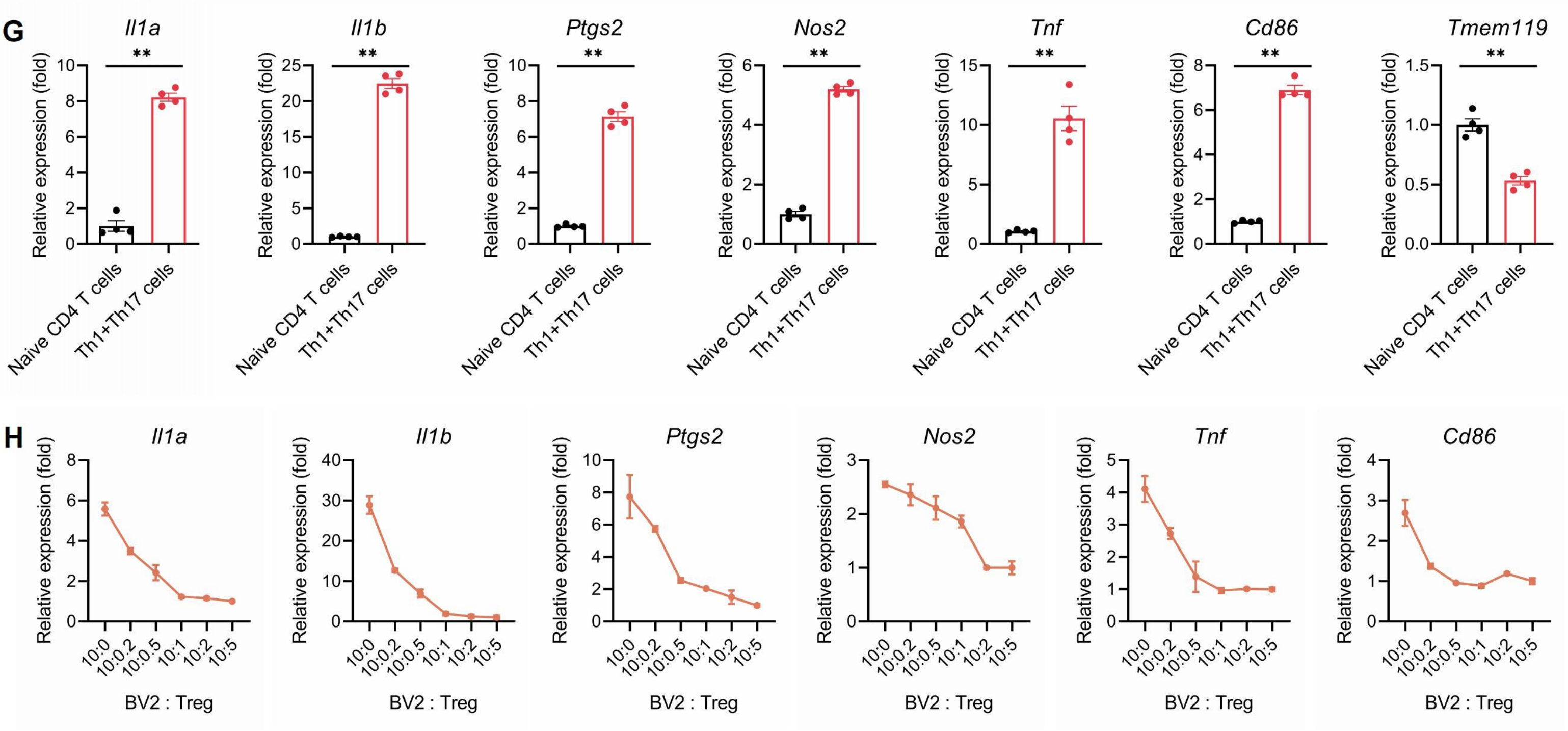

Supplemental Figure 2

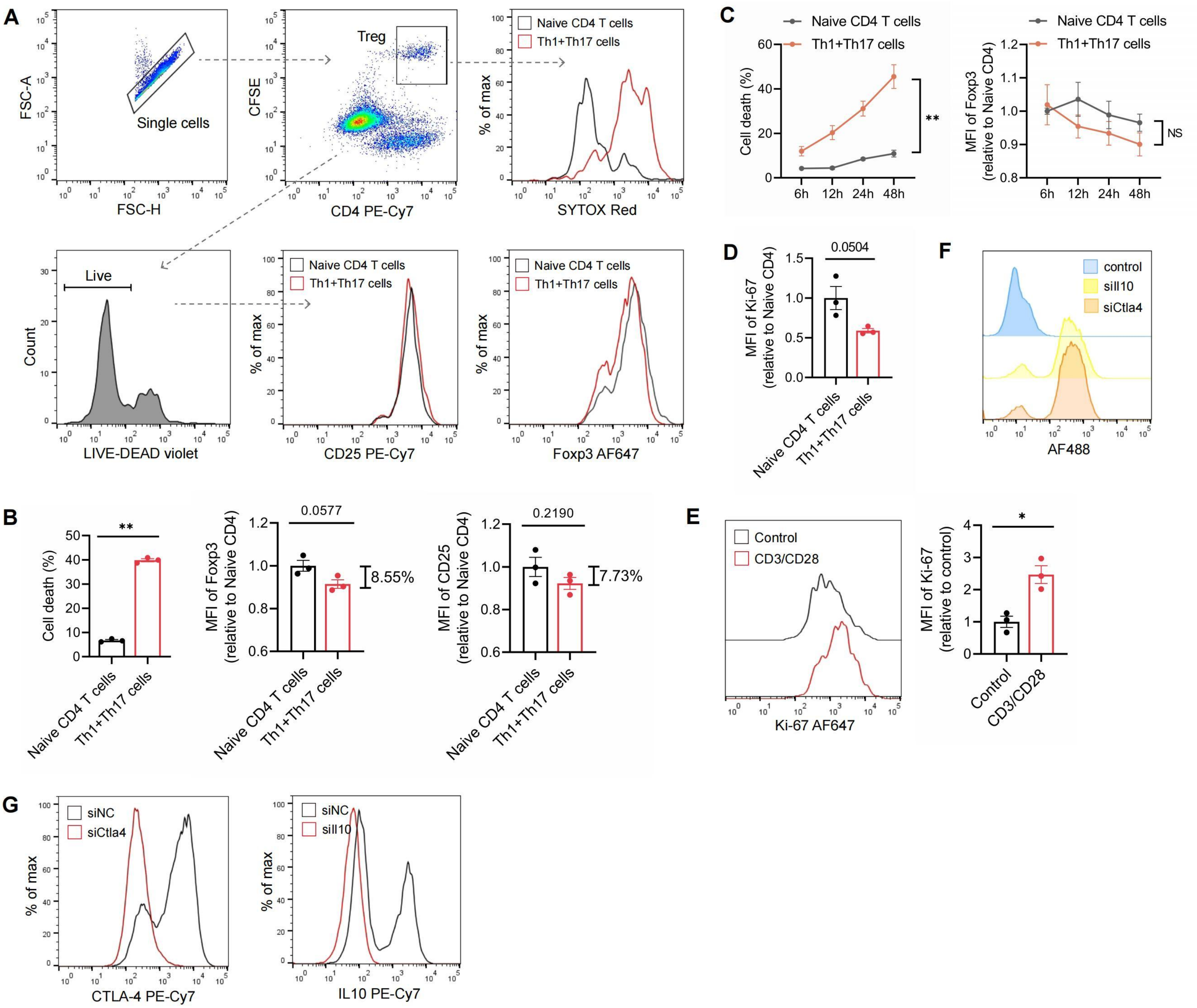

Supplemental Figure 3

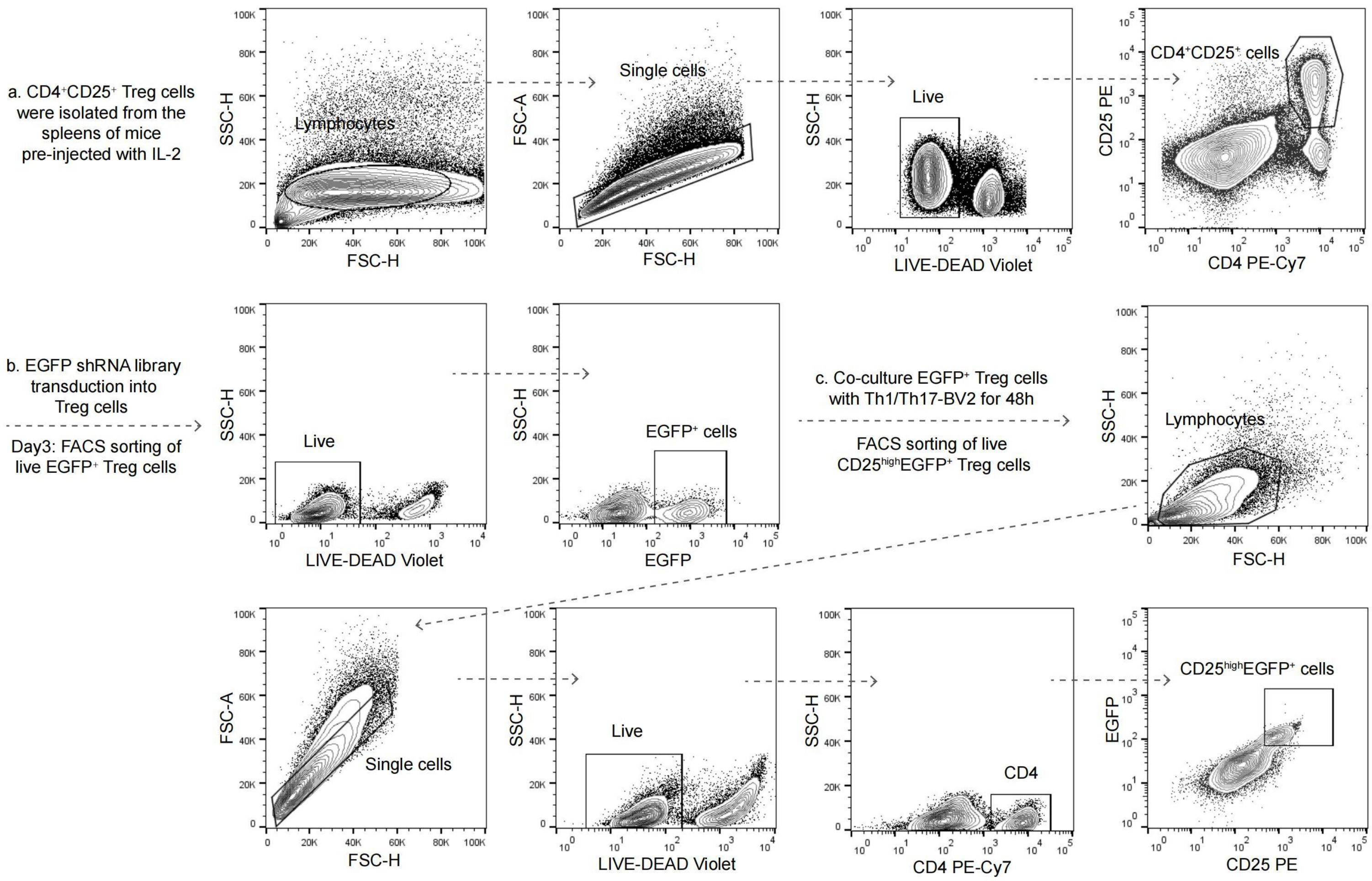

Supplemental Figure 4

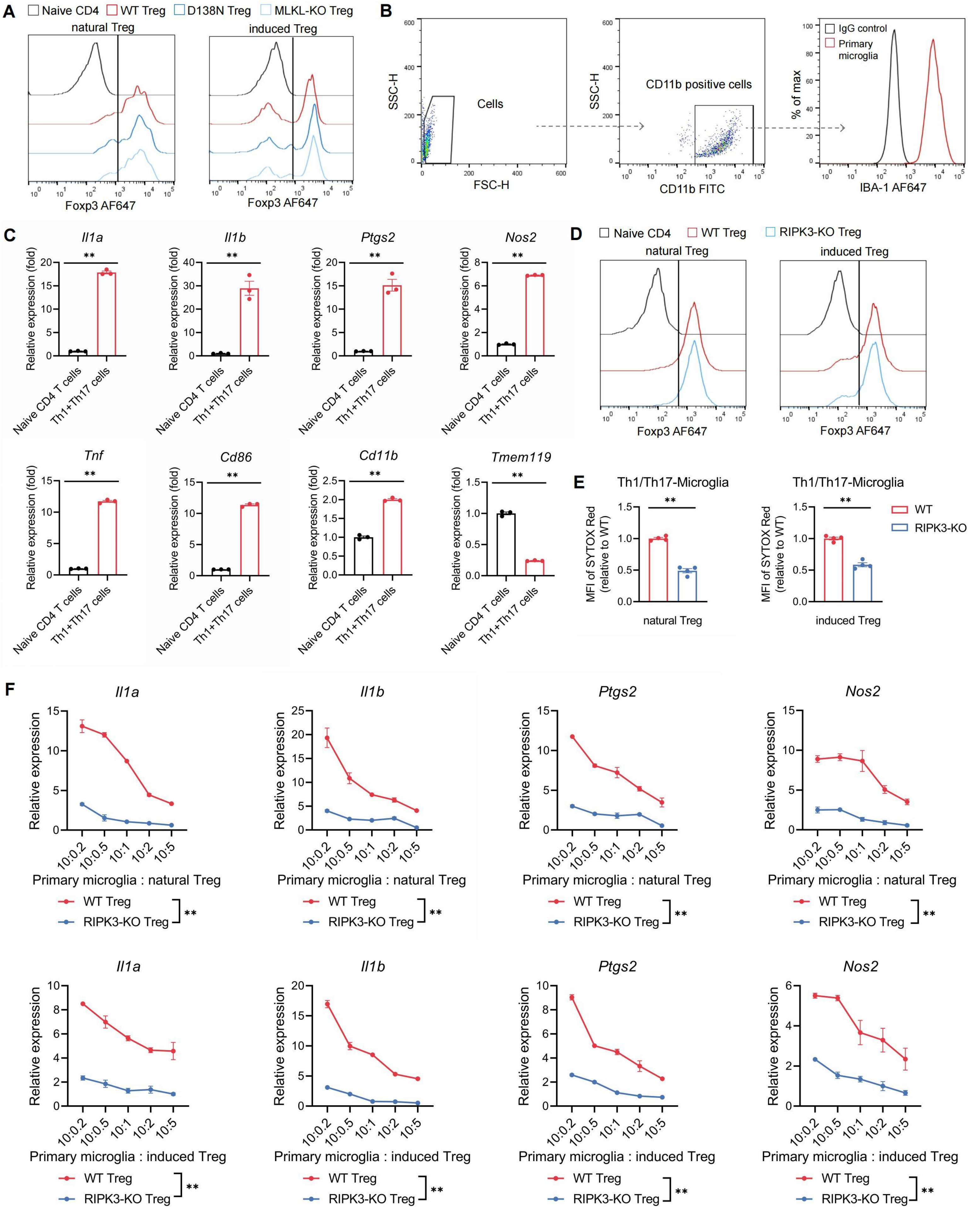

Supplemental Figure 5

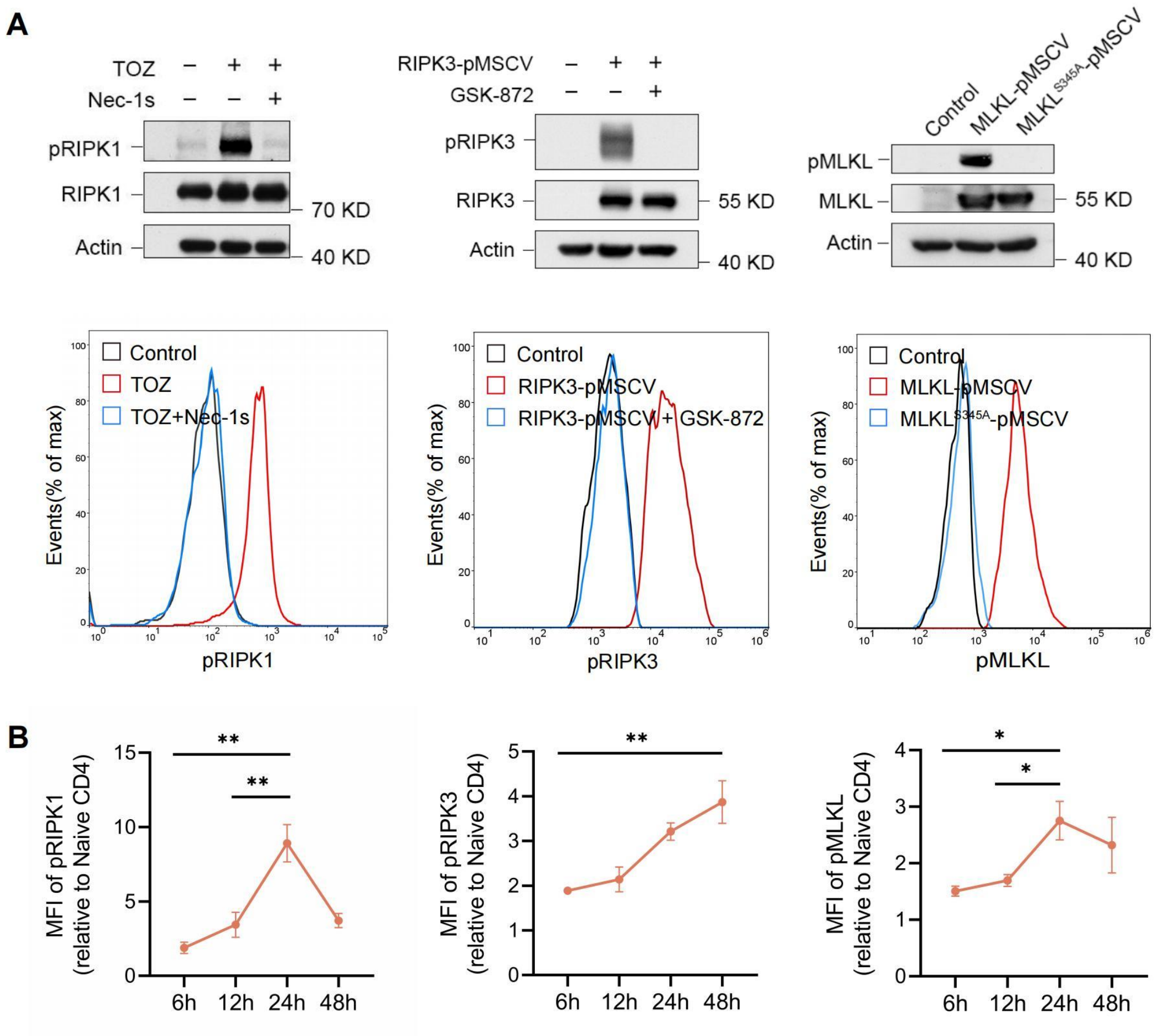

Supplemental Figure 6

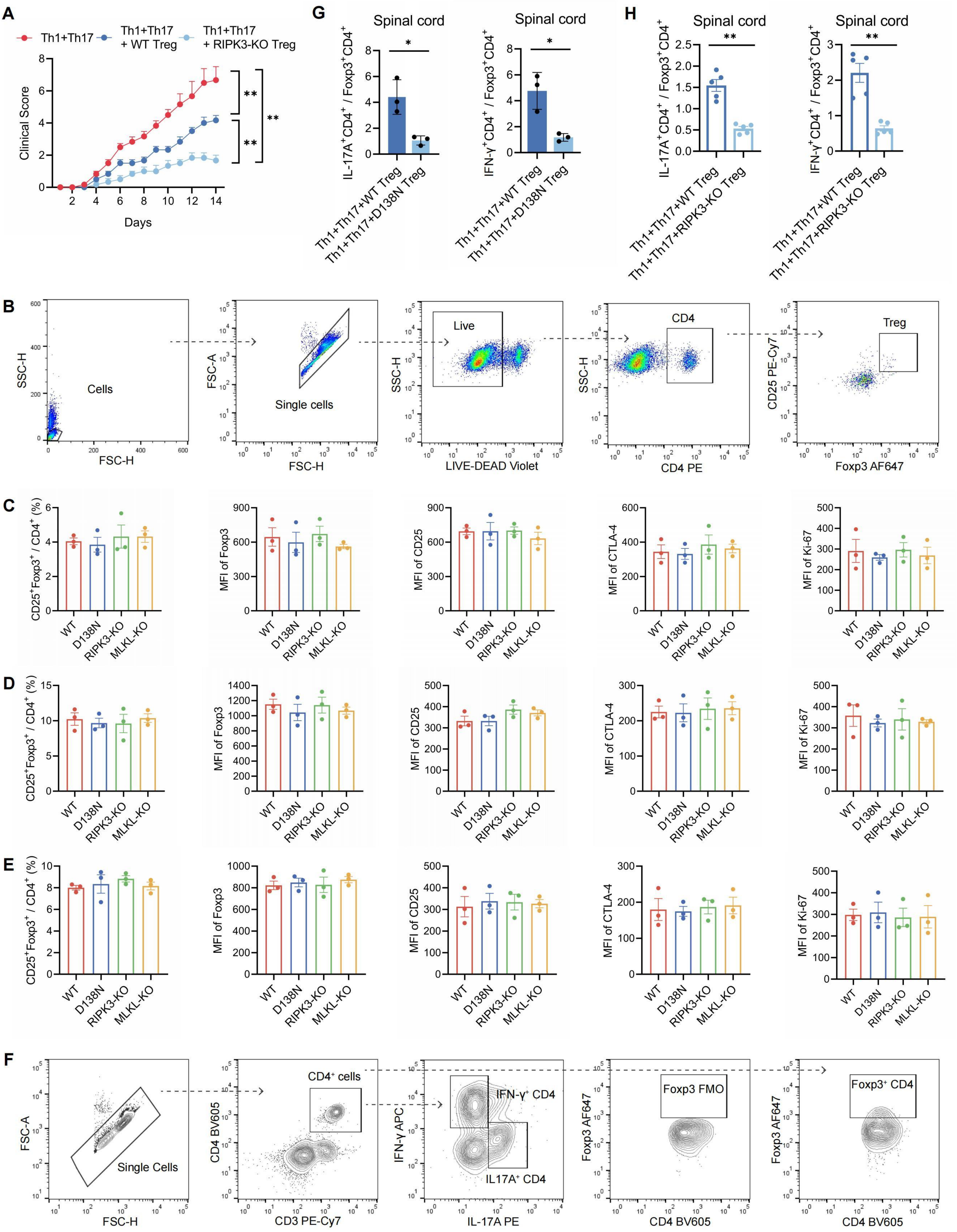

Supplemental Figure 7

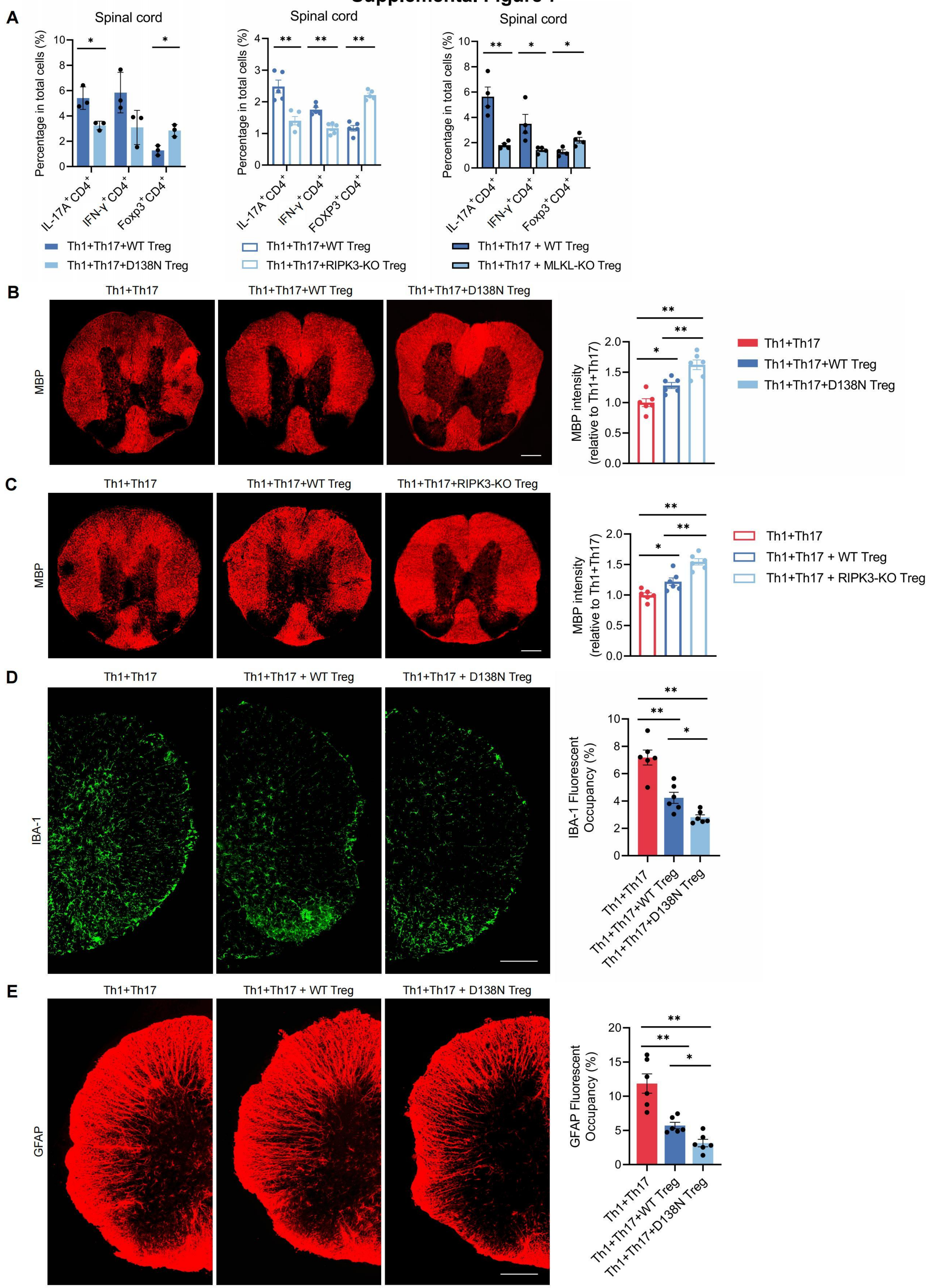

Supplemental Figure 8

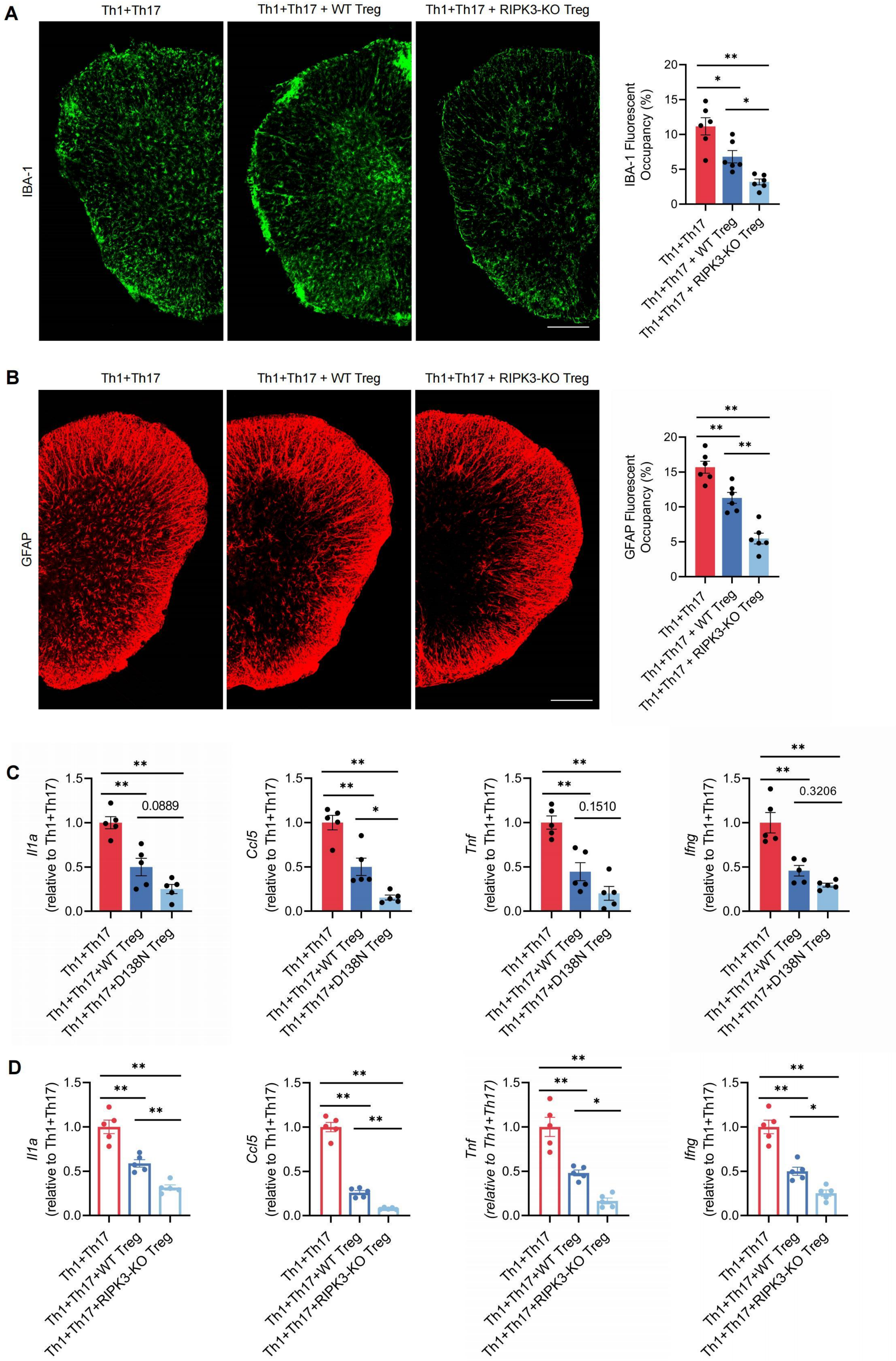

Supplemental Figure 9

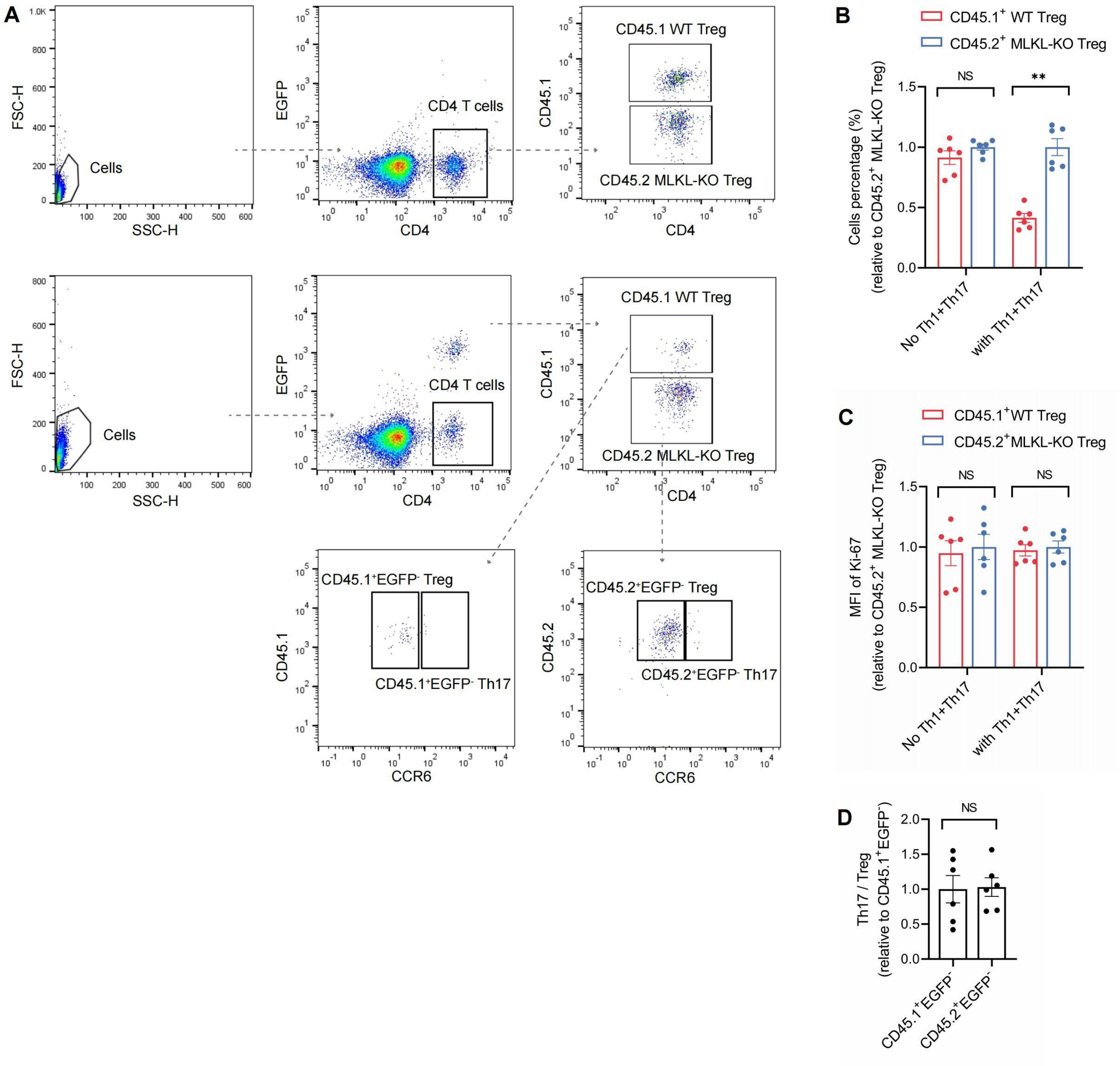

Supplemental Figure 10

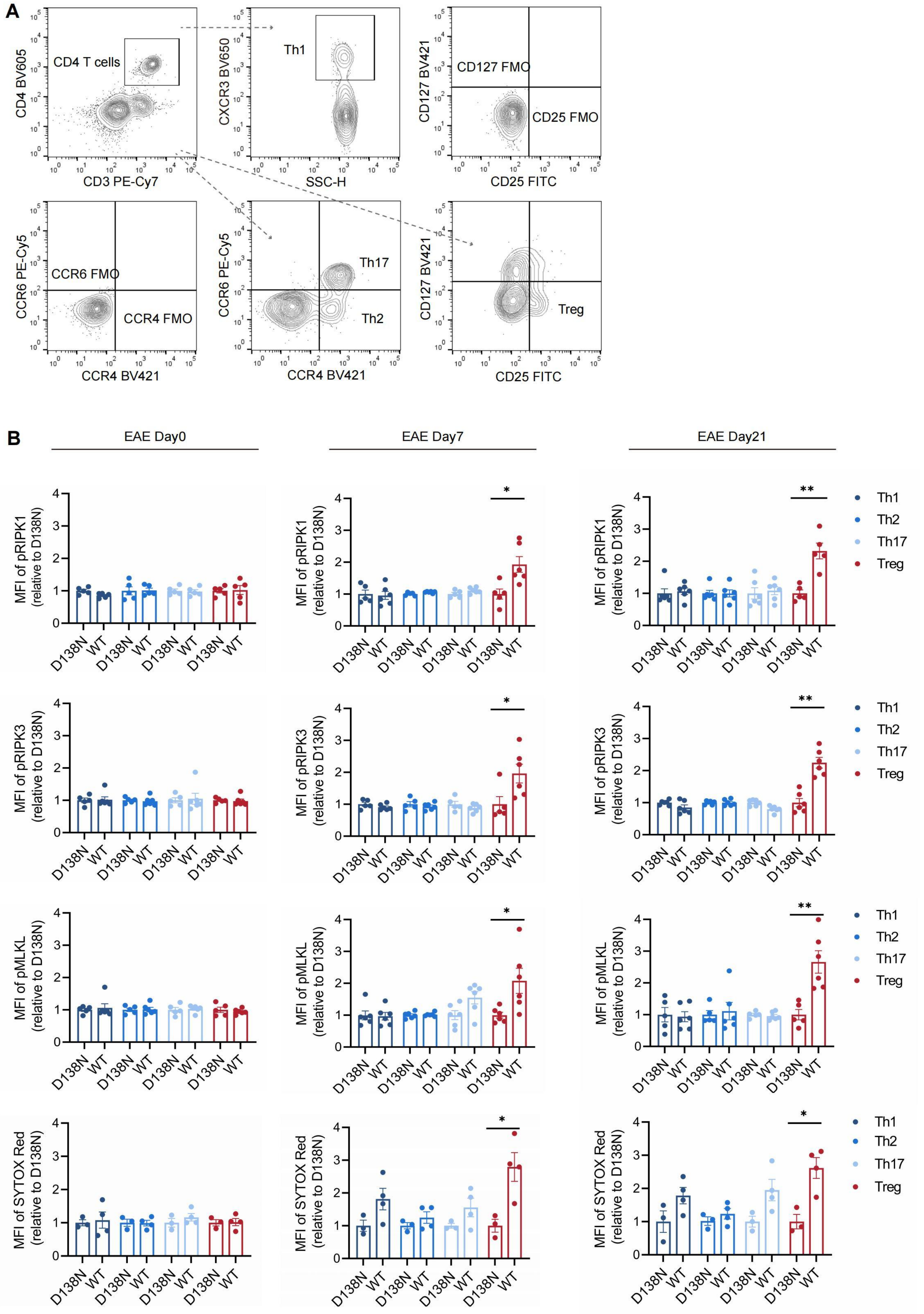

Supplemental Figure 11

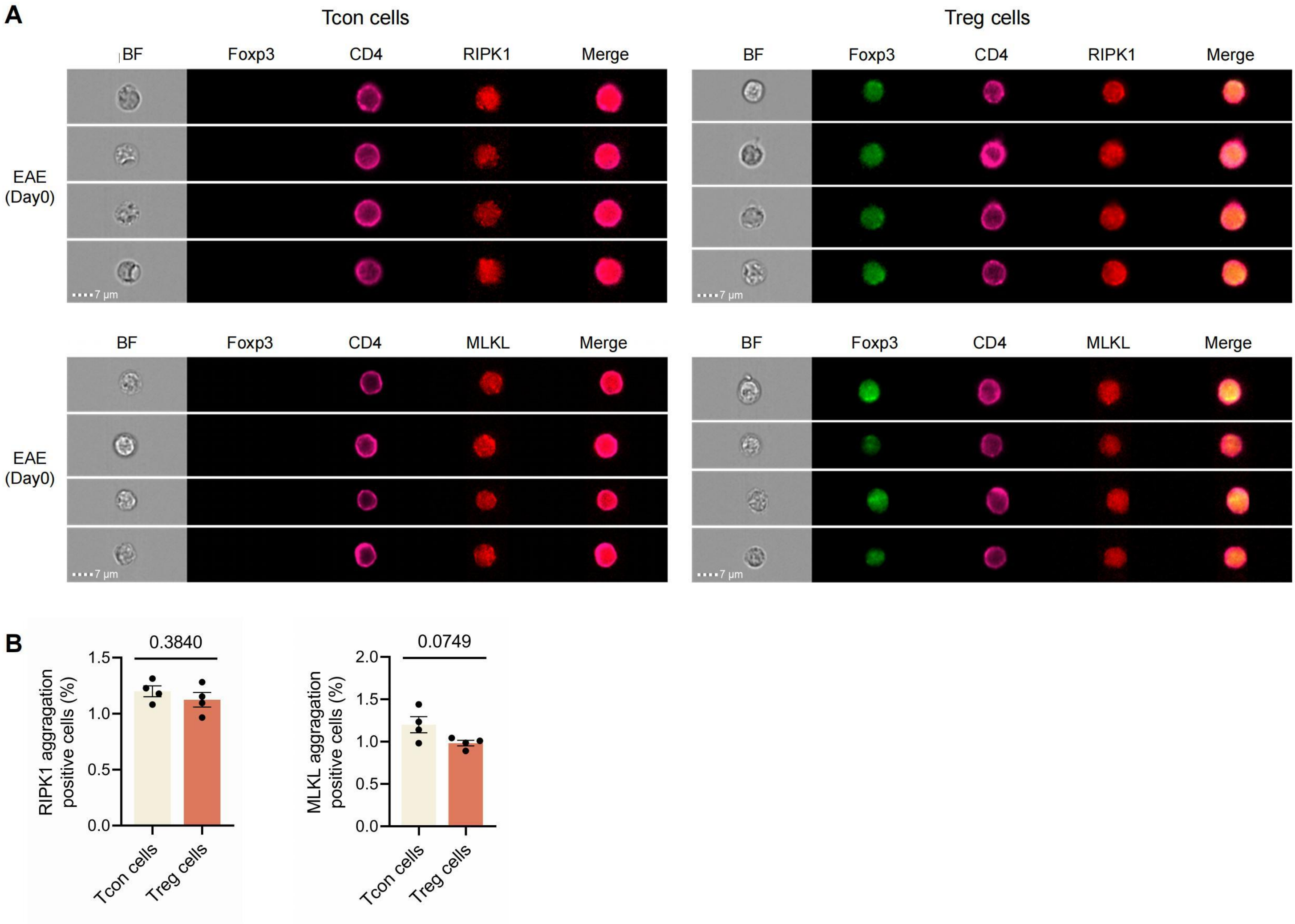

Supplemental Figure 12

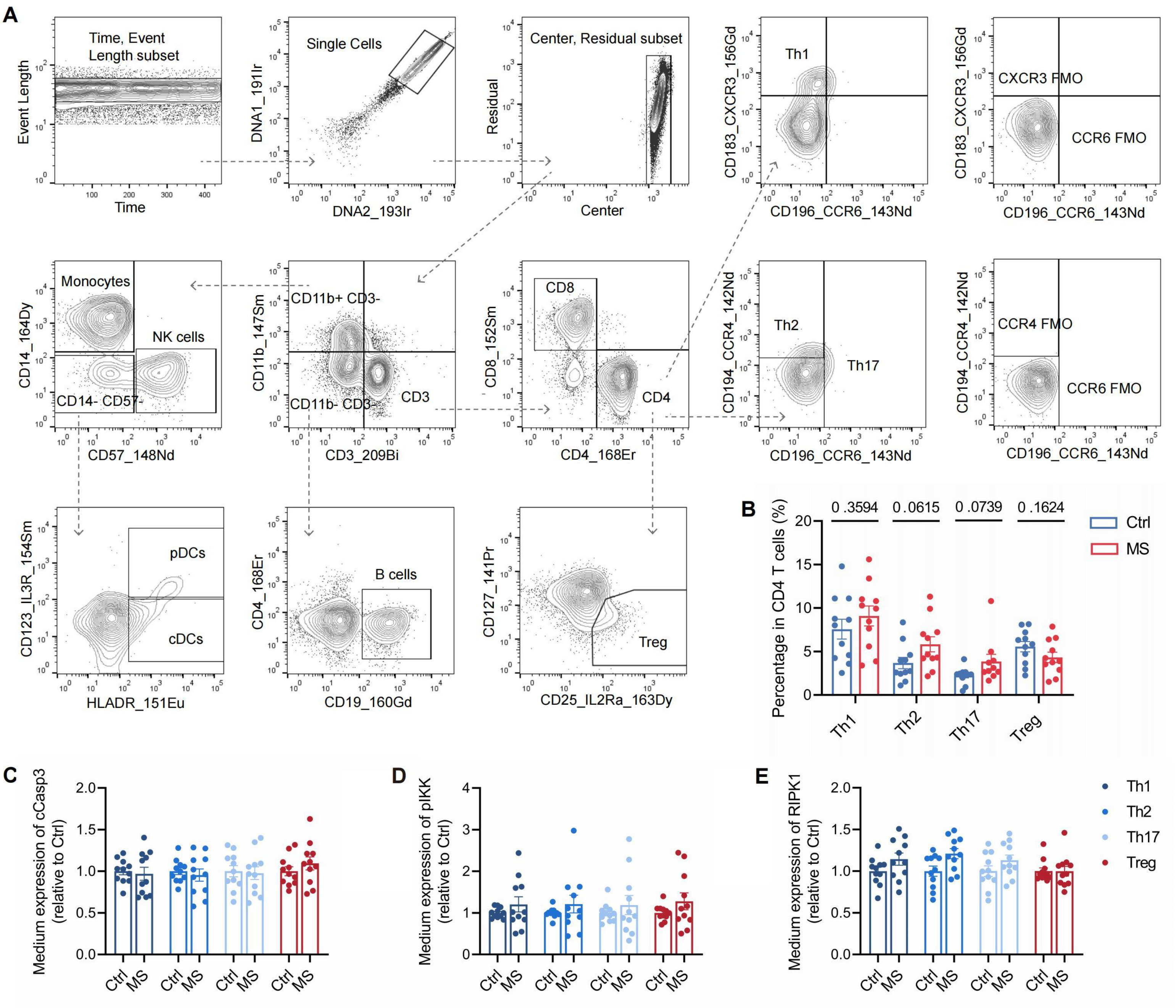

Supplemental Figure 13

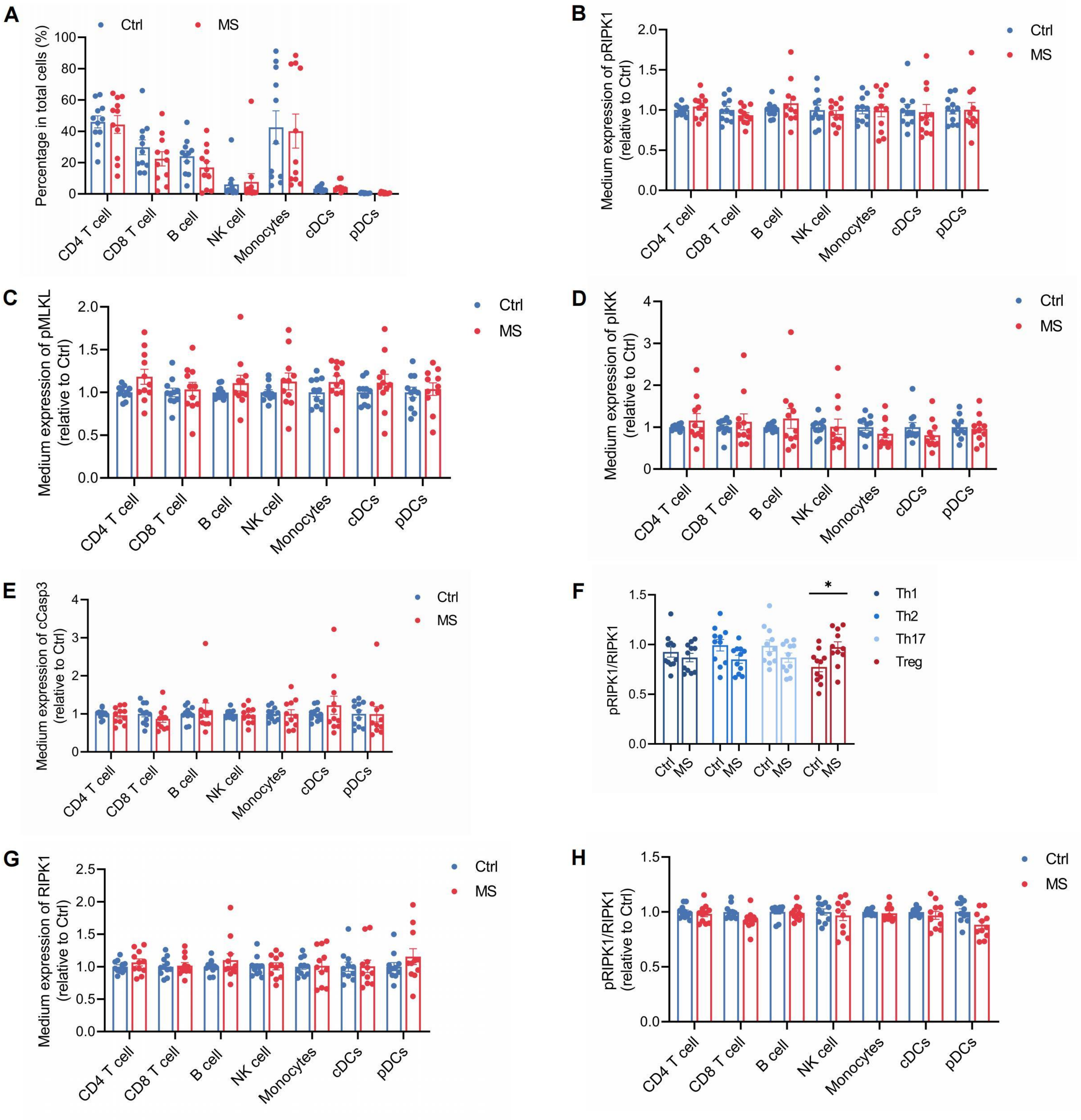

Supplemental Figure 14

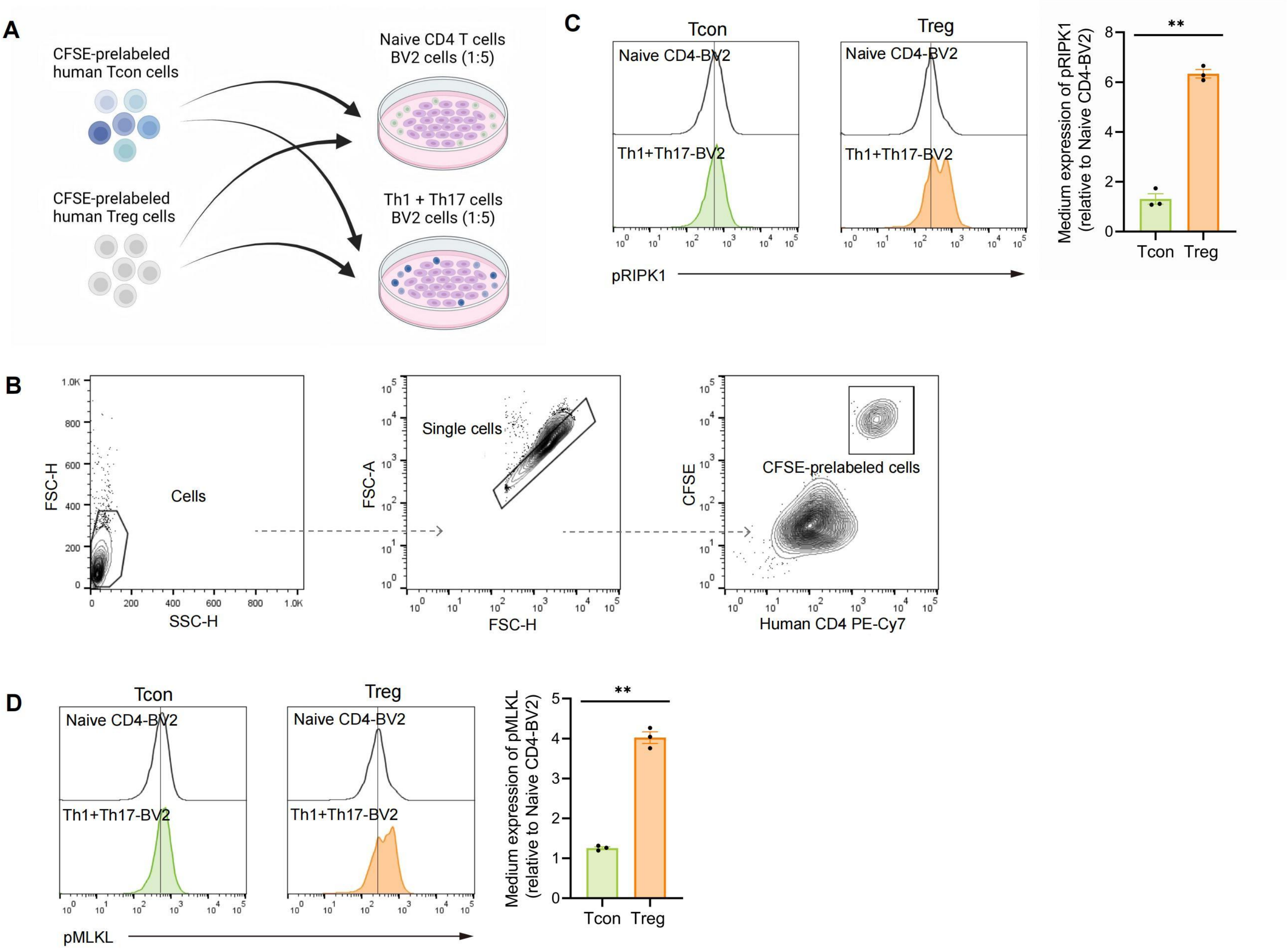

Supplemental Figure 15

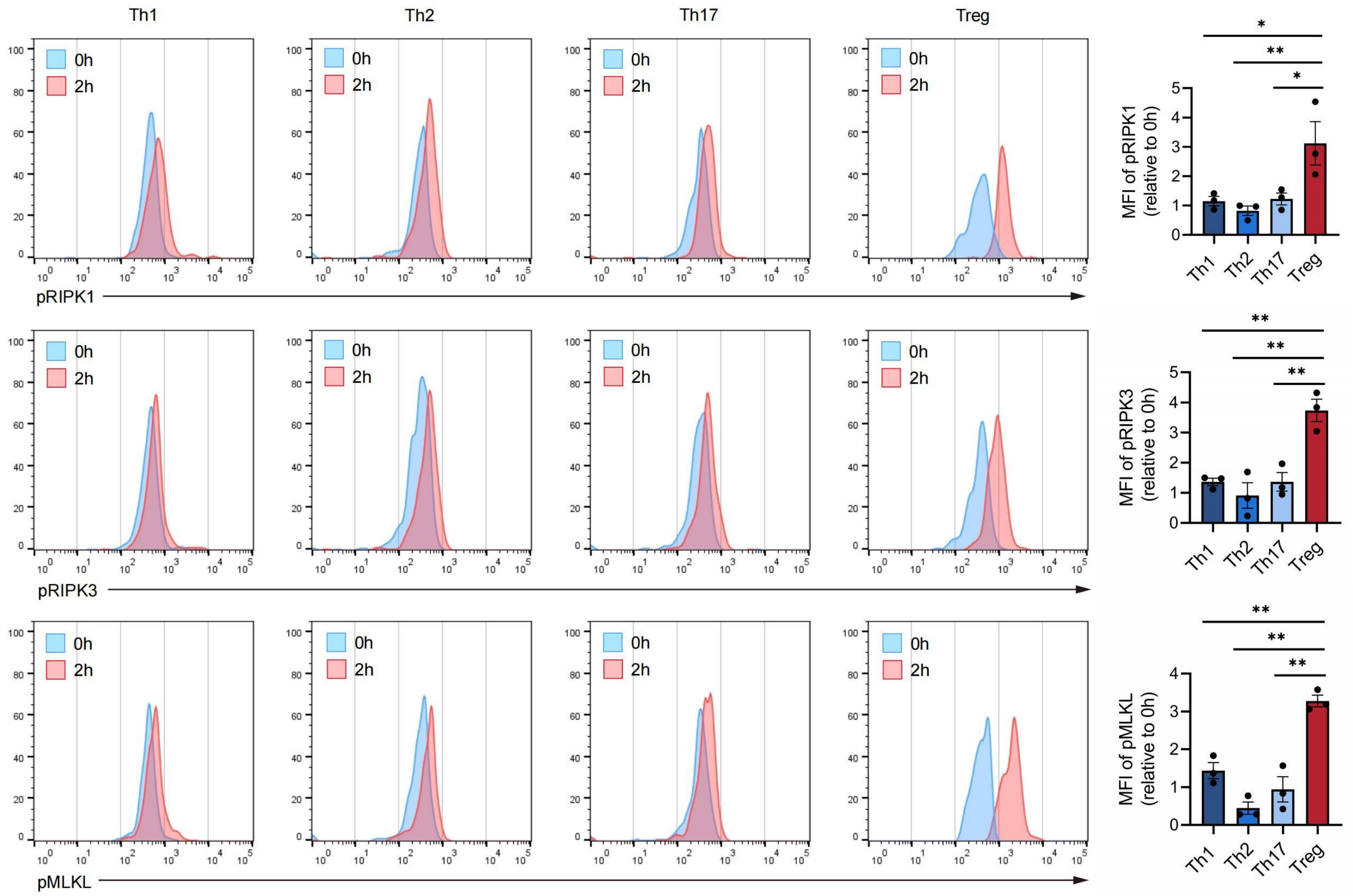

Supplemental Figure 16

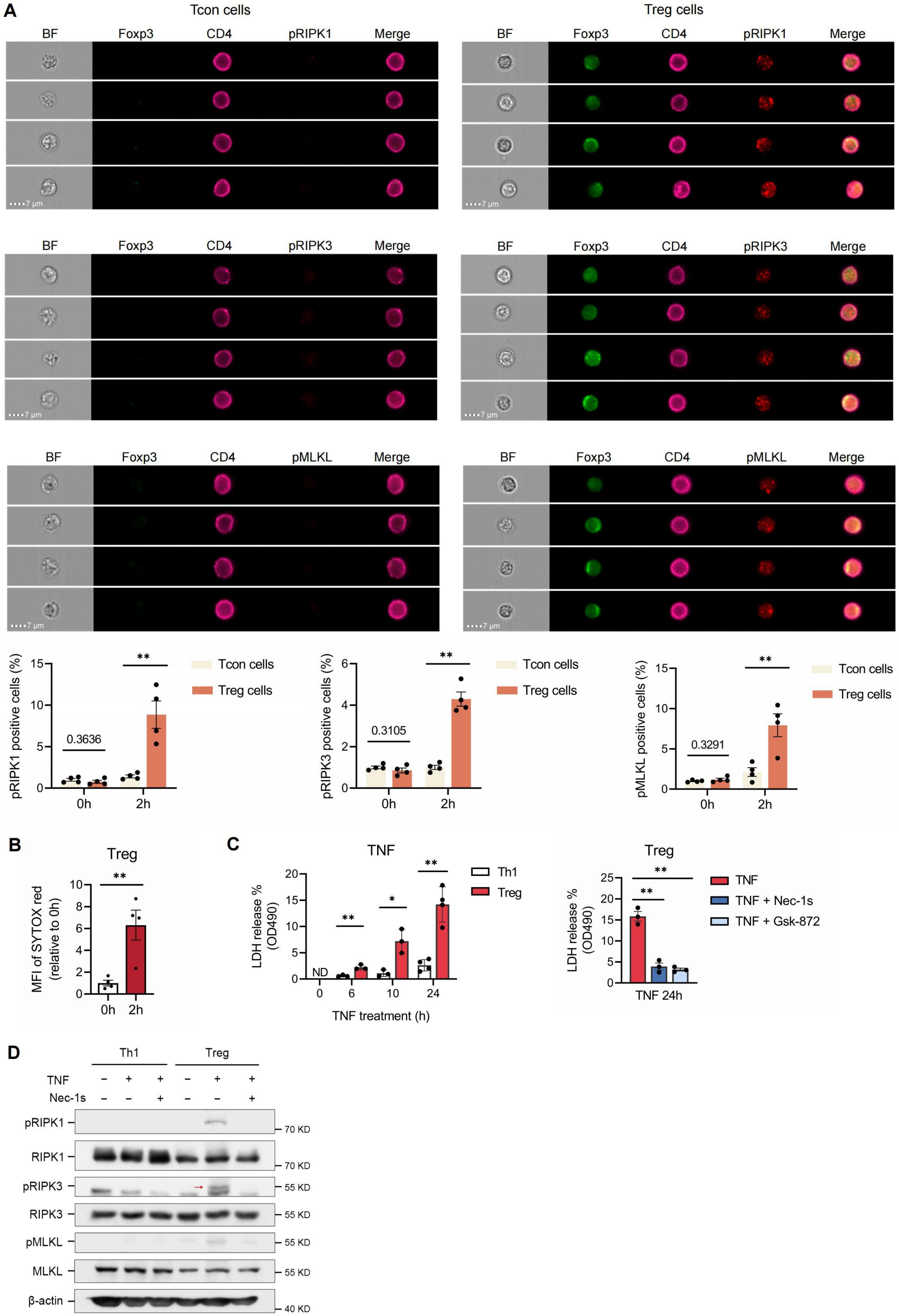

Supplemental Figure 17

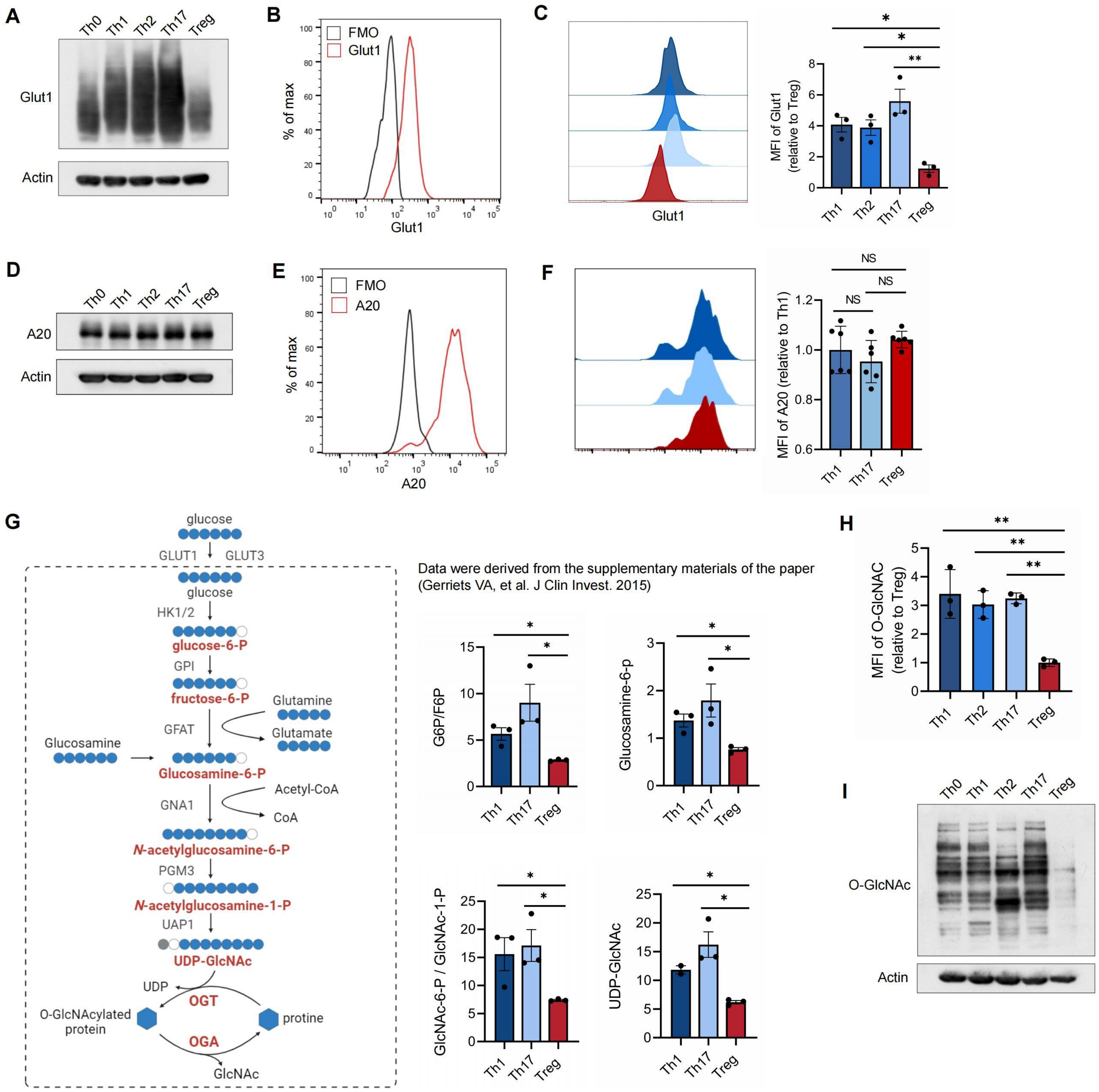

Supplemental Figure 18

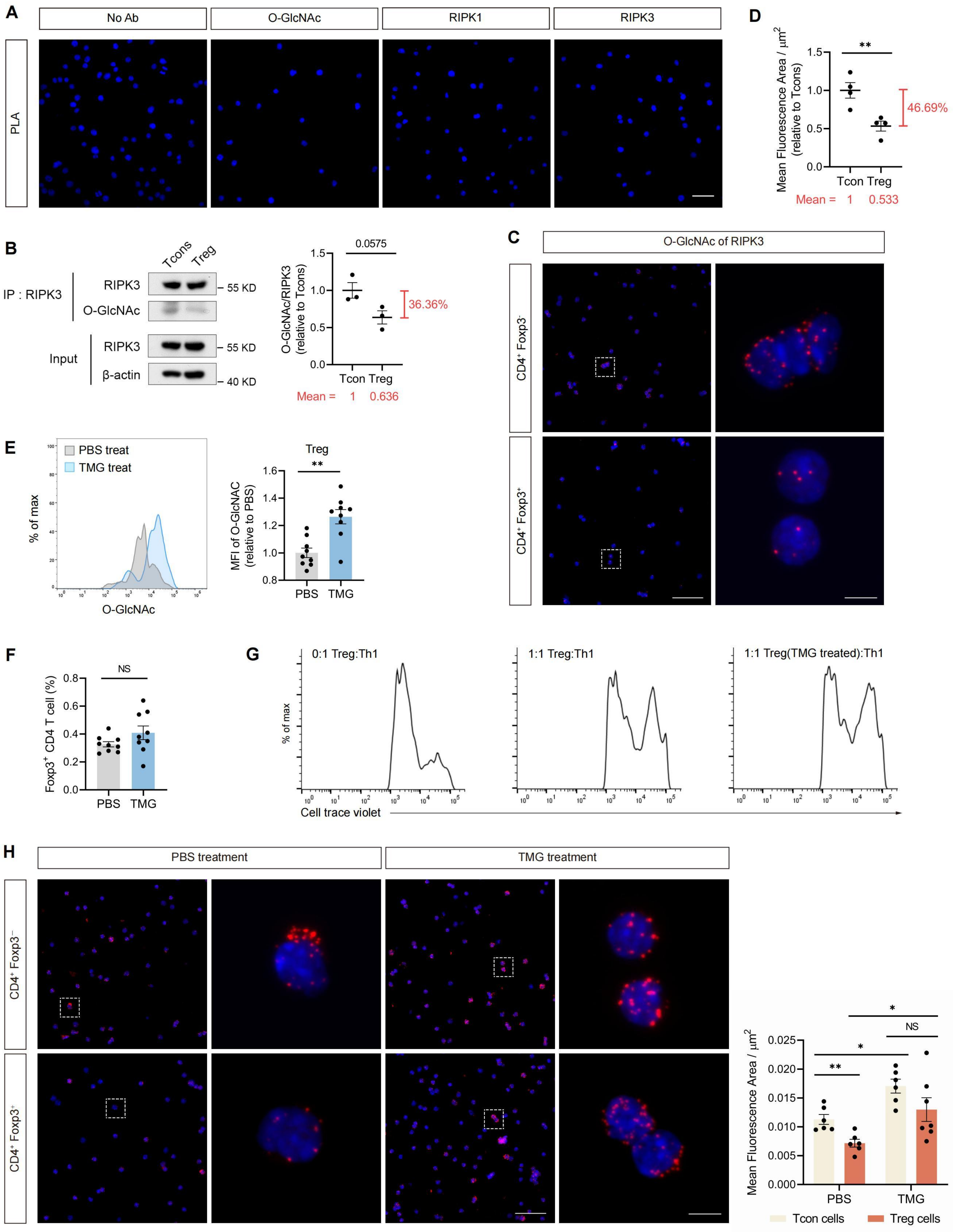

Supplemental Figure 19

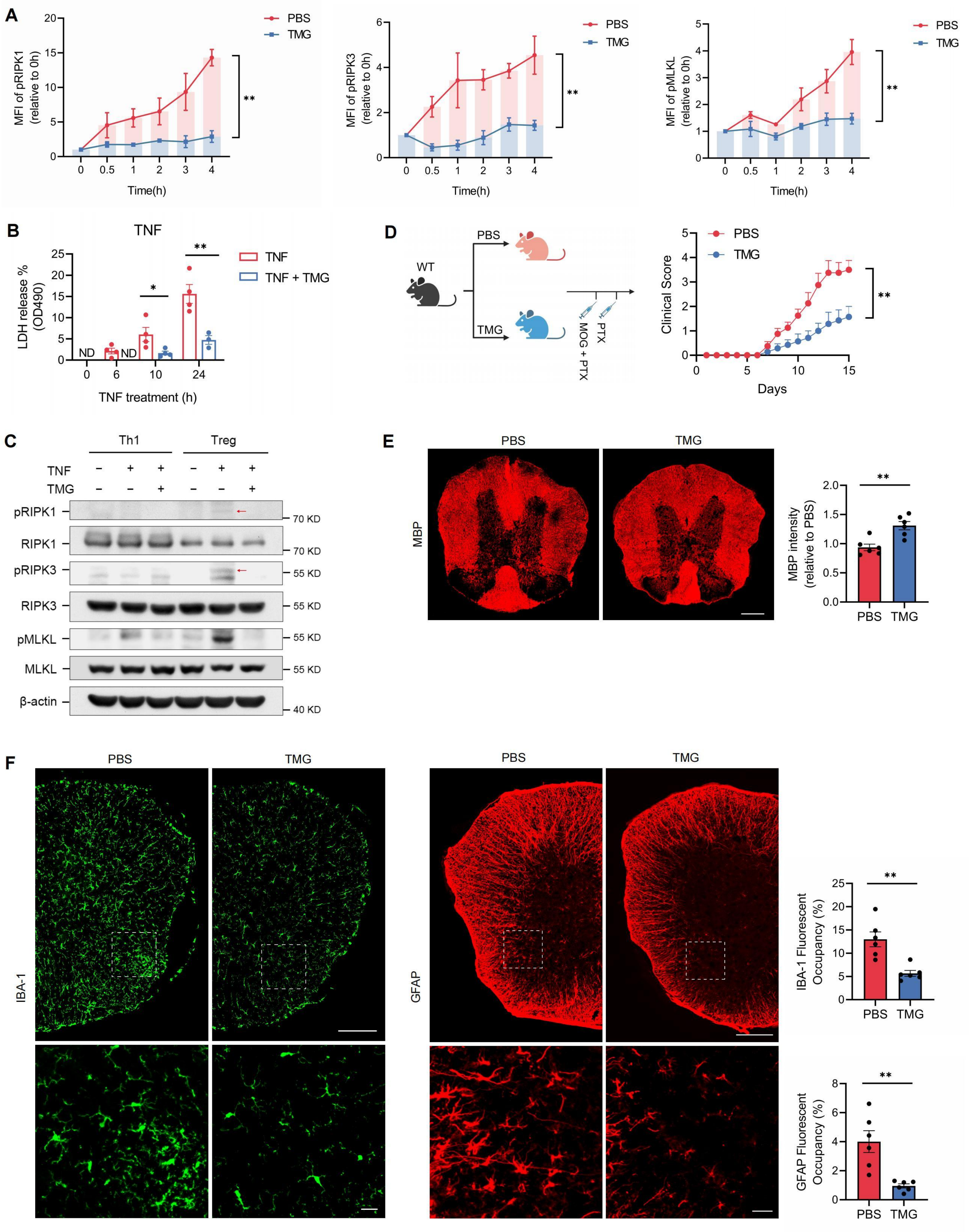

Supplemental Figure 20

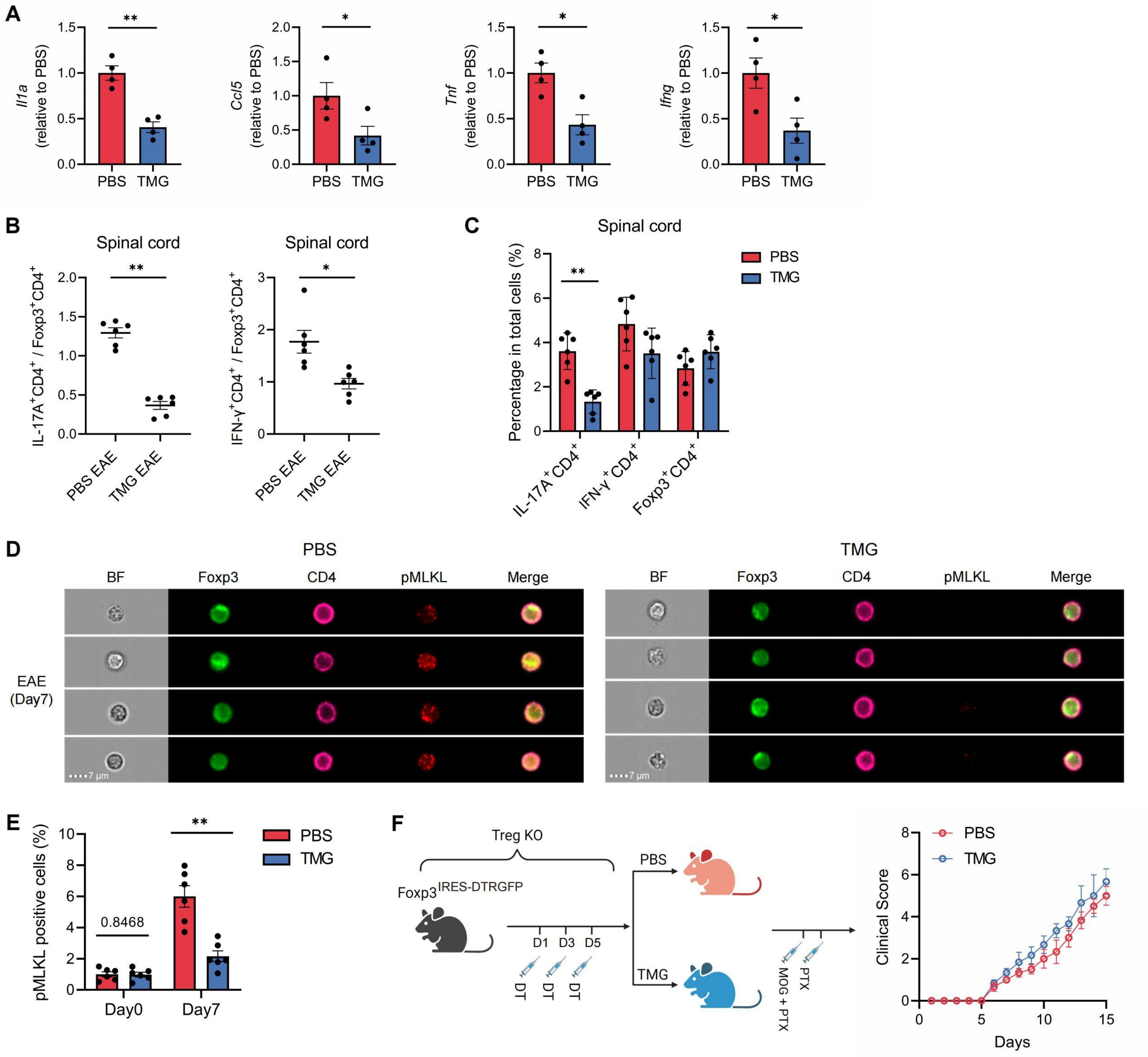

Supplemental Figure 21

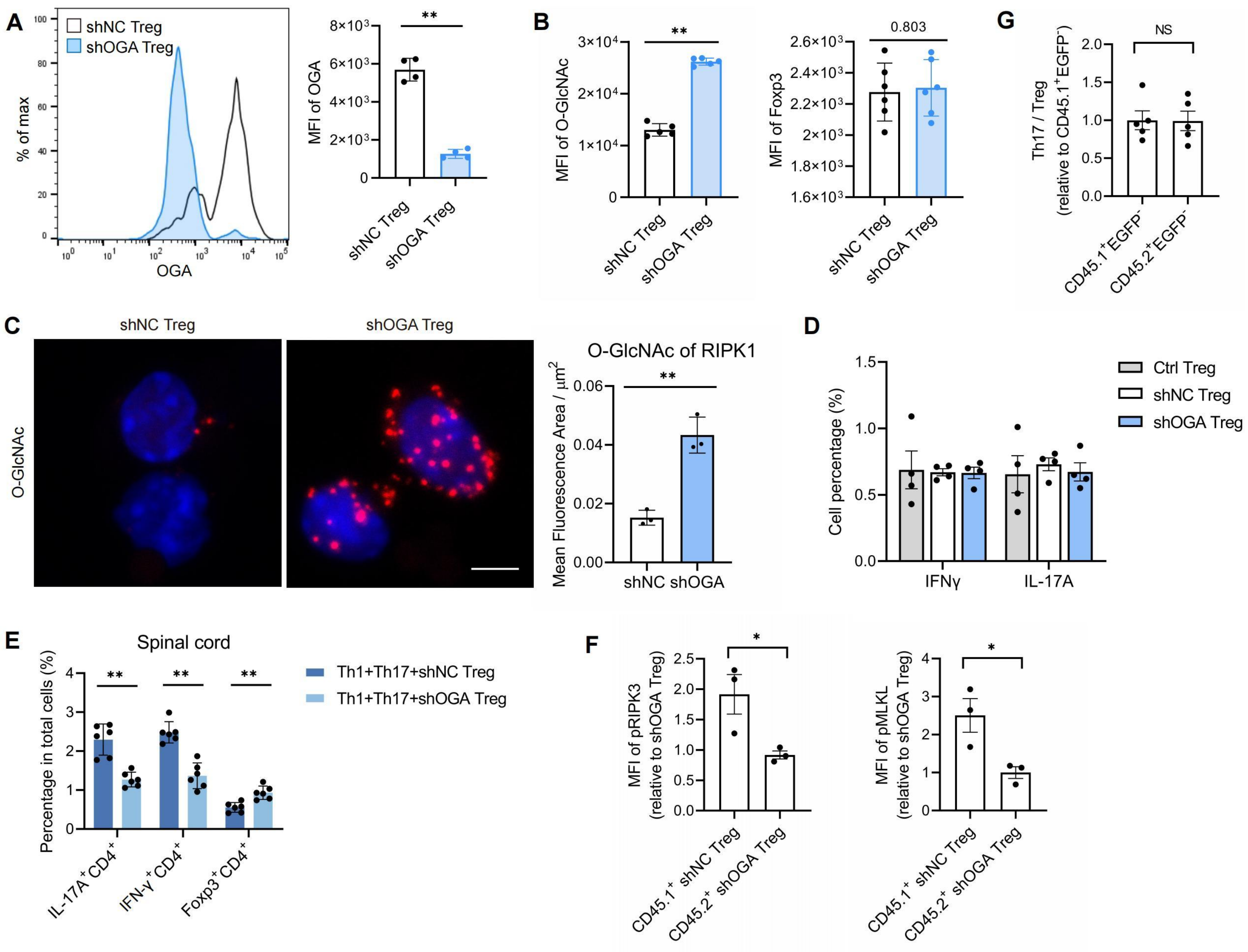

Supplemental Figure 22

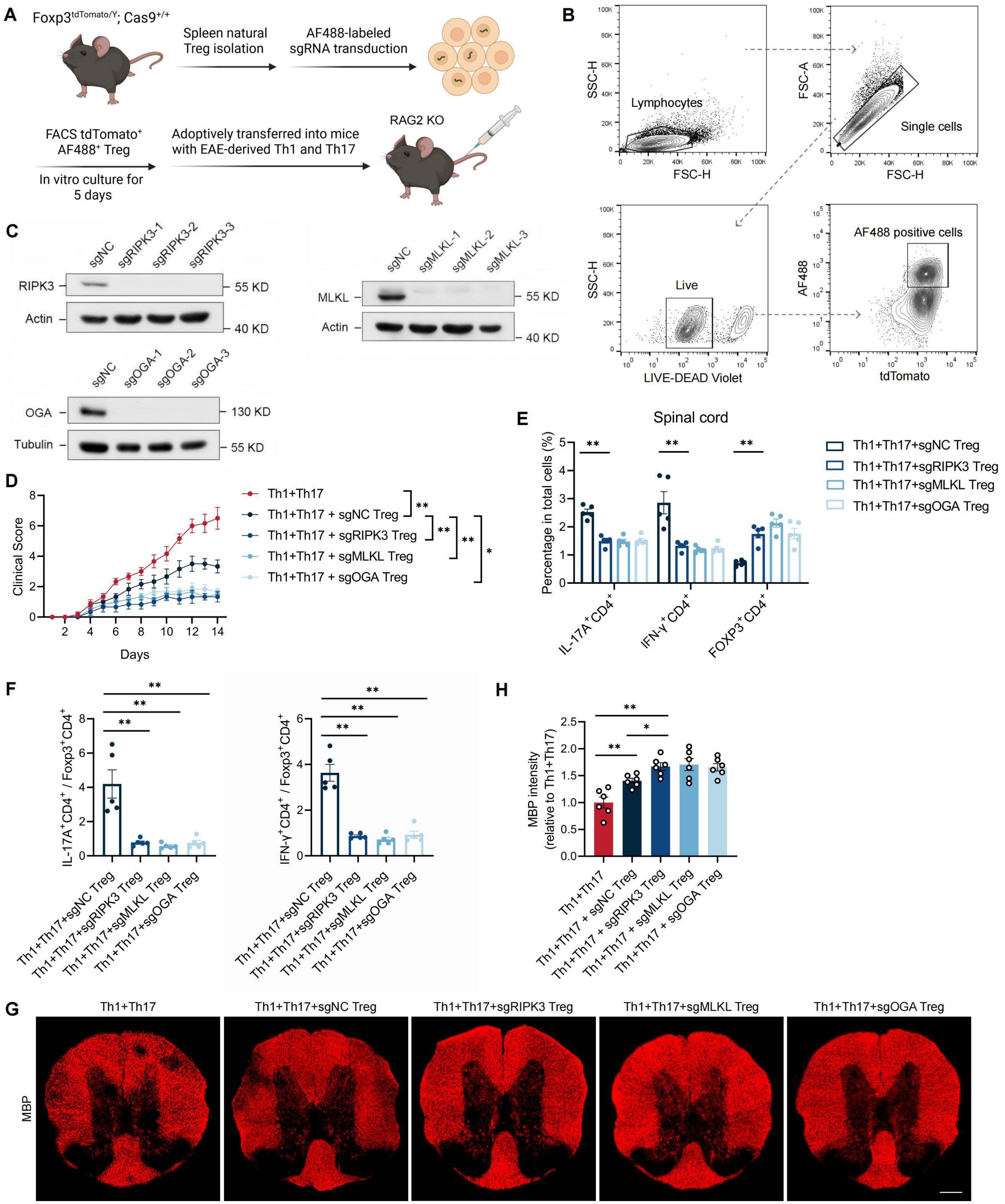

Supplemental Figure 23

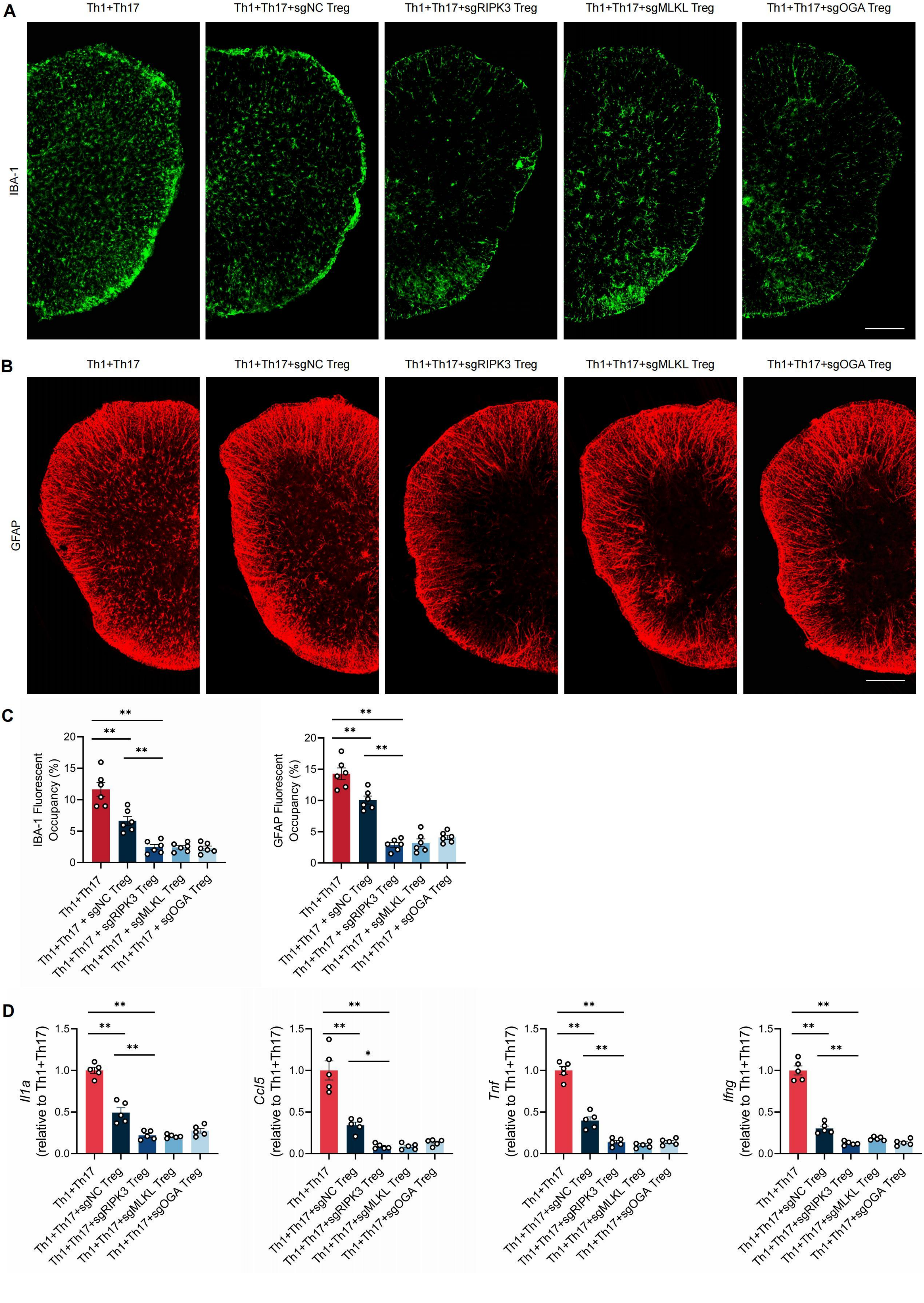

Supplemental Figure 24

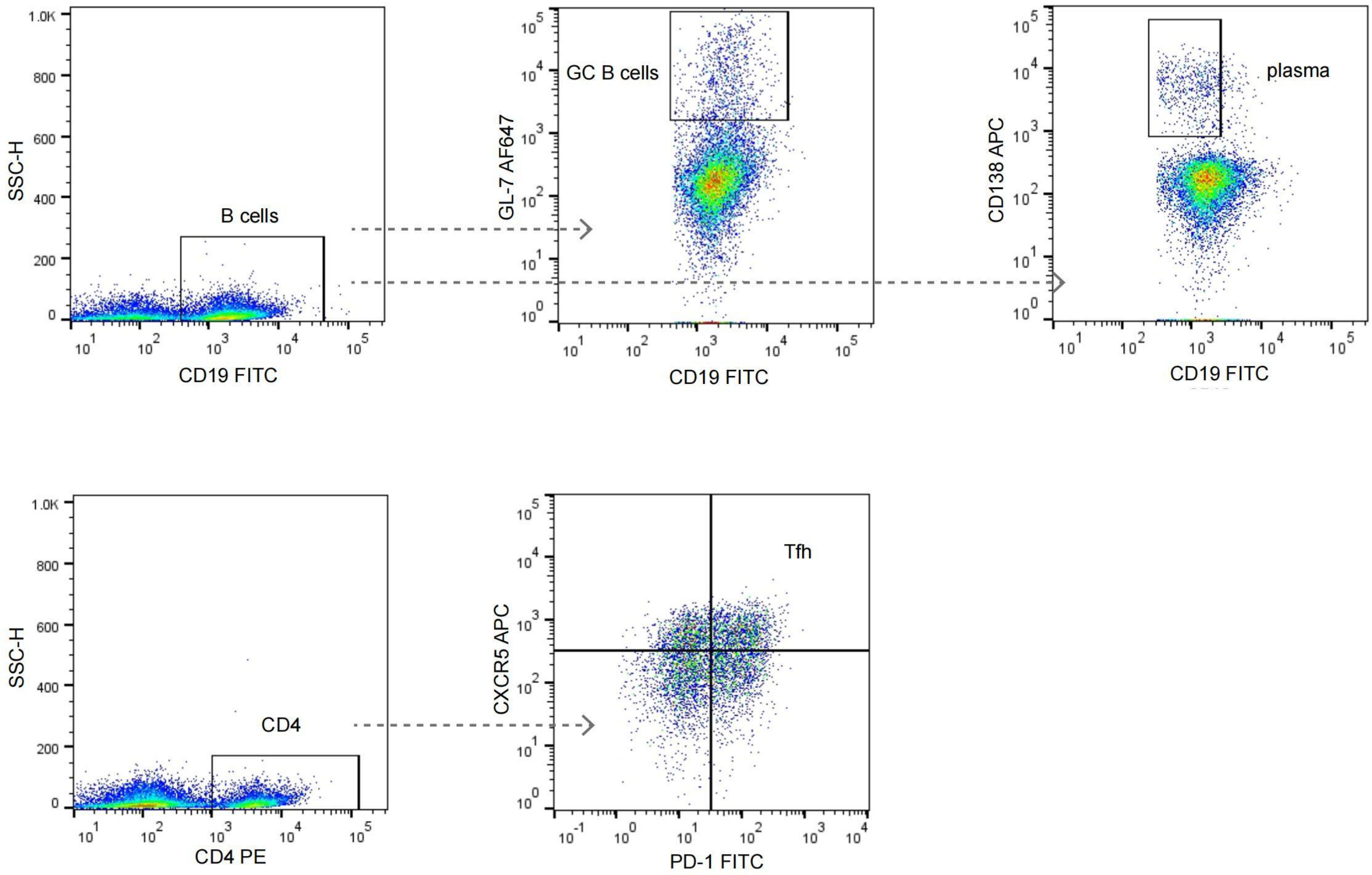

Supplemental Table 1. KEGG pathway analysis of upregulated genes

Pathyway	pvalue	qvalue	Count	Enrichment
Systemic lupus erythematosus	1.59545E-27	4.31612E-25	51	26.79711622
Neutrophil extracellular trap formation	1.2105E-13	1.63737E-11	42	12.91703381
Alcoholism	4.70545E-13	4.24316E-11	41	12.32739903
Inflammatory bowel disease	8.72564E-07	5.90129E-05	15	6.059202937
Influenza A	2.83624E-06	0.000153455	26	5.547257161
Asthma	3.94658E-06	0.000177942	9	5.403779348
Toxoplasmosis	6.49791E-06	0.000242744	19	5.187226614
Viral carcinogenesis	7.17841E-06	0.000242744	30	5.143971775
Hematopoietic cell lineage	1.1952E-05	0.00035926	17	4.922558604
Cytokine-cytokine receptor interaction	2.96889E-05	0.000803163	34	4.527405827
Pertussis	6.56593E-05	0.001614779	14	4.182703706
Th17 cell differentiation	0.000182196	0.004107392	16	3.739462112
Efferocytosis	0.000225957	0.004702092	21	3.645975031
Necroptosis	0.000271479	0.005245866	22	3.566264299
Calcium signaling pathway	0.000353918	0.006382942	28	3.451097335
Complement and coagulation cascades	0.000576364	0.009657153	14	3.23930333
DNA replication	0.00060686	0.009657153	8	3.216911464
Viral protein interaction with cytokine and cytokine receptor	0.000642718	0.009659566	14	3.191979409
Central carbon metabolism in cancer	0.001240995	0.017669567	11	2.906230007
Leishmaniasis	0.001401119	0.018951975	11	2.853525057
IL-17 signaling pathway	0.001801396	0.023160018	13	2.744390903
Tryptophan metabolism	0.001883623	0.023160018	9	2.725006112
TNF signaling pathway	0.001969052	0.023160018	15	2.705742795
Mineral absorption	0.00281268	0.031699741	9	2.550879682
Purine metabolism	0.002929451	0.031699741	16	2.533213795
C-type lectin receptor signaling pathway	0.003212212	0.033422615	14	2.493195748
Glycolysis / Gluconeogenesis	0.003369492	0.033760599	10	2.472435605
Epstein-Barr virus infection	0.00350283	0.033843127	23	2.455580999
Chagas disease	0.004082626	0.038084748	13	2.389060431
Alcoholic liver disease	0.004520447	0.04076333	16	2.344818605
African trypanosomiasis	0.004808535	0.041962428	7	2.317987212
Cell cycle	0.005617211	0.046401339	17	2.250479279
NF-kappa B signaling pathway	0.005660241	0.046401339	13	2.247165063
Transcriptional misregulation in cancer	0.006454863	0.051359129	22	2.190112954

Fanconi anemia pathway	0.006978876	0.052908387	8	2.156214526
Terpenoid backbone biosynthesis	0.007236303	0.052908387	5	2.140483272
Mismatch repair	0.007236303	0.052908387	5	2.140483272
p53 signaling pathway	0.007615132	0.05421299	10	2.118322563
Prion disease	0.008297284	0.056394423	25	2.081064024
Intestinal immune network for IgA production	0.008338475	0.056394423	7	2.07891337
AGE-RAGE signaling pathway in diabetic complications	0.009157211	0.060421133	12	2.038236798
Pancreatic secretion	0.010258014	0.066072922	13	1.988936715
Type I diabetes mellitus	0.010573238	0.066519512	9	1.975792007
Pyrimidine metabolism	0.012067606	0.07419557	8	1.918378866
Starch and sucrose metabolism	0.01272707	0.076511277	6	1.895271553
Small cell lung cancer	0.013702398	0.080583898	11	1.863203418
Lipid and atherosclerosis	0.016020218	0.087644034	20	1.795331587
Legionellosis	0.0161988	0.087644034	8	1.790517153
Allograft rejection	0.0161988	0.087644034	8	1.790517153
Graft-versus-host disease	0.0161988	0.087644034	8	1.790517153
Adipocytokine signaling pathway	0.016543496	0.087753941	9	1.781372713
Apoptosis	0.017151152	0.089227654	14	1.765706698
Nucleotide metabolism	0.017676315	0.090224687	10	1.752608263
Antifolate resistance	0.019486992	0.097624891	5	1.710255195
Rheumatoid arthritis	0.020513701	0.100899927	10	1.687955977
Th1 and Th2 cell differentiation	0.022050815	0.106523675	10	1.65657535
MAPK signaling pathway	0.023630356	0.112151459	25	1.626529734
HIF-1 signaling pathway	0.026807432	0.125036481	12	1.571744783
Fluid shear stress and atherosclerosis	0.034433134	0.157882526	14	1.463023442
Serotonergic synapse	0.037906879	0.170913474	13	1.421281967
Tuberculosis	0.03886172	0.172346195	16	1.410477984
Fructose and mannose metabolism	0.045280734	0.194664724	5	1.34408654
Gap junction	0.04579425	0.194664724	9	1.339189048
Glutathione metabolism	0.046052978	0.194664724	8	1.336742285
Endocrine and other factor-regulated calcium reabsorption	0.048703324	0.199661687	7	1.312441394
Antigen processing and presentation	0.048711237	0.199661687	9	1.312370846

Supplemental Table 2. List of MS-associated microglial signatures

Microglia type	Gene
DAM / MGnD / disease-associated microglia	Apoe, Trem2, Tyrobp, Lpl, Cst7, Clec7a, Itgax, Axl, Gpnmb, Spp1, Lgals3, Cd9, Cd63, Cd68, Csf1, Csf1r, Mertk, Ctsb, Ctsd, Ctsl, Lyz2
Complement / innate immune activation	C1qa, C1qb, C1qc, C3, C4b, Serping1
Chemokines / inflammatory cytokines	Ccl2, Ccl3, Ccl4, Ccl5, Cxcl10, Cxcl16, Il1b, Il18, Tnf, Nfkbia, Nfkbiz, Socs3
Antigen presentation / interferon-responsive myeloid genes	H2-Aa, H2-Ab1, H2-Eb1, Cd74, B2m,Tap1, Tap2, Psmb8, Psmb9, Stat1, Irf7, Ifit1, Ifit2, Ifit3, Isg15, Ifi27l2a
Lipid metabolism / myelin debris handling	Abca1, Abcg1, Fabp5, Npc2, Plin2, Soat1, Lipa, Psap, Acp5
Iron / oxidative stress / lesion-associated stress	Fth1, Ftl1, Hmox1, Cybb, Sod2,
Proliferation-associated reactive microglia	Mki67, Top2a, Cdk1, Ccnb1, Cenpf

Supplemental Table 3. Gene List for shRNA Library

Trp53	Tnfrsf1a	Pou5f1	Casp1	Atm	En1	Cd1d1	Tbx21	Tgfb2
Tnf	Kras	Reln	Ascl1	Eomes	Fyn	Tlr3	Flt1	H2afx
Tlr4	Acta2	Nefm	Gata4	Mapt	Slc2a4	Plp1	Tlr7	Hdac1
Apoe	Hif1a	Gsk3b	Gli3	Il18	Lef1	Emx1	Serpine1	Nodal
Ifng	Myc	Ins2	Tnfrsf1b	Ifngr1	Runx1	Per2	Wnt4	Ubc
Shh	Rag2	Gli1	Foxn1	Hes1	Nr3c1	Drd2	Oprm1	Isl2
Ctnnb1	Foxp3	T	Wnt3a	Nkx2-1	Tbr1	Tgfbr2	Spi1	Arntl
Tgfb1	App	Foxa2	Rbfox3	Trpv1	Csf2	Pitx2	Selp	Il1a
Myd88	Mapk3	Pax3	Ins1	Pvalb	Csf1	Hmgb1	Il7	Ctla4
Il10	Nos3	Psen1	Stat6	Cd44	Neurod1	Neurog2	Abca1	Epha4
Casp3	Esr1	Fasl	Nlrp3	Sst	Stat5a	Bcl2l11	Nr1h3	Ptf1a
Lep	Prkdc	Bcl2	Cnr1	Src	Dll1	Map2	Vdr	Slc6a3
Il6	Il17a	Spp1	Ccr2	Adipoq	Myog	Cd40lg	Cdc42	Ifnb1
Pecam1	Gfap	Tlr9	Hmox1	Ntrk2	Gata3	Mdm2	Socs3	Abl1
Akt1	Sox2	Mapk8	Fgf10	Cxcl12	Jag1	Des	Met	Hnf4a
Pax6	Mmp9	Nos1	Il2rg	Olig2	Erbb2	Lta	Nr5a1	Grin2b
Tlr2	Ptgs2	H2-Ab1	Ret	Ticam1	Bmp7	Dicer1	Adora2a	Cdkn1c
Fas	Cdkn2a	Col1a1	Fgfr1	Cftr	Prox1	Nppa	Nfatc1	Ptpn11
Lepr	Cdkn1a	Stat1	Pdx1	Fn1	Col10a1	E2f1	Pdgfrb	Mef2c
Nfkb1	Prnp	Il2	Cybb	Cdh2	Igf1r	Axin2	Rara	Mt1
Kit	Il1b	Rela	Calb2	C3	Mecp2	Jak2	Prkaa1	Ager
Vegfa	Bdnf	Nes	Ncam1	Htt	Gata1	Fgf4	Cd36	Neurog3
Stat3	B2m	Jun	Smad1	Pik3r1	Myf5	L1cam	Nrp1	Vsx2
Rag1	Igf1	Sirt1	Gad1	Wnt5a	Prkca	Foxg1	Rho	Clock
Bmp4	Lyz2	Pax2	Mbp	Cx3cr1	Mitf	Ikbkb	Gli2	Smo
Nos2	Calb1	Myod1	Pdgfra	Sox10	Vim	Smad4	Gnrh1	Scarb1
Mki67	Kdr	Pcna	Tnfsf11	Ihh	Il12a	Pomc	Itgb2	Gh
Notch1	Bmp2	Cd4	Ptprc	Cd19	Smad2	Npy	Dlx2	Hes5
Cdh1	Egfr	Rac1	Fgf2	Cd28	Fcer1g	P2rx7	Chat	Smad9
Apc	Cdkn1b	Sod1	Cd40	Kitl	Rhoa	Agtr1a	Id2	Cdx2
Il4	Nfe2l2	Ngfr	Fos	Mmp2	Pax7	Calca	Cd80	Dlx5
Pten	Bax	Wnt1	Nkx2-5	Esr2	Trp63	Notch2	Hras	Plat
Tubb3	Th	Msx1	Icam1	Cebpb	Smad5	Snca	Fgfr3	Mif
Dmd	Rb1	Col2a1	Creb1	Prf1	Itgam	Nf1	Gdnf	Pycard
Pparg	Mtor	Fgfr2	Cd8a	Foxo1	Egr2	Bcl2l1	Ntrk1	Cd86
Ldlr	Gja1	Cav1	a	Msx2	Fcgr2b	Insr	Prkcd	Pdcd1
Isl1	Itgb1	Il12b	Il1r1	Lhx1	Il15	Hprt	Dnmt1	Bmpr1a
Sox9	Runx2	Smad3	Tyr	Nkx2-2	Grin1	Rbpj	Nanog	Hand2
Mapk1	Ccl2	Ar	Wt1	Ptk2	Tjp1	Atoh1	Brca1	Il4ra
Fgf8	Ifnar1	Otx2	Igf2	Cxcr4	Cebpa	Btk	Camk2a	Crebbp
Ptch1	Ppara	Ahr	Parp1	Fmr1	Egr1	Krt14	Sod2	Nkx6-1

Mapk14	Ccnd1	Gcg	Ppargc1a	Pde6b	Il13	Itgb3	Foxo3	Sftpc
Cacna1a	Plau	Ctgf	Dnmt3a	Smn1	Timp1	Msr1	Sele	Krt10
Kcnj6	Dkk1	H2-D1	Lrp1	Srf	Hdac2	Xbp1	Cd79a	Tgm2
Lyn	Krt8	Syk	Nr4a2	Bglap	Dll4	Etv5	Htr1a	Yap1
Syp	Myb	Mstn	Slc2a1	Irf1	Dlx1	Dlk1	Ttr	Hspg2
Aicda	Prkaca	Irf3	Aqp4	Ntrk3	Dab1	Erbb4	A1cf	Myh6
Ccnd2	Thra	Edn1	Fabp7	Tap1	Smarca4	Pou4f2	Cd34	Prl
Slc6a4	Ccr7	Ptgs1	En2	Lmna	Tsc2	Fbn1	Cer1	Trpa1
Irs1	Ncf1	Mapk9	Klf4	Lmx1b	Slc17a6	Cyp19a1	Sqstm1	Igf2r
Pou4f1	Scgb1a1	Cd274	Slc1a3	Abcb1a	Itgav	Fgf9	Sftpa1	Eif2ak2
Ppard	Myl2	Ntn1	Ephb2	Ccl3	Nfkbia	Hey1	Alb	Flt3
Hspa5	Tfap2a	Hoxd13	Nefl	Ucp1	Csf1r	Cxcl10	Mt2	Itga5
Apoa1	Nefh	Pik3cg	Prkcz	Hand1	Irf8	Pth1r	Omp	Ikbkg
Egf	Chrna7	Il23a	Sell	Nr1h2	Itga4	Sdc1	Scd1	Smad7
Gria1	Lhx2	Snai1	Ep300	Prkaa2	Sp7	Wnt7a	Hnf1a	Msh2
Hgf	Rarb	Npc1	Il6st	Itga6	Bid	Dag1	Grem1	Slit2
Lif	Cux1	Pdgfb	Sry	Pik3ca	Flt4	Lpl	Cspg4	Tacr1
Ccr5	Dcx	Lama2	Id1	Spry2	Id3	Rps6kb1	Ccne1	Uncx
Efnb2	Gata2	Il33	Myo7a	Raf1	Sema3a	Agtr2	Gnaq	Il22
Tek	Il2ra	Actb	Notch3	Atg5	Cntn2	Aqp1	Gpx1	Grm5
Il7r	Nrg1	Ezh2	Tgfbr1	Wnt3	Thy1	Sycp3	Cd74	Bcl6
Rxra	Tbx5	Pax1	Vwf	Lgr5	Wnt11	Atf4	Ikzf1	Cdk1
Tnc	Thrb	Pthlh	Prkce	Gbx2	Gria2	Ddx4	Prph	Lyst
Bcl11b	Gja5	Grid2	S100b	Lfng	Plg	Vav1	Ren1	Oca2
Cdk4	Stat5b	Afp	Tbx1	Neurog1	Rarg	Ets1	Utrn	Phex
Mmp13	Col1a2	Akt2	Tgfb3	Mycn	Ucp2	Irs2	Dcc	Prkcb
Vcam1	Dlg4	Cxcr2	Bmi1	Abcb1b	Bace1	Lhx3	Ido1	Prkcq
Hspa1b	Epas1	Emx2	Lama1	Pou1f1	Crabp1	Snap25	Il21	Rpe65
Stat4	Lck	Gata6	Six3	Cbl	Braf	Atp2a2	Chrnb2	Gjb1
Tcf3	Ngf	Mnx1	Wnt7b	Cxcr3	Alpl	Atp7a	Angpt1	Mafb
Ghrl	Sp1	Nr1h4	Gad2	Etv1	Hr	Lcn2	Aire	Otx1
Agt	Cdk2	Sparc	Tac1	Amh	Phox2b	Nfatc2	Grb2	Tbx3
H2-K1	Adam17	Srebf1	Nog	Slc11a1	Adora1	Apob	Nr4a1	Vip
Cdk5	Rora	Nod2	Pkd1	Tgfa	F2rl1	Gzmb	Tnfrsf9	Map2k1
Traf6	Uchl1	Cd3e	Map3k7	Ache	Park2	Etv4	Vhl	Actc1
Tyrp1	Casp8	Rorc	Fgf3	Fcgr3	Cnr2	Arrb2	Vangl2	Arnt
Gap43	Hoxb1	Thbs1	Grin2a	Pdpn	Cry1	Alox5	Hhex	Runx3
Per1	Ntf3	Epo	Prdm1	Kl	Ednrb	Crh	Hsf1	Ctsl
Aldh1a2	Adrb2	Epor	Cd14	Ryr2	Ghr	Hoxd11	Ptpn1	S1pr1
Twist1	Bak1	Lgals3	Ddit3	Shc1	Gnas	Ptger2	Sphk1	Psen2
Ptpn6	Cdh5	Myo5a	Il3	Tal1	Gpi1	Atg7	Trpv4	Tagln
Meox2	Socs1	Stk11	Il5	Krt5	Inpp5d	Lyve1	Gusb	Acan
Pgr	Hc	Chuk	Itpr1	Nrp2	Rel	Ryr1	Ccnd3	Neurod6

C5ar1	Trp73	Pkd2	Dcn	Foxp1	Aqp2	Ripk3	Gnat1	Sod3
Cck	Adcyap1	Relb	Ilk	Eln	Bmpr2	Tbx18	Npr1	Tbp
Hey2	Csf3	Cry2	Lama5	Gfra1	Cd24a	Brca2	Otc	Cdh23
Pax5	Foxa1	Efna5	Ascl2	Prph2	Efnb1	Mlh1	Slc8a1	Vamp2
Rock1	Eng	Bbc3	Pdpk1	Tlr5	Fshb	Sin3a	Was	Vegfc
Dnmt3b	F3	Meis1	Tnfsf10	Alox15	Smpd1	Myh10	Adra2a	Map3k5
Lor	Myh7	Nfkb2	Wnt10b	Col4a1	Kmt2a	Ly6a	Fabp4	Ada
Pax9	Cxcl1	Plaur	Ace	Cyp11a1	Hsp90b1	Arg1	Chga	Hsd11b1
Cryaa	Gsc	Itgb4	C1qa	Mpo	Atf3	Ephb4	Foxm1	Ppp1r1b
Eda	Notch4	Ccl5	Lhx5	Pak1	Fgfr4	Itga3	Maf	Ptgds
Gnai2	Icos	Ift88	Muc1	Ppp2ca	Glp1r	Lhx9	Six1	Rbl2
Tert	Fgf7	Casp9	Nox4	Becn1	Jag2	Oxt	Wnt2	Ephb1
Lamc1	Gdf5	Cr2	Postn	Chrna1	Jak3	Sfrp1	Slc17a7	Lefty2
Amelx	Mmp14	Fst	Adam10	Cd247	Nr1i2	Slc12a2	Arx	Rictor
Birc5	Pim1	Sftpb	Ccna2	Eif2ak3	Pik3cd	Xpa	Chrm3	Bmp5
Cnp	Pml	Aldh1a1	Gjb2	Hoxd12	Ezr	Il27	Gal	Capn1
Dbh	Tnnt2	Mag	Hdc	Hsp90ab1	Arrb1	Grm7	Lrp2	Ccr1
Lefty1	Kcnj11	Meox1	Hoxa2	Men1	Cbs	Ctsk	Kif3a	Ctcf
Ebf1	Lamb1	Robo1	Il1rl1	Pgk1	Hfe	Cyp2e1	Krt18	Ctsd
Mc4r	Lhx6	Satb2	Oprd1	Six2	Hoxa10	Drd3	Slc9a1	Cyp1a2
Mcl1	Park7	Traf2	Pou3f1	Tyrobp	Itga2b	Flt3l	Snai2	Eya1
Foxp2	C4b	Trf	Scn8a	Pin1	Krt19	Selplg	Lrrk2	Fgf15
Ltbr	Ctsb	Vcl	Sftpd	Ctnnd1	Tnfrsf4	Sox4	Adrbk1	Hoxa9
Dusp1	Fut4	Vldlr	Tshb	Cdk5r1	Gsk3a	Tdgf1	Pitx1	Htr1b
Cacna1c	Gabra1	Hamp	Mavs	Gjd2	Ptges	Zfp36	Prlr	Lat
Erbb3	Inhba	Lmx1a	Klrk1	Glul	Vcan	Abcg2	Pxn	Lipe
F2r	Lrp6	Irf4	Map3k14	Cfb	Hoxa3	Robo2	S100a9	Polr2a
Pla2g4a	Mmp3	Lgals1	Acta1	Hesx1	Hoxc8	Amhr2	Tfrc	Timp2
Pmp22	Nlrc4	Nid1	Il1rn	Hoxb4	Itpr3	Axin1	Nt5e	Wnt9b
Scnn1a	Angpt2	Pbx1	Junb	Cd47	Lamp1	Cebpd	Vps54	Tardbp
Slc2a2	Nr2f2	Qk	Myo6	Anxa1	Myl7	Elavl4	Agrp	Zbtb16
Syn1	Bmpr1b	Timp3	Rest	Mdk	Prop1	Myh9	Apaf1	Gck
Tcf7l2	Crhr1	Tnni3	Sgk1	Ocln	Ripk1	Pln	Mesp1	Hoxa11
Fgf1	Cryab	Cat	Tpbpa	Prkci	Rock2	Sirpa	Mpz	Hsp90aa1
Itgal	Dct	Kcnma1	Yy1	Hnf1b	Sox17	Star	Ncoa1	Jak1
Ptger4	Grm1	Lhb	Keap1	Irf7	Stx1a	Tnfrsf11a	Pafah1b1	Lrp8
Tnfsf13b	Il17ra	Lrp5	Plcg1	Cyp1a1	Adora2b	Clec7a	Ppy	Zap70
Aqp5	Pdgfa	Pitx3	Tcf4	Ltb	Agrn	Rtn4	Ets2	Avp
Calr	Sfrp2	Bdkrb2	Cacna1d	Rbl1	Cd1d2	Pard3	Ctnna1	Crem
Pax8	Adrb1	Clu	Nr1i3	Syt1	Dbx1	Atp1a1	Onecut1	Eed
Prkcg	Bmp6	Spry1	Fcgr1	Tcf7	Nras	Cd38	Rxrg	Hoxa5
Ppp3ca	Hdac3	Ciita	Tbx2	Nox1	Prkg1	F8	Cxcl2	Hoxd4
Foxc1	Rac2	Meis2	Dst	Mmp7	Hmga2	Nphs1	Tirap	Hspb2

Mmp12	Rad51	Trpc6	Irak1	Bloc1s6	Klf1	Tbk1	Cacna1s	Slc9a3
Pou3f2	Slc1a2	Zp3	Kcnq1	Abcb4	Mc1r	Pik3cb	Fgf5	Anxa2
Vcp	Pink1	Olig1	Myh11	Sox5	Ncoa3	Klf5	Fzd3	Casp4
Agtpbp1	Tubg1	Twist2	Nqo1	Cblb	Kat2b	Casp2	H3f3a	Cd81
Dusp6	Casr	Gzma	S100a4	Socs2	Sh2d1a	Cd9	Lamp2	Cldn5
Nod1	Col18a1	Tnfrsf11b	Zeb1	Emcn	Ddx58	Col4a2	P2ry2	Eif2s1
Abcg1	Ephb3	Pth	Ttn	Tmem173	Acvr1	Csf2rb	Ptger1	Evx1
Bsg	Nr2f1	Nr5a2	Rorb	Adm	Calm1	Cttn	Ripk2	Fgf18
Car2	Fadd	Ace2	Kiss1	Canx	Mecom	Efnb3	Rab3a	G6pdx
Col3a1	Hoxa1	Ehmt2	Txnip	Cfl1	Gnao1	Fabp1	Sema3c	Hrh1
Epha2	Il2rb	Cdh3	Rptor	Cpe	Hspb1	Gabrb3	Slc18a3	Elavl3
Gabrg2	Inha	Gast	Areg	S1pr2	Igfbp2	Lpar1	Ucp3	Lcp2
Gfi1	Ptger3	Hspa8	Csk	Hoxb8	Flna	Itgb7	Zic2	Npy2r
Rxrb	Slit1	Igfbp3	E2f3	Lbx1	S100a8	Lox	Map3k8	Peg3
Myocd	Wnt2b	Il15ra	Foxj1	Rnf2	Ghsr	Mapkapk2	Dysf	Prrx1
Ube3a	Foxl2	Sumo1	Jup	Stim1	Tph1	Myl1	Eif2ak4	Tbx4
Nr0b2	Opn4	Plcg2	Maoa	Tnfaip3	Retn	Npy1r	Atoh7	Thbs2
Tslp	Ern1	Ceacam1	Ndn	Wnt6	Cd8b1	Prom1	Cdk6	Plin2
Pnpla2	Adrb3	Map2k2	Numb	Skp2	Col4a3	Stmn2	Ednra	Diap1
Nr3c2	Atf2	Bad	Cx3cl1	Xrcc6	Cyp17a1	Slc2a3	Efna2	Dlg1
Chrna4	Crx	Fcer1a	Sim1	Foxd1	Dlx3	Sox11	Mfn2	Dmp1
Apoa2	Foxc2	Idh1	Aifm1	Nedd4	Dpp4	Tbxa2r	Mdm4	Epha3
Hcrt	Hdac6	Nrl	Bcr	Pcsk1	Map1b	Slc18a2	Ptafr	Hspd1
Hoxa13	Hbegf	Rax	Prdx6	Prkar1a	Nppb	Wnt8b	Slc12a3	Ldha
Krt1	Htr2c	Ncor1	Xiap	Ptn	Pla2g2a	Fbxw7	Syn2	Enpp1
Nfatc3	Ibsp	Srebf2	Ccr6	Tie1	Mst1r	Sost	Tet2	Plk1
Sox1	Itga2	Alx4	Ckm	Tln1	Tbx6	Ntf5	Tlr6	Pou3f4
Hdac4	Me1	Bgn	Col6a1	Zic1	Chrm2	Nr0b1	Tnfrsf18	Ppia
Slc32a1	Mpl	Cav3	Hcn1	Panx1	Mafa	Gadd45a	Cyp26b1	Ano1
Bcl2l12	Myf6	Cfh	Hoxb5	Cbfb	Elane	Eif4ebp1	Mapk7	Egln1
Cga	Oprk1	Dvl2	Irx3	Cd22	Il21r	Fasn	Apln	Asic1
Htr2a	Sdc4	Igfbp5	Mybpc3	Cflar	Rab27a	Gja4	Cdh6	Cbx5
Il9	Trp53bp1	Ncoa2	Pou2f1	Epha7	Cdkn2b	Hbb-y	H2-Aa	Cdx1
Itgax	Gabbr1	Pck1	Hspa1a	Fli1	Dyrk1a	Itga7	Hbb-bh1	Csf3r
Itk	Chrm1	Ptk2b	Scn5a	Gjc1	Eif4e	Nkx3-1	Pgf	Dazl
Kcna1	Dvl1	Trim28	Ticam2	Scx	Hck	Prm1	Plxna2	Ephx2
Klf2	Hoxd10	Dnmt3l	Xrcc5	Sox6	Mos	Slc4a1	Atxn1	Lhx8
Mertk	Elavl1	Cd5	Cdkn2c	Cacng2	Rps6	Mkl1	Thbd	Mesp2
Nf2	Lmo4	Cux2	Crhr2	Faah	Shox2	Axl	Tshr	Tfam
Ogg1	Mef2a	Dhh	Galc	Gba	Traf3	Atp1a2	Slc40a1	Tuba1a
Pcsk2	Mfge8	Dll3	Grn	Gsx2	E2f2	Rap1a	Cadm1	Vil1
Gla	Polb	Vasp	Cyp27b1	Ncl	Gdf9	Dync1h1	Arc	Large
Bdkrb1	Med1	Sphk2	Dmc1	P2ry1	Gjb6	Fosb	Arf6	Mycl

Lpin1	Pmaip1	Acvr2a	Dnm1	Pkm	Klrb1c	H2-DMa	Cbx1	Ppargc1b
Hdac5	Dnm1l	Il6ra	Inhbb	Rgs2	Sigmar1	Hoxb3	Erg	Mlph
Cyr61	Tas1r3	Abcc1	F11r	Nr2e1	Osm	Lipc	Fga	Mc3r
Jund	Itga1	Pcnt	Lmnb1	Txn1	Pld1	Fabp5	Fhl2	Myl4
Stmn1	Aplp2	Ptpra	Mest	Tet1	Tph2	Mas1	B4galnt1	Ptx3
Rasgrf1	C3ar1	Tcf12	Mttp	Suz12	Tg	Slc12a1	Gnb1	Rab7
Tcp1	Cdh11	Thpo	Nfe2	Cxcl13	Tpm1	Ncor2	Id4	Ryr3
Trpc1	Ctss	Tnfsf12	Sema3f	Nampt	Nr2c2	Tnfsf9	Igfbp4	Sag
Atr	Fgf17	Tnfsf4	Sptbn1	Map1lc3a	Vav2	Atf6	Lama4	Slit3
Crtc2	Gabra6	Wnt5b	Stxbp1	Aif1	Atrx	Zic3	Mog	Snta1
Sall4	Kcnd2	Ywhaz	Ybx1	Aqp3	Zfpm2	Akt3	Nck1	Sox18
Cpa1	Ltb4r1	Esrra	Icosl	Btg2	Fus	Map2k3	Rhox5	Sp3
Chek1	Havcr2	Camk2d	Foxo4	Cacna1b	Hcn4	Trpm5	Scnn1g	Tubb4a
Edar	Myh2	Orai1	Sall1	Entpd1	Rcan1	Carm1	Frzb	Ung
Grik1	Pou3f3	Chrng	Golga2	Cdh8	Abca4	Glo1	Slc22a5	Mapk10
Hexb	Ren2	Camk4	E2f4	Camp	Dab2	Cbx3	Sort1	Lig4
Mef2d	Spta1	Cd69	Glb1	Ces1c	Fancc	Ccna1	Suv39h1	Stk4
Slc11a2	Stra8	Bcar1	Cxcr5	Gna13	Gnb2l1	Cln3	Usf1	Arl13b
Scn10a	Cd27	Cyp1b1	Casp7	Pdia3	Itch	Gabra2	Vtn	Lin28a
Sptan1	Gsn	Cyp26a1	Chrd	Hoxb7	Kcnn4	Htr2b	Sufu	Dsp
Trpc3	Disc1	Dgat1	Dlx6	Hoxc6	Lamb2	Itgae	Homer1	Adh1
Spry4	Cd207	Gcm1	Fzd8	Hoxd9	Ly96	Kcnj10	Spo11	Alox12
Zeb2	Nbn	Gna11	Ghrh	Htr3a	Mip	Smad6	Tnfsf14	Rhob
Map3k1	Aldh1a3	Lhcgr	Kcnj2	Limk1	Mllt4	Mbl1	Gria3	Ccr4
Opa1	Slc12a5	Cxcl9	Cited2	Plcb1	Mme	Spn	Tyk2	Ackr3
Kdm1a	Tbx20	Mx1	Scnn1b	Psap	Msi1	Aurkb	Ncstn	Comt
Adora3	Ifih1	Musk	Slc34a1	Tle4	Enpp2	Bhlhe40	Smarca2	S1pr3
Phox2a	Aldh2	Pdyn	Abcc8	Trh	Stat2	Tyro3	Wasl	Hba-a1
Cldn1	Amy1	Rbp1	Umod	Vdac1	Aurka	Rapgef3	Nfatc4	Hcn2
Fgr	Apex1	Sstr2	Slc26a4	Map2k4	Sycp1	Chek2	Adra1b	Cd200
Ghrhr	Cckbr	Ywhae	Esrrb	Il27ra	Tcf21	Pla2g6	Bnip3	Neu1
Grb10	Crk	Slc9a3r1	Nrip1	Ppif	Nr1d1	Crlf2	Cacnb4	Nfia
Hoxa7	Cd55	Nfat5	Adcy1	Mylk	Ago2	Mlxipl	Cd59a	Plxna1
Hoxb6	Hoxa4	Cacna1h	Stub1	Acp5	Ror2	Pgm1	Csnk2a2	Tfap2c
Il12rb2	Lmo2	Dock7	Angptl4	Actn2	Ppp2r1a	Rps6ka5	Cyp7a1	Ube2i
Itpr2	Trpm8	Ap3b1	Adipor1	Acvrl1	Rtn4r	Dtnbp1	Efna1	Vegfb
Ldb1	Pah	Apobec1	Pcsk9	Dnm2	Neurod4	Fgg	Foxf1	Xpc
Blnk	Furin	Aprt	Dhfr	Epha5	Bcl10	H6pd	Hoxb9	P2rx3
Penk	Ccl11	Atp2b2	Grik2	Fcgrt	Cd2ap	Plekha5	Il18r1	Trpm2
Pltp	Tcf7l1	Cited1	Ivl	Fzd6	Ctbp2	Acvr2b	Jarid2	Tnfrsf13c
Cyld	Il17f	Dio3	P2rx4	Ngb	H2-Eb1	Opn1sw	Unc5b	Nfix
Cth	Esrrg	Abl2	Pnp	Cltc	Hk1	Cp	Ogt	Cd244
Bsn	Gtf2ird1	Kiss1r	Ptgir	Hes7	Ndst1	Dio2	Egln3	Nrxn1

Eef1a1	Gpnmb	Adra2c	Serpinf1	Wwtr1	Iapp	Drd5	Cacna1e	P4hb
Elk1	Dgcr8	Barx1	Csnk1e	Rps6ka3	Kcnc1	Efna3	Capn3	Per3
Fcer2a	Actn1	Capn2	Frs2	Alcam	Lum	F2rl3	Cask	Ppp3r1
Fshr	Akr1b3	Cr1l	Batf3	Ank3	Asic3	Gcgr	Coro1a	Scn1a
G6pc	Arg2	Cycs	Dkk3	Bach1	Bhlha15	Glra1	Dpysl2	
Hmox2	Cacna1g	Ext1	Fezf2	Bcl3	Ccl4	Gna12	Crp	
Hpse	Ercc1	F9	Abcg8	Camk2b	Sema3e	Hspa9	Ddx5	
Igfbp1	Gip	Gfra2	Adipor2	Clcn1	Tfeb	Il12rb1	Gabra3	
Lifr	Slc6a9	Gstp1	Prdm16	Cldn3	Kdm6b	Lcat	Hoxc9	
Psmb8	Gnrhr	Lamc2	Apobec3	Drd4	Tk1	Epcam	Ldhc	
Nr4a3	Marcks	Psmb9	Tubg2	Fkbp5	Trpc4	Msh6	Gadd45b	
Polg	Mbl2	Slc3a2	Lgr4	Nr6a1	Osr1	Mthfr	Cldn11	
Pon1	Prdx1	Mrc1	Olr1	Nkx6-2	Ercc6	Pms2	Oxtr	
Sos1	Pax4	Sox8	Bub1b	Sdc2	Pias1	S100a6	Pou2f2	
Prdx2	Prkd1	Irak4	Ctnna2	Il11	Dspp	Apcs	Soat1	
Tiam1	Plec	Tlx3	Cntn1	Klra8	Wls	Smarcb1	Tead1	
Heyl	Ppp3cb	Rab5a	Col9a1	Lgals9	Hoxb2	Irf5	Tgfb1i1	
Gper1	Ptch2	Sfn	Cxadr	Msn	Ank1	Grm2	Pde4d	
Adcy3	Rspo1	Adar	Cyp21a1	Nfya	Cckar	Nkx3-2	Ywhab	
Adamts1	Ptpn2	Plin1	Edn3	Khdrbs1	Chrm4	Cldn2	Ikbke	
Adcyap1r1	Grhl3	Agtrap	Fah	Pyy	Cys1	Ctbp1	Pdzk1	
Birc3	Slc6a1	Mta1	Gpd1	Tle1	Egr3	Dclk1	Map1lc3b	
Btrc	Cln8	Tspo	Gja8	Tlr1	Enah	F2	Plxnd1	
Daxx	Pvrl1	Cldn4	Foxd3	Tlx1	Etv6	Gab1	Atxn3	
Fes	Xpo1	Cst3	Hoxd3	Tub	Fabp3	Hipk2	Atp1b1	
Fth1	Chd4	Fgf6	Ksr1	Pcgf2	Gaa	Hk2	Pcdh15	
Fzd4	Gabra5	Gabrd	Mbd2	Trpm7	Hhip	Myh1	Ddr1	
B4galt1	Actg1	Gas6	Mmp8	Fbxo32	Pick1	Pbx3	Cdkn2d	
Muc5ac	Grin2c	Gnai3	Por	Itga9	Pou4f3	Pld2	Clcn3	
Neo1	Kcnk2	Has2	Slc1a1	Lmo1	Rdx	Nlgn1	Ccr9	
Pknox1	Oprl1	Hmgcr	Unc5c	Ank2	Dntt	Ptprz1	Crkl	
Tmsb4x	Vipr2	Hp	Wnt10a	Aco1	Top2a	Sepp1	Ddc	
Robo3	Abcg5	Itgb5	Plagl1	Anxa5	Yes1	Serpine2	Dsg3	
Rbp4	Iqgap1	Kif5b	Eif2a	Apoa4	Zfx	Adcy5	Gas1	
Smarcc1	Brd4	Klk1	Pttg1	Cyp2a5	Trib3	Gp6	Opn1mw	
Sox3	Pdcd1lg2	Kpna2	Aim2	Figf	Sirt6	Gphn	Kpnb1	
Tfap2b	Bhlhe22	Krt17	Trim63	Fosl1	Actn4	Piwil2	Crb1	
Rasa1	Dppa3	Myh4	Vav3	Fzd7	Scrib	Hfe2	Madcam1	
Ulk1	Lrat	Nfib	Sv2a	Grpr	Birc2	Trem2	Mtf1	

Supplemental Table 4. Downregulated and upregulated regulators identified by shRNA screening

Downregulated regulators	mean_LFC	p_value	Upregulated regulators	mean_LFC	p_value
Tnni3	-1.065530423	0.000418924	*Ripk3*	0.964172071	0.028989596
Hfe2	-1.000760749	0.02531474	*Gabrg2*	0.906475609	0.042919325
Limk1	-0.984619089	0.011081839	*Ifng*	0.839102852	0.04973584
Tbxa2r	-0.886230874	0.027671199	*Insr*	0.813973354	0.0415368
Hcn1	-0.875473332	0.020965736	*Cacnb4*	0.809239799	0.011674579
Efna2	-0.868737104	0.01611121	*Sox17*	0.808310668	0.003797644
Dlx5	-0.835870937	0.018199376	*Axl*	0.77984161	0.011272026
Mta1	-0.804911292	0.03741399	*Hdac1*	0.753579671	0.032865092
Timp1	-0.788430135	0.020551841	*Tgm2*	0.726000024	0.029425011
Tgfbr2	-0.778565403	0.024426553	*Xpo1*	0.723182899	0.049262316
Ckm	-0.768531932	0.016841353	*Slit1*	0.710422476	0.015342664
Il10	-0.764745968	0.027842226	*Cldn3*	0.654621415	0.038274545
H2afx	-0.762176222	0.034514338	*Nefh*	0.649185698	0.009354299
Mafb	-0.754889371	0.023247591	*Tubb4a*	0.64823853	0.015458106
Jup	-0.753829527	0.042239836	*Cxcr2*	0.648035708	0.033886797
Pxn	-0.743870158	0.020834328	*Becn1*	0.645914345	0.012470745
Esrrb	-0.719352061	0.012343033	*Ptger4*	0.640782717	0.013773309
Ppp3cb	-0.699479188	0.037194071	*Pgm1*	0.634674385	0.035022011
Vcl	-0.696437951	0.010907928	*Slit2*	0.61963761	0.044115246
Wnt1	-0.696005061	0.004116737	*Irs1*	0.619121008	0.039384289
Gabbr1	-0.670099629	0.048761783	*Krt8*	0.600694494	0.042313922
Pla2g2a	-0.668274077	0.011550387	*Mpo*	0.576717832	0.025879773
Fut4	-0.667593167	0.048102598	*Nox4*	0.567239529	0.004295058
Sfrp2	-0.657397527	0.02781832	*Ccr5*	0.565268853	0.030590521
Ncoa3	-0.630883059	0.023717993	*Smad1*	0.559722155	0.020491591
Vsx2	-0.626026064	0.038098234	*Ager*	0.545715689	0.00225524
Emx2	-0.625851058	0.013834982	*Slc12a2*	0.543340658	0.03419596
Lhx5	-0.621354176	0.021675914	*Apob*	0.539013928	0.047600236
Syk	-0.614632641	0.005772675	*Bmpr1a*	0.518822718	0.006351281
Ascl2	-0.614451641	0.010416603			
Avp	-0.608690694	0.025440742			
Ctla4	-0.589386706	0.045154302			
Sox1	-0.573438095	0.024112989			
Eln	-0.566429765	0.017258175			
Xiap	-0.558874111	0.04138737			
Runx2	-0.553925249	0.005752456			

Map3k1	-0.553237047	0.015292886			
Hoxd11	-0.545265262	0.03108183			
Cdk5r1	-0.536912683	0.047942281			
Etv4	-0.528936756	0.030032435			
Prkcb	-0.523715505	0.011987196			
Gnb2l1	-0.5156446	0.033484625			
Hspa8	-0.513256257	0.04664049			
Gabra5	-0.500966618	0.010057275			

Supplemental Table 5. Age and sex of MS patients and healthy volunteers

Sample code	Final diagnosis	Sex	Age	Disease duration	EDSS	sampling-context	treatment
MS1	relapsing-remitting MS, RRMS	Female	57	1 year	1	relapse	Methylprednisolone+ Dimethyl fumarate
MS2	relapsing-remitting MS, RRMS	Female	38	16 years	3	remission	Methylprednisolone
MS3	relapsing-remitting MS, RRMS	Male	34	3 years	2	relapse	Methylprednisolone+ Teriflunomide
MS4	relapsing-remitting MS, RRMS	Female	37	1 year	6	relapse	Methylprednisolone+ Mycophenolate Mofet
MS5	relapsing-remitting MS, RRMS	Female	32	2 years	2	relapse	Methylprednisolone+ Siponimod
MS6	relapsing-remitting MS, RRMS	Male	44	6 months	2	relapse	Ofatumumab
MS7	relapsing-remitting MS, RRMS	Male	50	3 years	2	relapse	Methylprednisolone
MS8	relapsing-remitting MS, RRMS	Male	35	4 years	3	relapse	Methylprednisolone
MS9	relapsing-remitting MS, RRMS	Male	56	3.5years	6	relapse	Methylprednisolone+ Ofatumumab
MS10	relapsing-remitting MS, RRMS	Female	30	3 months	1.5	remission	Methylprednisolone+ Ofatumumab
MS11	Clinically Isolated Syndrome	Female	26	1 month	1	Acute episode	Methylprednisolone
Ctrl1	healthy	Female	40				
Ctrl2	healthy	Female	38				
Ctrl3	healthy	Male	34				
Ctrl4	healthy	Male	44				
Ctrl5	healthy	Female	32				
Ctrl6	healthy	Male	37				
Ctrl7	healthy	Male	30				
Ctrl8	healthy	Female	41				
Ctrl9	healthy	Male	36				
Ctrl10	healthy	Female	49				
Ctrl11	healthy	Male	33				

Supplemental Table 6. The sequences of sgRNAs

**sgRNA**	**Sequence(5' -> 3')**
sgRIPK3-1	GGTCACTGAGGACCTCCAGT
sgRIPK3-2	GTGTAGGAAGAAGATATCCT
sgRIPK3-3	CAGCTTTGGGATCCTCGTGT
sgMLKL-1	GCGCAGCCTCCTAGTCCTGA
sgMLKL-2	TCAGCAAGAAGTCCCATATT
sgMLKL-3	ATGTCAGCCAGCCAGCATCC
sgOGA-1	GATCCACAGAGCTCATCCCA
sgOGA-2	TTGGCGAGAGATGTATTCAG
sgOGA-3	AGATGCTACGGGAATTCCAG
sgNC	GCGAGGTATTCGGCTCCGCG

Supplemental Table 7. The sequences of siRNAs

Gene	Sense (5' -> 3')	Antisense (5' -> 3')
Ager	GGAAGGAGGUCAAGUCCAAUU	UUGGACUUGACCUCCUUCCUU
Apob	AGGCAGAGCUAGUGGCAAAUU	UUUGCCACUAGCUCUGCCUUU
Axl	GAUCAGAGCUGGAGGAUUUUU	AAAUCCUCCAGCUCUGAUCUU
Becn1	GCUGGAGUUGGAUGACGAAUU	UUCGUCAUCCAACUCCAGCUU
Bmprla	GCUACGCAGGACAAUAGAAUU	UUCUAUUGUCCUGCGUAGCUU
Cacnb4	GGAGAAUAUUCGGAUUCAAUU	UUGAAUCCGAAUAUUCUCCUU
Ccr5	GAGAGAAGUUCCGGAGUUAUU	UAACUCCGGAACUUCUCUCUU
Cldn3	CCUACGACCGCAAGGACUAUU	UAGUCCUUGCGGUCGUAGGUU
Cxcr2	GAGAAUUCAAGGUGGAUAAUU	UUAUCCACCUUGAAUUCUCUU
Gabrg2	AAGCAAGGAUAAAGACAAAUU	UUUGUCUUUAUCCUUGCUUUU
Hdac1	GAAAGAUCCUGAAGAGAAAUU	UUUCUCUUCAGGAUCUUUCUU
Ifng	GCAUAGAUGUGGAAGAAAAUU	UUUUCUUCCACAUCUAUGCUU
Insr	GCACAGGCCAUUUGAGAAAUU	UUUCUCAAAUGGCCUGUGCUU
Irs1	GAGAAGAAGUGGCGGCACAUU	UGUGCCGCCACUUCUUCUCUU
Krt8	GUACAGAGAUGGAGAAUGAUU	UCAUUCUCCAUCUCUGUACUU
Mpo	UACAAGGAGCGGAGAGAAAUU	UUUCUCUCCGCUCCUUGUAUU
Nefh	GAUAAAGGUAGUAGAGAAAUU	UUUCUCUACUACCUUUAUCUU
Nox4	GCUUCUACCUACGCAAUAAUU	UUAUUGCGUAGGUAGAAGCUU
Pgm1	GGACCAUAUCCGAGAGAAAUU	UUUCUCUCGGAUAUGGUCCUU
Ptger4	CCACAUACAUGAAGGGCCAUU	UGGCCCUUCAUGUAUGUGGUU
Slc12a2	GCAAGAAGCCCAAGGGUUUUU	AAACCCUUGGGCUUCUUGCUU
Slit1	GGAGUUGAACGGUAUCAAAUU	UUUGAUACCGUUCAACUCCUU
Slit2	GGGAGAGAAGUGUGAGAAAUU	UUUCUCACACUUCUCUCCCUU
Smad1	UGAAGAAACUGAAGAAGAAUU	UUCUUCUUCAGUUUCUUCAUU
Sox17	GCACGGAAUUCGAACAGUAUU	UACUGUUCGAAUUCCGUGCUU
Tgm2	GAACAAACUGGCAGAGAAAUU	UUUCUCUGCCAGUUUGUUCUU

Tubb4a	CUGAAGAGGAGGUGGCUUAUU	UAAGCCACCUCCUCUUCAGUU
Ripk3	UGACAAGAUUCAUGGAGAAUU	UUCUCCAUGAAUCUUGUCAUU
Xpo1	AGCAAUUAUGACAAUGUUAUU	UAACAUUGUCAUAAUUGCUUU
Ctla4	GCGGCAGACAAAUGACCAAUU	UUGGUCAUUUGUCUGCCGCUU
Il10	GCUGGACAACAUACUGCUAUU	UAGCAGUAUGUUGUCCAGCUU
NC	UUCUCCGAACGUGUCACGUTT	ACGUGACACGUUCGGAGAATT

Supplemental Table 8. Primer sequences used for qPCR

**Primer**	**Forward (5' -> 3')**	**Reverse (5' -> 3')**
Il1α	CGAAGACTACAGTTCTGCCATT	GACGTTTCAGAGGTTCTCAGAG
Ifng	ATGAACGCTACACACTGCATC	CCATCCTTTTGCCAGTTCCTC
Tnf	CCCTCACACTCAGATCATCTTCT	GCTACGACGTGGGCTACAG
Ccl5	GCTGCTTTGCCTACCTCTCC	TCGAGTGACAAACACGACTGC
Hprt	TCAGTCAACGGGGGACATAAA	GGGGCTGTACTGCTTAACCAG
Il1β	GCAACTGTTCCTGAACTCAACT	ATCTTTTGGGGTCCGTCAACT
Ptgs2	CAGGCTGAACTTCGAAACA	GCTCACGAGGCCACTGATACCTA
Nos2	TCCCAGCCTGCCCCTTCAAT	CGGATCTCTCTCCTCCTGGG
Cd86	AACGTATTGGAAGGAGATTACAGCT	CCTGCTAGGCTGATTCGGCT
Tmem119	GTGTCTAACAGGCCCCAGAA	AGCCACGTGGTATCAAGGAG

Supplemental Table 9. Reagents

REAGENT or RESOURCE	SOURCE	IDENTIFIER
**Antibodies**		
Rabbit monoclonal anti-mouse MBP	Cell Signaling Technology	Cat# 78896; RRID: AB_2799920
PE anti-mouse CD4	Biolegend	Cat# 100408; RRID: AB_312693
Brilliant Violet 605™ anti-mouse CD4	Biolegend	Cat# 100548; RRID: AB_2563054
FITC Conjugated Rat anti-mouse CD4	Cell Signaling Technology	Cat# 96127; RRID: AB_2943473
FITC anti-mouse CD45.1	Invitrogen	Cat# 11-0453-82; RRID: AB_465058
PE anti-mouse CD45.2	Invitrogen	Cat# 12-0454-82; RRID: AB_465678
APC anti-mouse CD45.1	Biolegend	Cat# 110713; RRID: AB_313502
APC anti-mouse CD45.2	Biolegend	Cat# 109813; RRID: AB_389210
PE/Cyanine7 anti-mouse CD4	Biolegend	Cat# 100422; RRID: AB_312707
IFN gamma Monoclonal Antibody	Invitrogen	Cat# 16-7311-81; RRID: AB_469242
IL-4 Monoclonal Antibody	Invitrogen	Cat# 16-7041-81; RRID: AB_469208
APC anti-mouse IFN gamma	Biolegend	Cat# 113605; RRID: AB_2734170
Rabbit monoclonal anti-mouse Foxp3	Cell Signaling Technology	Cat# 12653; RRID: AB_2797979
PE anti-mouse IL-17A	Biolegend	Cat# 506903; RRID: AB_315463
Rabbit monoclonal anti-mouse IBA-1	Abcam	Cat# ab178847; RRID: AB_2832244
Mouse monoclonal anti-mouse GFAP	Santa Cruz Biotechnology	Cat# sc-33673; RRID:AB_627673
PE/Cyanine7 anti-mouse CD3	Biolegend	Cat# 100220; RRID: AB_1732057
Phospho-RIP3 (Thr231/Ser232) (E7S1R) rabbit mAb	Cell Signaling Technology	Cat# 91702; RRID: AB_2937060
Phospho-MLKL (Ser345) (D6E3G) Rabbit mAb	Cell Signaling Technology	Cat# 37333; RRID: AB_2799112
Phospho-IKKα/β (Ser176/180) (16A6) Rabbit mAb	Cell Signaling Technology	Cat# 2697; RRID: AB_2079382
Cleaved Caspase-3 (Asp175) Antibody	Cell Signaling Technology	Cat# 9661; RRID: AB_2341188
MLKL (D6W1K) Rabbit mAb	Cell Signaling Technology	Cat# 37705; RRID: AB_2799118
Phospho-RIP (Ser166) Antibody	Cell Signaling Technology	Cat# 53286; RRID:AB_2925183
RIP (D94C12) XP Rabbit mAb	Cell Signaling Technology	Cat# 3493; RRID: AB_2305314
RIP3 (D4G2A) Rabbit mAb	Cell Signaling Technology	Cat# 95702; RRID: AB_2721823

Glut1 (E4S6I) Rabbit mAb	Cell Signaling Technology	Cat# 73015; RRID: AB_3064908
Mouse monoclonal anti-mouse A20	Santa Cruz Biotechnology	Cat# sc-166692; RRID: AB_2204516
Beta Actin antibody	Proteintech	Cat# 66009-1-Ig; RRID: AB_2687938
Mouse monoclonal anti-mouse O-GlcNAc	Thermo Fisher Scientific	Cat# MA1-072; RRID: AB_326364
Brilliant Violet 650™ anti-mouse CXCR3	Biolegend	Cat# 126531; RRID: AB_2563160
Brilliant Violet 421™ anti-mouse CCR4	Biolegend	Cat# 131217; RRID: AB_10933253
PE/Cyanine7 anti-mouse CCR6	Biolegend	Cat# 129815; RRID: AB_1877244
FITC anti-mouse CD25	Biolegend	Cat# 102005; RRID: AB_312854
IL-2 Monoclonal Antibody	Biolegend	Cat# 16-7021-85; RRID: AB_657802
Rabbit monoclonal anti-mouse TMS1/ASC	Abcam	Cat# ab309497; RRID: AB_3669034
FITC anti-mouse PD-1	Biolegend	Cat# 135213; RRID: AB_10689633
Alexa Fluor 647 anti-MU/HU GL7	Biolegend	Cat# 144605; RRID: AB_2562184
APC anti-mouse CD138	Biolegend	Cat# 142505; RRID: AB_10960141
APC anti-mouse CXCR5	Biolegend	Cat# 145505; RRID: AB_2561969
Alexa Fluor 488 anti-mouse Ki-67	Biolegend	Cat# 652417; RRID: AB_2564236
Alexa Fluor 647 anti-mouse Ki-67	Biolegend	Cat# 151206; RRID:AB_2566801
Anti-human CD127	Polaris Biology	cat# H03612094
Anti-human CD194/CCR4	Polaris Biology	cat# H09521095
Anti-human CD196/CCR6	Polaris Biology	cat# H09711096
Anti-human CD20	Polaris Biology	cat# H10612097
Anti-human IgD	Polaris Biology	cat# H32011098
Anti-human CD27	Polaris Biology	cat# H14923099
Anti-human CD11b	Polaris Biology	cat# H03025105
Anti-human CD57	Polaris Biology	cat# H23542100
Anti-human CD28	Polaris Biology	cat# H15911107
Anti-human CD279/PD-1	Polaris Biology	cat# H15811101
Anti-human HLA-DR	Polaris Biology	cat# H31111111
Anti-human CD8	Polaris Biology	cat# H25754109
Anti-human CD45RA	Polaris Biology	cat# H21411112
Anti-human CD123/IL-3R	Polaris Biology	cat# H03311110
Anti-human CD294/CRTH2	Polaris Biology	cat# H16511115
Anti-human CD183/CXCR3	Polaris Biology	cat# H08912116
Anti-human CD11c	Polaris Biology	cat# H03112118
Anti-human CD16	Polaris Biology	cat# H06767120
Anti-human CD19	Polaris Biology	cat# H09212119
Anti-human CD24	Polaris Biology	cat# H13132124
Anti-human CD185/CXCR5	Polaris Biology	cat# H09131125

Anti-human CD25/IL-2Rα	Polaris Biology	cat# H13553126
Anti-human CD14	Polaris Biology	cat# H04842127
Anti-human CD66b	Polaris Biology	cat# H24832128
Anti-human CD56	Polaris Biology	cat# H23414131
Anti-human CD197/CCR7	Polaris Biology	cat# H09813132
Anti-human CD4	Polaris Biology	cat# H20631133
Anti-human CD38	Polaris Biology	cat# H20412134
Anti-human CD45	Polaris Biology	cat# H21231138
Anti-human Granzyme B	Polaris Biology	cat# H30723139
Anti-human Perforin	Polaris Biology	cat# H37312140
Anti-human TCRγδ	Polaris Biology	cat# H40762141
Anti-human CD3	Polaris Biology	cat# H16712189
Anti-human Phospho-IKK alpha/beta	Thermo Fisher Scientific	cat# 701643; RRID: AB_2532498
Cleaved Caspase-3 (Asp175) (D3E9) Rabbit mAb	Cell Signaling Technology	cat# 74860SF
Phospho-RIPK1 (Ser166) (D8I3A) Rabbit mAb	Cell Signaling Technology	cat# 96323SF
Anti-MLKL (phospho S358)	Abcam	cat# AB208909
Human RIP1 Rabbit mAb	Biolynx	cat# YJY-5-8
**Chemicals, peptides, and recombinant proteins**		
7-AAD Viability Staining Solution	Thermo Fisher Scientific	Cat# 00-6993-50
Pertussis Toxin	Absin	Cat# abs42024900
Myelin Oligodendrocyte Glycoprotein (35-55)	Hanhong Technology	Cat# 332P2372
Neomycin	Sigma	Cat# N6386-5G
Polymyxin B sulfate	Adamas	Cat# 01380785
Diphtheria Toxin	Sigma	Cat# D0564
IFN-gamma	MedChemExpress	Cat# HY-P70667
10X RBC Lysis Buffer	Thermo Fisher Scientific	Cat# 00-4300-54
Cell Stimulation Cocktail	Thermo Fisher Scientific	Cat# 00-4975-03
Necrostatin 2 racemate(Nec-1s)	Selleck	Cat# S8641
GSK-872	MedChemExpress	Cat# HY-101872
Recombinant Mouse TNF alpha	Novoprotein	Cat# CF09
CellTrace™ Violet	Thermo Fisher Scientific	Cat# C34571
Thiamet-G	Selleck	Cat# S7213
Z-VAD-FMK	Selleck	Cat# S7023
SYTOX™ Deep Red	Thermo Fisher Scientific	Cat# S11381

ImunoSep™ Cell Separation Buffer	Precision BioMedicals	Cat# 604025
ImunoSep™ Separator	Precision BioMedicals	Cat# 602005
Hematoxylin-eosin (H&E)	Sevier Biotech	Cat# G1076
PAS	Sevier Biotech	Cat# G1008
X-VIVO™ 15 Serum-free Hematopoietic Cell Medium	Lonza	Cat# 04-418Q
Recombinant Murine IL-12	Peprotech	Cat# 210-12
Recombinant Murine IL-2	Peprotech	Cat# 212-12
Recombinant Murine IL-4	Peprotech	Cat# 214-14
Recombinant Murine IL-6	Peprotech	Cat# 216-16
Recombinant Human IL-23	Peprotech	Cat# 200-23
Recombinant Murine IL-1β	Peprotech	Cat# 211-11B
Recombinant Human TGF-β1	Peprotech	Cat# 100-21
**Critical commercial assays**		
Mouse CD4^{+} T-Cell Isolation Kit	Selleck	Cat# B90001
T Cell Activation/Expansion Kit, mouse	Miltenyi	Cat# 130-093-627
EasySep Mouse Naive CD4^{+} T Cell Isolation Kit	STEMCELL	Cat# 19765
ProteanFect™ Max Mouse Immunocyte Transfection Kit	NANOPORTAL BIOTECH	Cat# PT03
LDH Cytotoxicity Assay Kit	Beyotime	Cat# C0017
Duolink® In Situ Red Starter Kit Mouse/Rabbit	Sigma	Cat# DUO92101-1KT
**Biological samples**		
Human blood samples	This paper	NA
**Organisms/strains**		
Mouse: C57BL/6 (CD45.2)	Lingchang Biotechnology	NA
Mouse: C57BL/6 (CD45.1)	Shanghai Model Organisms center	Cat# NM-KI-210226; RRID: IMSR_NM-KI-210226
Mouse: Foxp3-IRES-DTRGFP	Shanghai Model Organisms center	Cat# NM-KI-190046; RRID:IMSR_NM-KI-190046
Mouse: Rag2-KO	Shanghai Model Organisms center	Cat# NM-KO-190429; RRID: IMSR_NM-KO-190429
Mouse: R26-EGFP(M-NSG)	Shanghai Model Organisms center	Cat# NM-KI-232214; RRID: IMSR_NM-KI-232214
Mouse: B6(C)-*H2-Ab1*^{bm12}/KhEgJ	The Jackson Laboratory	Cat# 001162; RRID: IMSR_JAX:001162
Mouse: R26-CAG-Cas9	Shanghai Model	Cat# NM-KI-00120; RRID:

	Organisms center	IMSR_NM-KI-00120
Mouse: Foxp3-IRES-tdTomato-2A-CreERT2	Shanghai Model Organisms center	Cat# NM-KI-190120; RRID: IMSR_NM-KI-190120
