# Supplementary material for: Enhancing Treg persistence by inhibiting necroptosis restrains autoimmune pathology
Source: J Clin Invest. 2026 Sep 1;136(21):e207077. doi: 10.1172/JCI207077 (PMC13722395; doi:10.1172/JCI207077)

Figure 6A

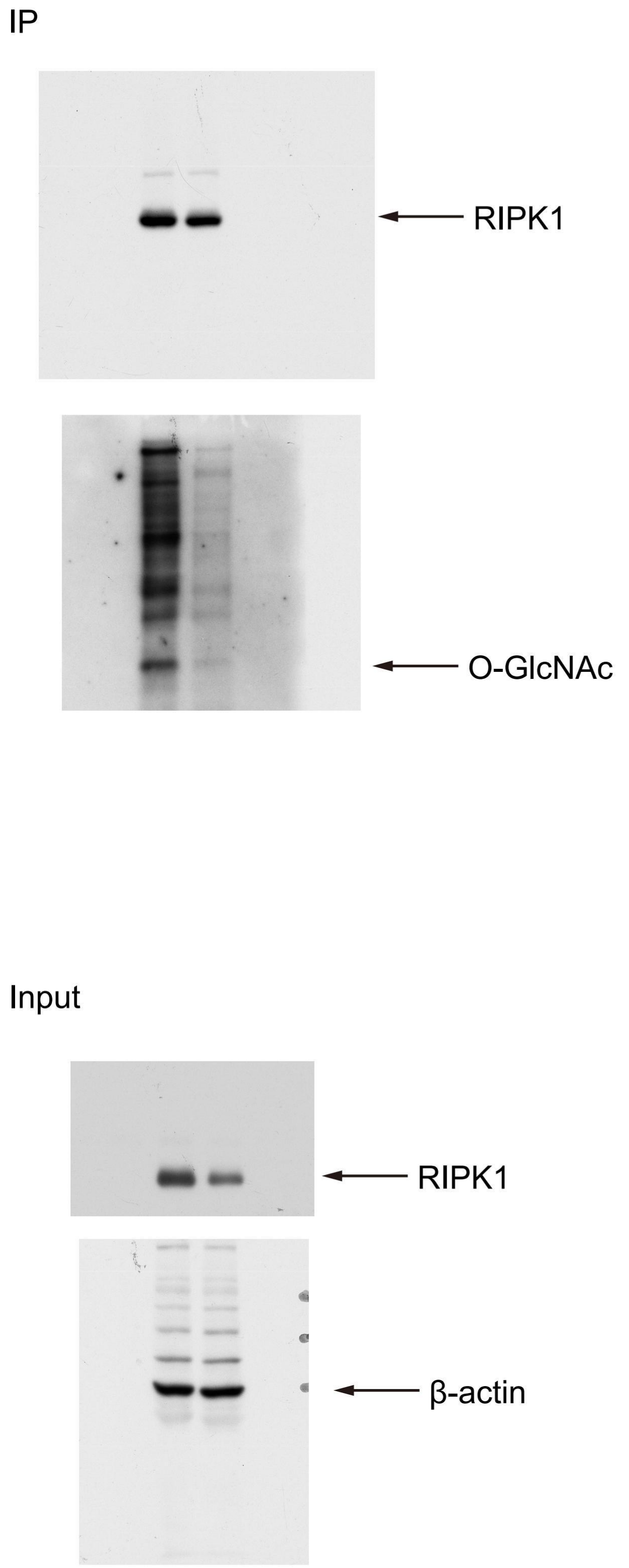

Supplemental Figure 5A

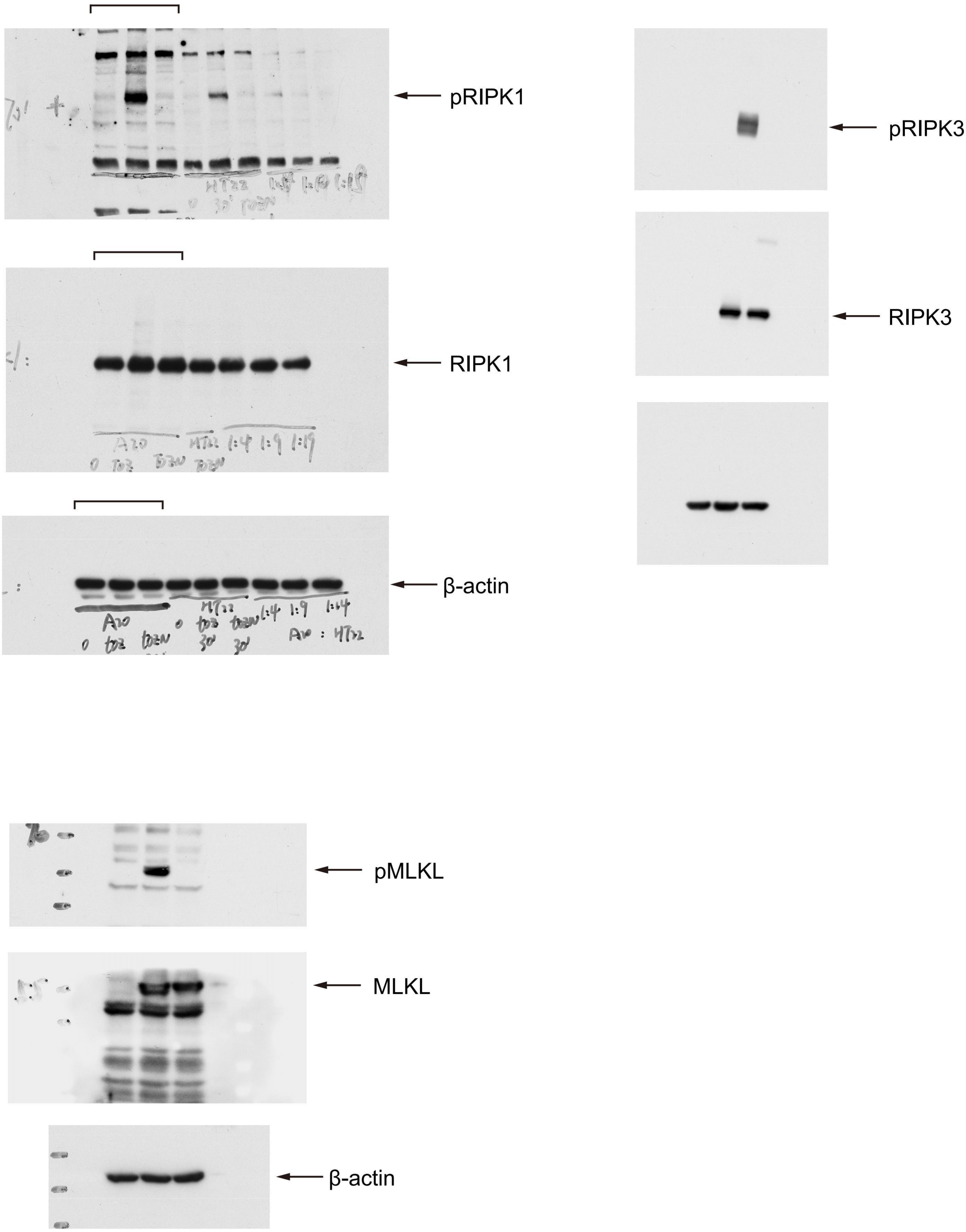

Supplemental Figure 16D

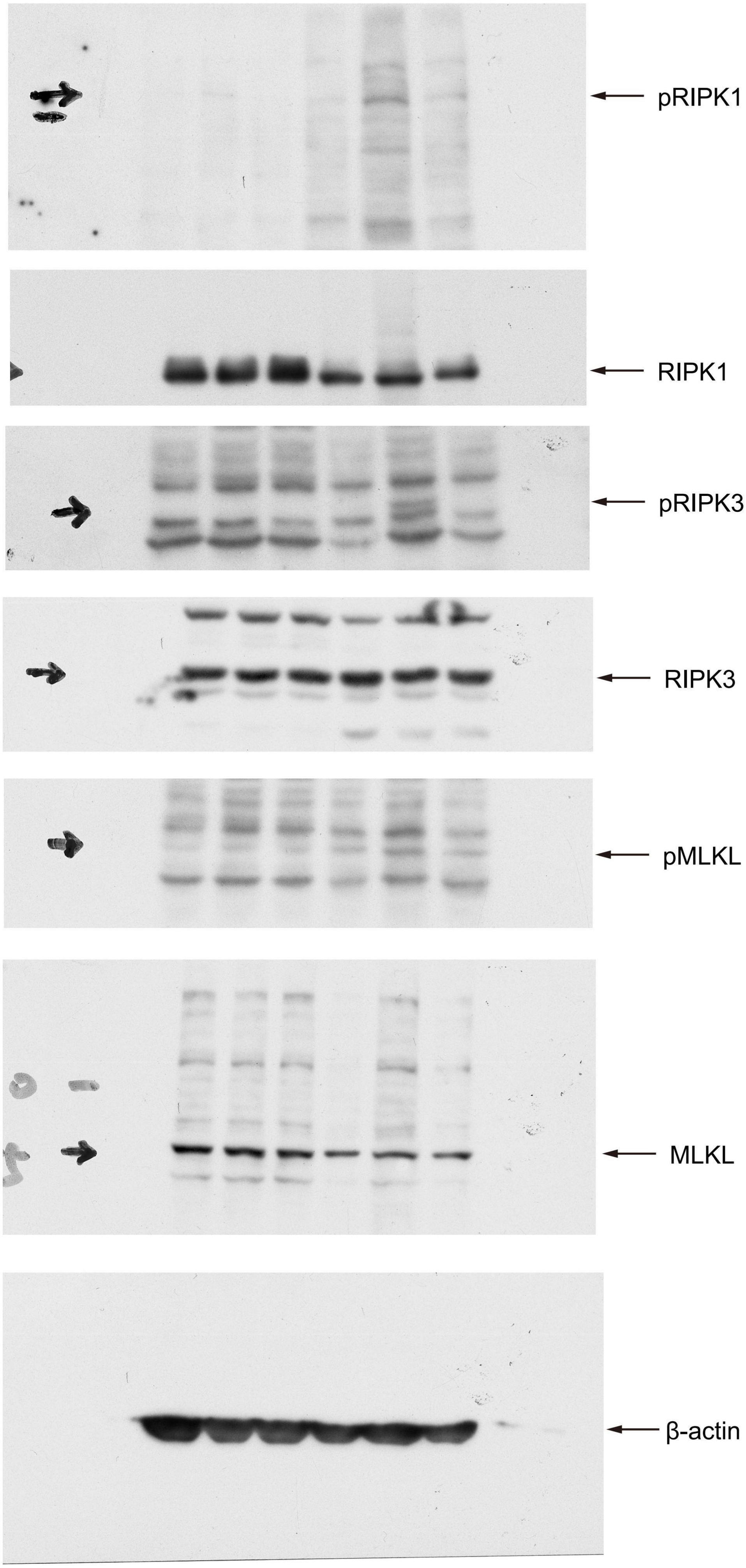

Supplemental Figure 17A

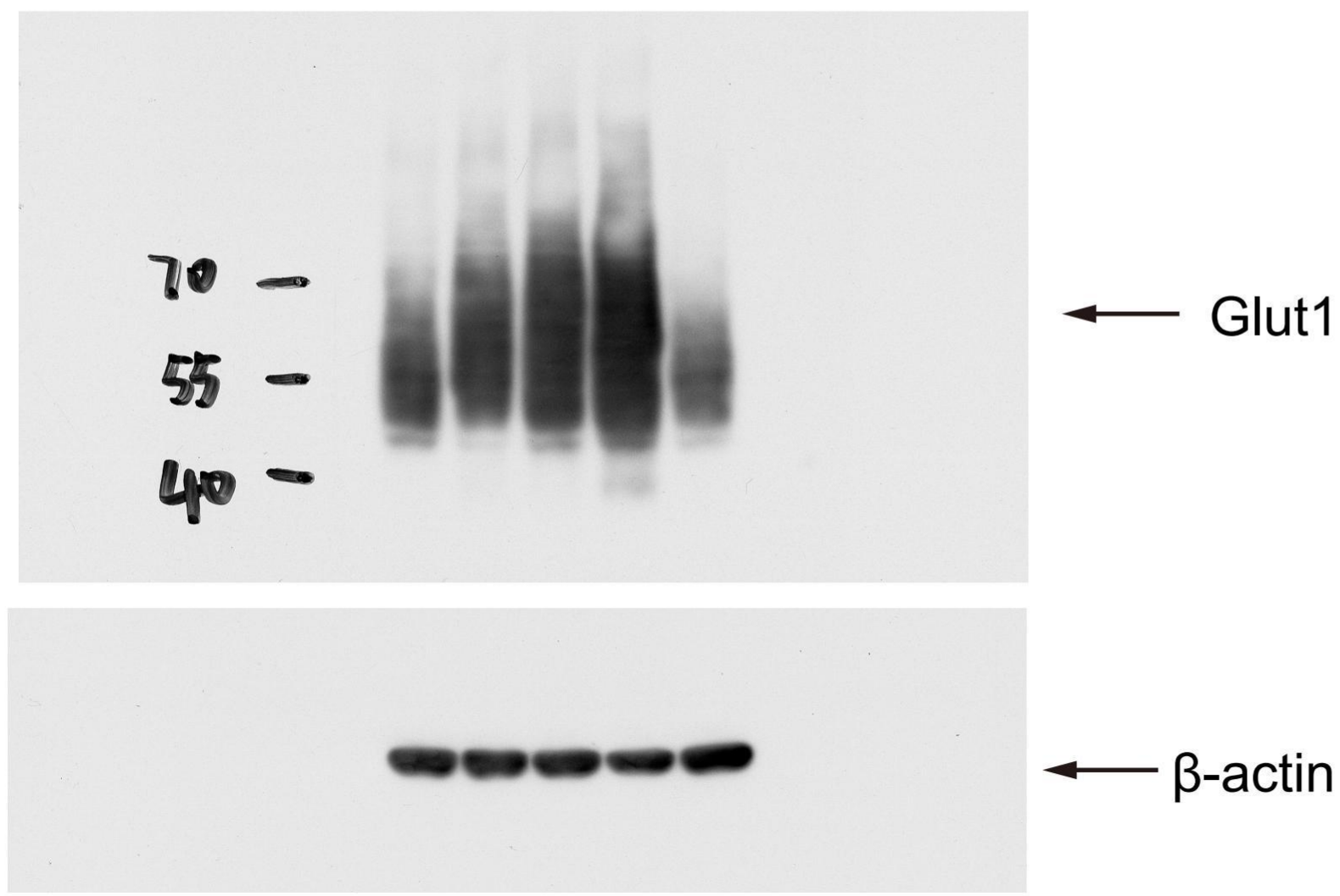

Supplemental Figure 17D

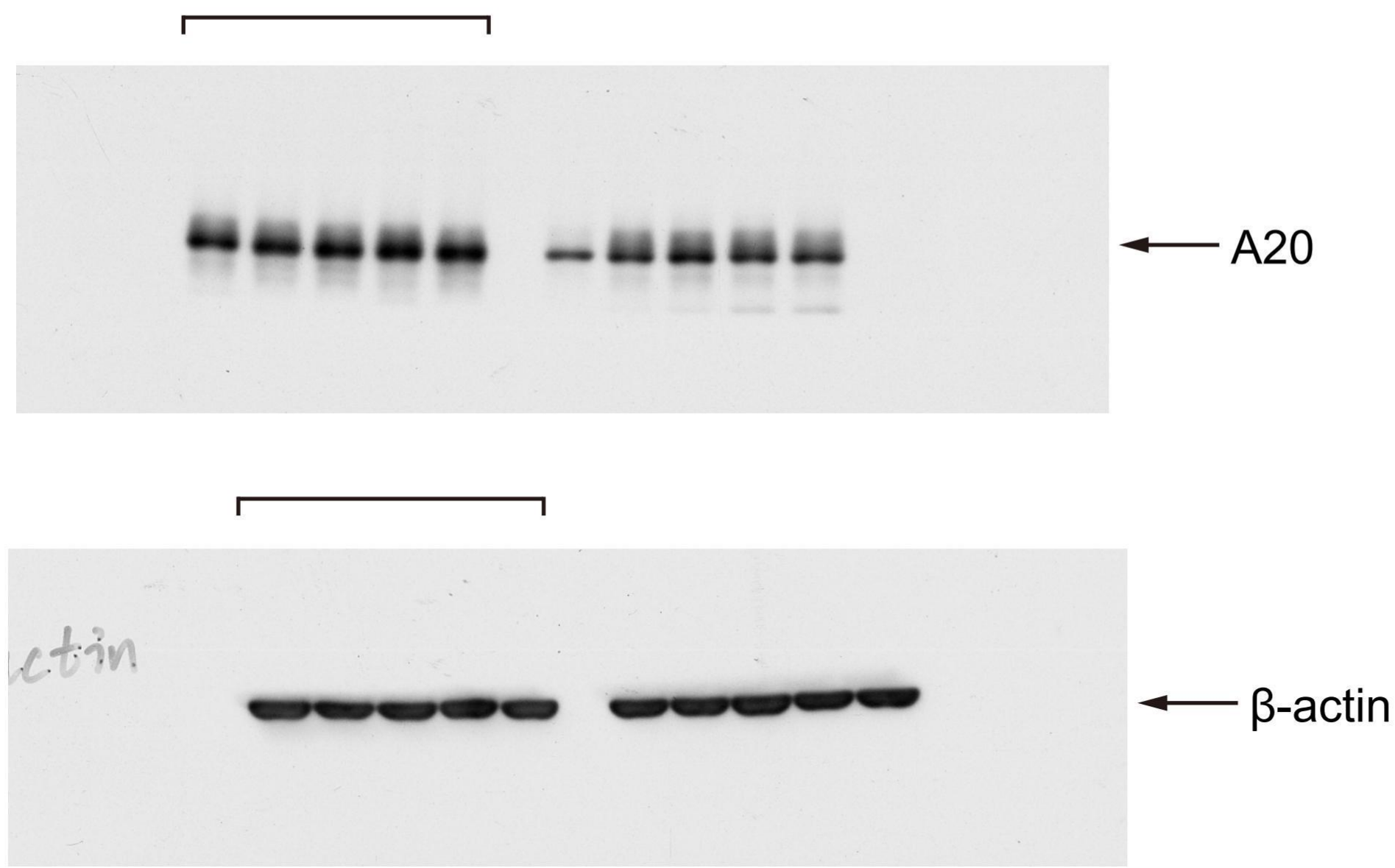

Supplemental Figure 17I

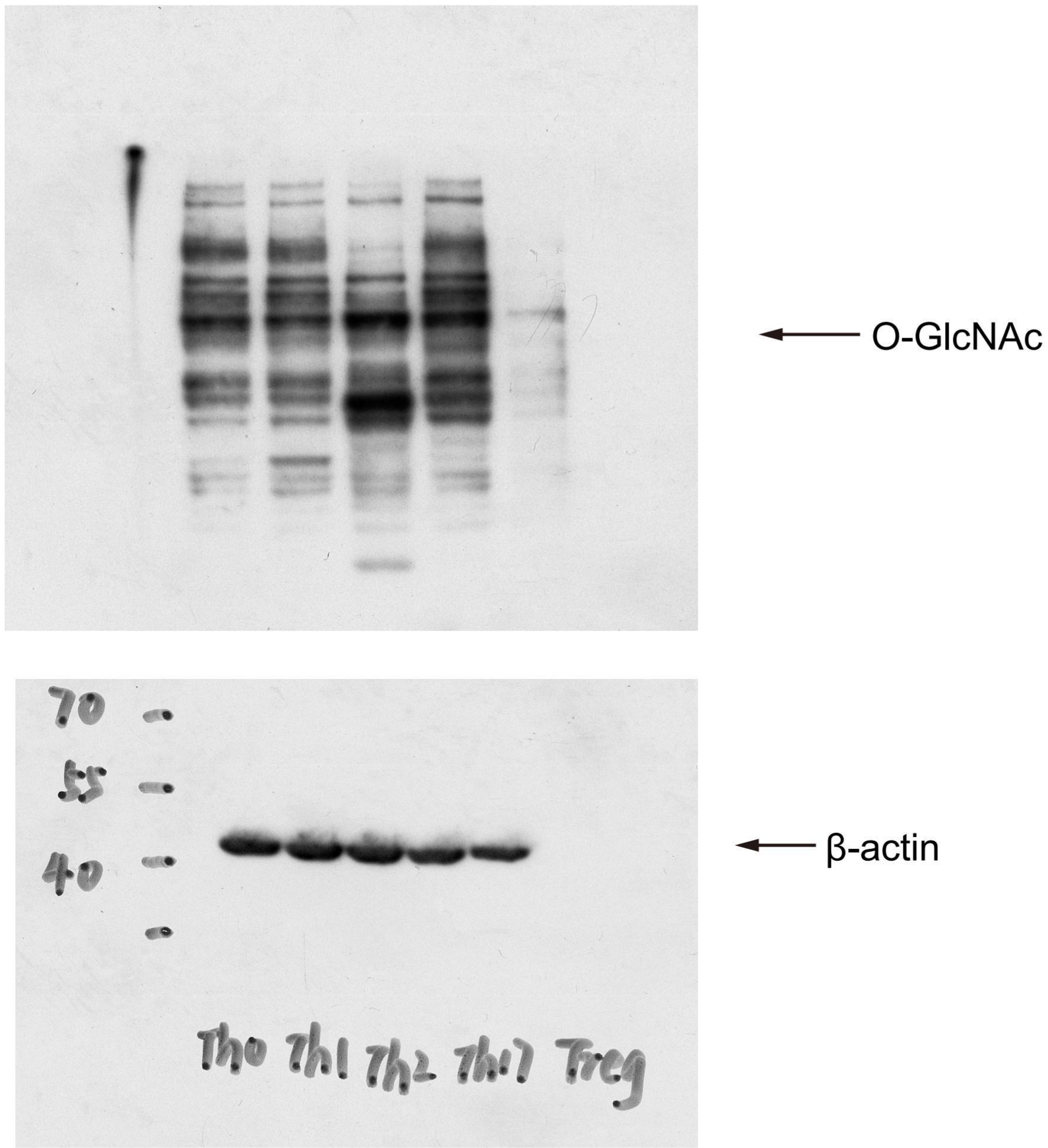

Supplemental Figure 18B

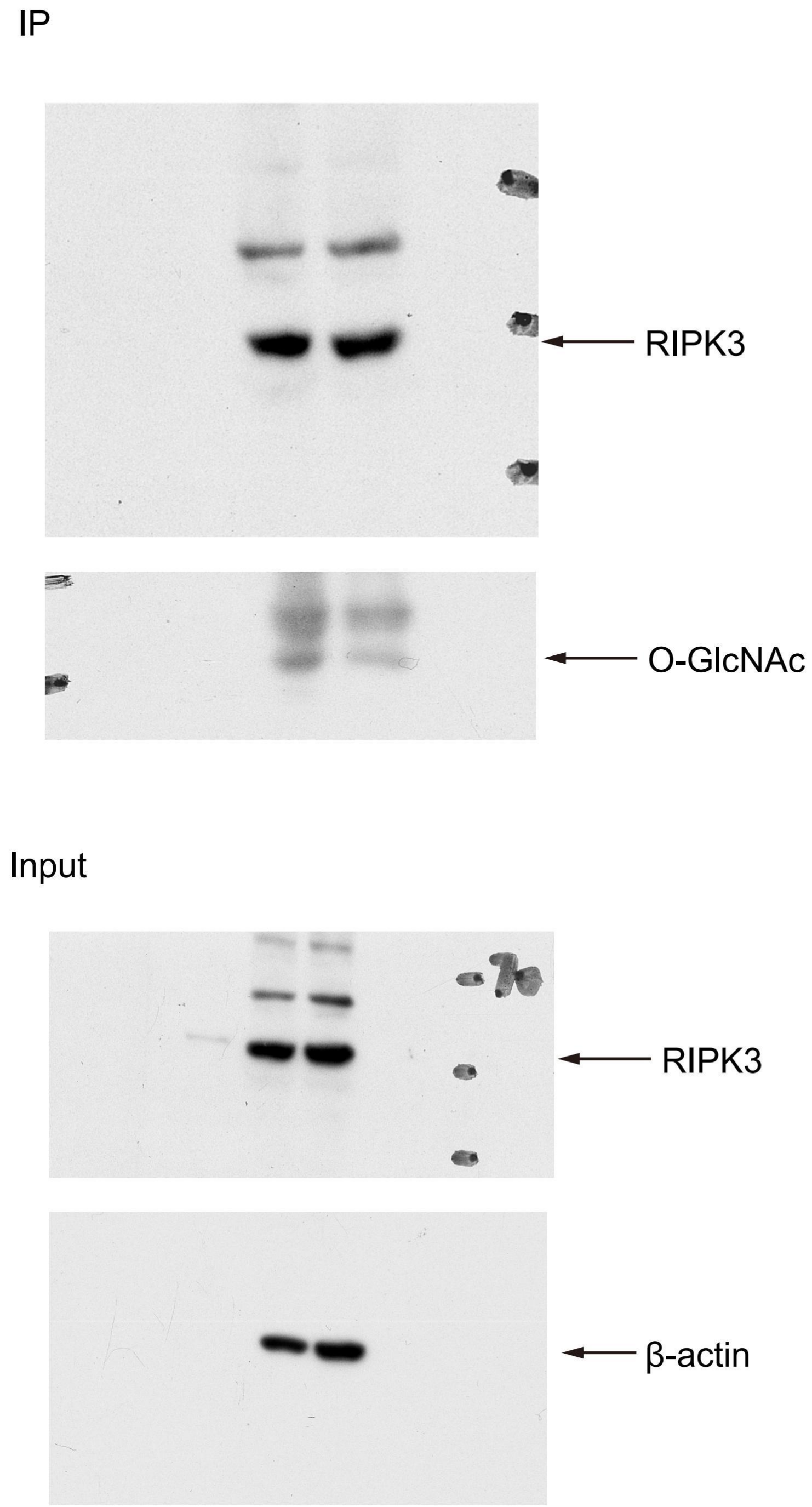

Supplemental Figure 19C

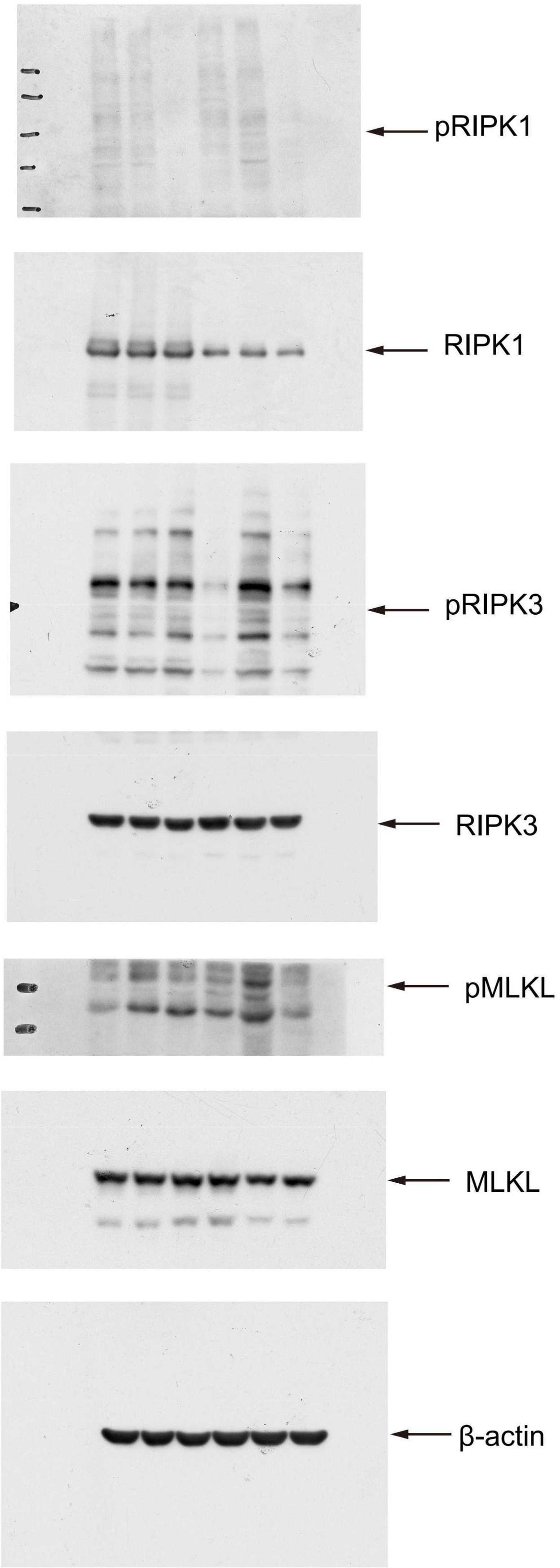

Supplemental Figure 22C

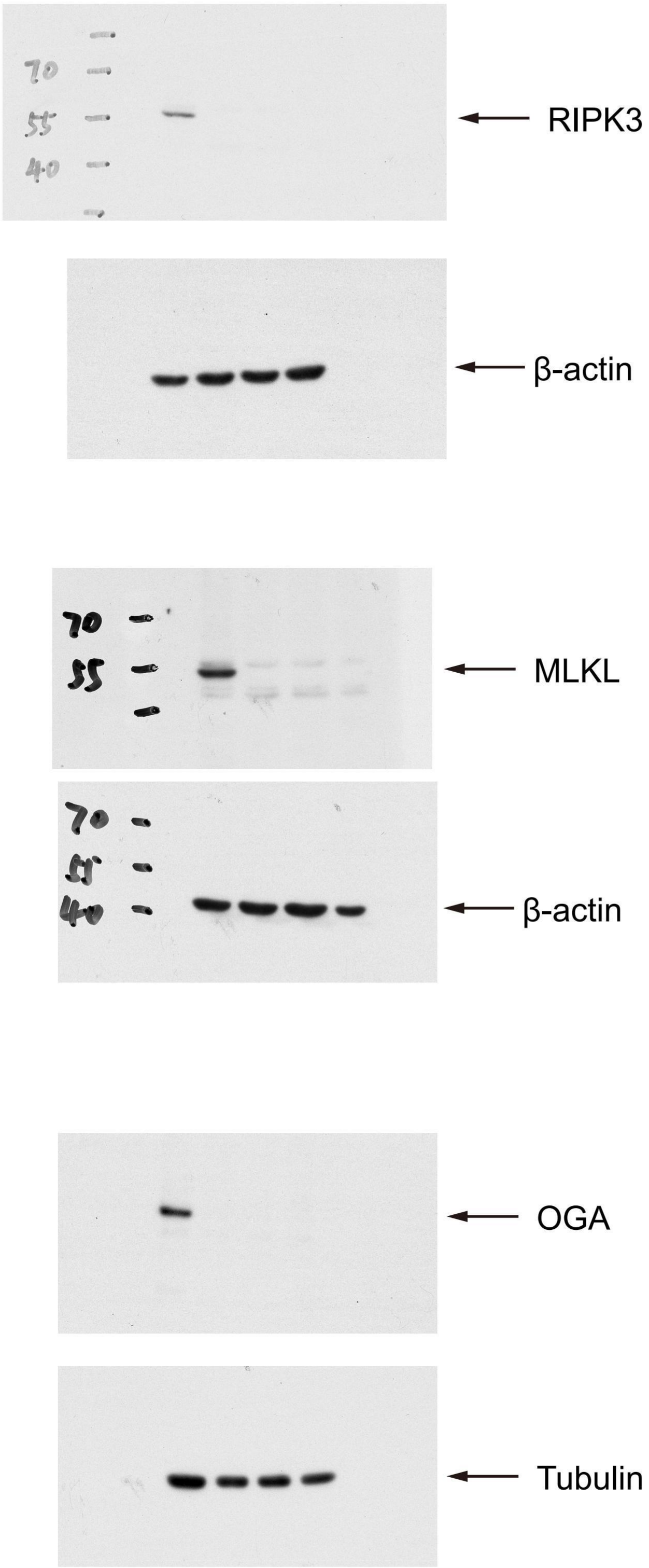

Supplement: Supplementary file 2 [file jci-136-207077-s213.pdf]
